# Exploring Recent Progress in First-Row Trimetallic Nanostructures and Their Derivatives for Electrocatalytic Water Splitting: A Comprehensive Review

**DOI:** 10.1021/acsomega.5c05855

**Published:** 2025-12-03

**Authors:** Fahimeh Sadat Vajedi, Nakédia M. F. Carvalho

**Affiliations:** Instituto de Química, 28130 Universidade do Estado do Rio de Janeiro (UERJ), Rua São Francisco Xavier, 524, Rio de Janeiro, 20550-900 Rio de Janeiro Brasil

## Abstract

Present scientific efforts are heavily concentrated on enhancing energy storage and conversion technologies to reduce environmental degradation and tackle impending energy issues. Electrocatalytic water splitting emerges as a leading method for producing pure hydrogen without generating undesired byproducts, highlighting the need for robust, cost-effective, highly active, and earth-abundant electrocatalyst materials composed of non-noble metals that exhibit excellent stability and performance for both hydrogen evolution reaction (HER) and oxygen evolution reaction (OER) at low overpotentials and high efficiencies. In this context, the design and engineering of trimetallic nanostructured materials with diverse architectures emerge as a promising strategy for energy conversion electrocatalysis. These trimetallic nanostructures, particularly those incorporating first-row transition metals, exhibit distinctive physicochemical properties, heightened efficacy, and enhanced durability across diverse applications compared to mono- and bimetallic counterparts, driven by synergistic interactions among the trimetals. Moreover, the incorporation of additional metals into the secondary building units (SBUs) of frameworks represents an efficacious strategy for augmenting electrochemical performance and electrical conductivity, increasing active site exposure, enhanced charge capacity, and proficient charge transfer among distinct ions. In this review, fundamental concepts and key evaluation metrics for electrochemical water-splitting reactions are outlined. Subsequently, an overview of recent advancements in the synthesis, structural/chemical modifications, and utilization of first-row transition metals as multifunctional nanomaterials for overall water splitting is elucidated. Then, a comprehensive analysis is provided on various trimetallic catalyst categories based on first-row transition metals, encompassing alloys, oxides, hydroxides, nitrides/phosphides/sulfides, and composite structures, aiming to expand the understanding of trimetallic systems and delineate a roadmap for the integration of diverse trimetallic materials as advanced candidates in electrochemical energy storage and conversion technologies.

## Graphical abstract

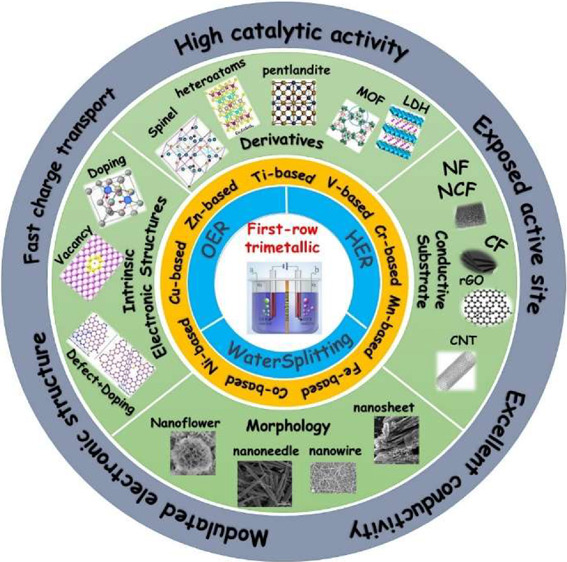

## 1 Introduction

Increasing energy demand in a world with decreasing reserves of fossil fuels has impelled a compelling imperative for alternative and sustainable energy sources. The environmental issues that have resulted from them, such as air pollution, climate change, and sea level rise, have increased the focus on alternative energy sources such as wind, solar, tidal, biomass, biodiesel, wave, and nuclear power to address the energy demand puzzle.^1–7^ Their variability due to geographical and environmental factors, however, poses a challenge to a consistent energy supply. There is therefore a fundamental imperative to develop measures to deliver a consistent and effective conversion of renewable sources to sustainable energy resources.

Hydrogen, as the most calorific energy carrier (142 MJ kg^–1^) and a potential candidate to alleviate the adverse impact of environmental pollution and global climate change, has gained much attention recently.^2,3^ Currently, there are various methodologies used for hydrogen production, including water electrolysis, photolysis,^4^ thermolysis,^5^ hydrocarbon steam reforming,^6^ biomass pyrolysis,^7^ and coal gasification.^2^ Within this landscape, electrochemical approaches, particularly water splitting, have gained prominence due to their low environmental impact and minimal greenhouse gas emissions, signifying their importance in the development of efficient energy storage and conversion systems.^8^

Electrochemical water splitting has emerged as a promising technology for hydrogen production, effectively converting intermittent-renewable resources like solar and wind energy into a stable chemical energy source in the form of hydrogen.^9^ Of noteworthy consideration is the fact that, despite its ideal reliance on inexpensive and renewable electricity sources, water electrolysis is regarded as a promising avenue for hydrogen production due to its environmental friendliness, abundance, and renewability of its sole reactant water.^10,11^ Effecting the conversion of energy from renewable sources necessitates the utilization of specialized materials characterized by precise control over their shape, size, porosity, crystalline phases, and composition. Addressing these challenges hinges on the development of nonprecious nanomaterial systems and technologies designed to harness and transform renewable energy sources.^12,13^ Consequently, substantial attention is directed toward the exploration of electrode materials designed for water splitting applications, particularly for the corresponding half-reactions oxygen evolution reaction (OER) and hydrogen evolution reaction (HER) electrocatalysts to reduce overpotential and improve their kinetics.

Noble-metal-based electrocatalysts, exemplified by materials like Ir, Ru, Pt and their respective oxides, offer excellent performance for OER and HER, but their applicability for large-scale commercialization is hampered by constraints such as high cost, scarcity, and reduced stability due to contamination and aggregation under demanding conditions.^14,15^ Consequently, the development of cost-effective, stable, and efficient electrocatalysts using earth-abundant materials is essential for advancing water splitting technologies.^16,17^ OER electrocatalysts for acidic media face challenges of high cost and limited availability, requiring acid-resistant and stable materials for practical applications. Additionally, the use of earth-abundant electrocatalysts in an alkaline environment holds the potential to simplify and reduce the cost of water electrolyzer systems.^18–20^ Developing such catalysts with markedly elevated activity, operating at minimal overpotential (η), and robust stability in alkaline media is essential for advancing economical hydrogen production and addressing the limitations of existing technologies.

Generally, water splitting catalysts are classified into two categories: homogeneous and heterogeneous catalysts.^21,22^ Molecular catalysts, as an example of homogeneous catalysts, have a defined structure, tunability, high atom economy, and a defined catalytic approach.^23,24^ However, their practical applicability is marred by poor stability arising from rapid structural degradation under oxidative conditions, thus impeding their widespread adoption.^25^ On the other hand, heterogeneous catalysts based on transition-metal oxides, hydroxides, oxyhydroxides, and their derivatives present unique benefits, such as low cost, high activity, and remarkable long-term stability in alkaline solutions.^26–29^ Thus, it has been widely studied into single/poly crystalline pure metal electrocatalysts for water splitting, focusing more on enriching characteristics as the key factor, such as selectivity and activity for OER and HER.

Traditional metal electrodes cover a wide range of materials, including noble metals (e.g., gold, silver, platinum), transition metals (e.g., iron, cadmium, zinc), and main group elements (thallium, indium, tin, cadmium, lead). Copper stands out among these as a leading candidate due to its electronic properties that allow for the selective formation of multicarbon products, with Faradaic efficiencies remarkably high; therefore, copper becomes an ideal ground for probing possible reaction pathways and optimizing operating parameters to improve sustainably advantageous conversions.^30,31^ However, the practical applications of these technologies are limited due to the high costs and limited supply of some metals.^32^ Consequently, substantial research endeavors are currently underway aiming to identify alternative catalysts that demonstrate improved water-splitting activity, including noble metals, carbon-based materials, and abundant metal alloys and oxides.^33,34^ Attaining the pinnacle of catalytic performance and stability demands meticulous structural and electronic design refinement, particularly for OER, involving gas–liquid–solid phase interactions, is critically influenced by the ability of reactants and intermediates to effectively adsorb and desorb on the catalyst surface.^35,36^

Superior OER and HER catalysts should exhibit the following key characteristics: (1) exceptional electrical conductivity to augment electron transfer efficiency and reduce intrinsic resistance; (2) high density of readily accessible catalytic active sites; (3) enhanced intrinsic activity of these active sites; (4) robust mechanical and chemical stability; (5) catalyst surfaces with superhydrophilic and superoleophobic properties, promoting the adsorption of water molecules and the desorption of oxygen; and (6) scalable, environmentally sustainable synthesis methodologies. Consequently, a myriad of strategies have been explored to augment electrical conductivity and optimize performance in the context of OER and HER, such as manipulation of the microstructure and morphology of oxide materials to amplify surface area and facilitate the exposure of additional active sites^37,38^ and incorporation of heteroatoms into composites to improve charge transfer and introduce additional active sites.^39,40^ In electrocatalysis, the active reaction can be influenced by three essential characteristics: the number of active sites (which relates to surface area and porosity), intrinsic activity, and the surface charge transfer kinetics.^41^ The tuning of these parameters is key to markedly improving electrocatalytic activity. The intentional synthesis of metal-based nanostructures possessing well-defined morphologies, controllable sizes, and tunable surface areas make them exceptional catalysts for numerous reactions. Among this group, multimetal hydroxides have superior performance due to their adjustable metal-cation ratios, electronic structures, interface properties, and chemical stability.^42^ Although slight modifications of morphology, the presence of low levels of dopant ions, and the careful structure design of heterojunctions are used, the structure–activity relationship remains to be fully explored.^43^ Additionally, these catalysts offer considerable compositional versatility, increasing electrocatalytic activity via synergistic effects that fine-tune the adsorption energies of intermediates and the surface electronic structure for precise control of water splitting and specific product formation.

Trimetallic nanostructures (TMNs) make use of three different metals and have attracted considerable interest due to their potential for outstanding applications in HER,^44^ OER,^45^ oxygen reduction reaction (ORR),^46^ alcohol oxidation reaction (CO_2_RR),^14^ and formic acid oxidation.^47^ TMNs serve as remarkable materials in providing distinctive catalytic activity along with enhanced stability, often avowing performance that surpasses monometallic and bimetallic counterparts. In a nutshell, their unusual configurations arise from pure alloying or clustering of three differing metals, providing very specific tuning in each composition’s ratios, size, and morphology. These modifications have opened other avenues of opportunity for addictive optimization, facilitating promising electrocatalytic performance across several reactions.^48^ The systematic design of trimetallics, based on their use of Earth-abundant elements, with controlled applied parameters like surface area and elemental composition, among other factors, is also raising their catalytic efficiency and versatility to a focal point in advanced nanomaterial applications.^49^ For example, Qin et al. synthesized trimetallic phosphorus–selenium composites on carbon cloth (NiV_2_P, FeSe@CC), a result of new OER parameters, accomplishing a serious feat of 168 mV at 10 mA cm^–2^ with a low Tafel slope of 44.32 mV dec^–1^.^45^

Trimetallic nanostructures have become very attractive electrocatalysts because of their synergistic effects and special benefits in water splitting, especially those based on first-row transition metals like Fe, Co, and Ni. By optimizing the binding energies of reaction intermediates, as required by Sabatier’s principle, the addition of three different metal components allows for the fine-tuning of electronic structures, which in turn increases catalytic activity. Reaction kinetics are enhanced by higher active site density of these nanostructures, which is made possible by their high surface areas and customized morphologies. Furthermore, the synergistic interactions of metal centers enhance charge transfer efficiency and electron delocalization, lowering overpotentials for both HER and OER. The adaptability of trimetallic systems also makes them more stable in alkaline environments, reducing deterioration over extended use. Trimetallic nanostructures overcome the drawbacks of monometallic and bimetallic catalysts by utilizing their synergistic qualities to provide an affordable and scalable alternative for successful hydrogen production. Therefore, considering the uptick in research interest and the bright prospects related to the catalytic properties of TMNs, it is, therefore, high time and imperative to contribute with an elaborate overview of the recent advances on their synthesis strategies and catalytic applications. It is surprising that out of all reviews carried out addressing health, environment, and general in nature, very few address the gallant electrocatalysts for water splitting.^40,47^ Accordingly, although several reviews have given emphasis to their stability as trio-based materials for energy conversion and arguably a given perspective addressing the divergent systems, synthesis journeys, structural attributes, and promises is available, the review recap on most recent research evidence is certainly evincible. In this review, we will present a concise yet comprehensive overview of the latest advances in trimetallic-based catalysts, their design or fabrication, characterization, and catalytic mechanisms concerning water splitting. To this end, the review will start with the fundamentals of water splitting, OER and HER. Then we will perform a comprehensive review of the different types of nanostructured trimetallic materials for OER, HER, and water splitting including metal organic frameworks (MOFs), layered double hydroxides (LDHs), metal oxides, spinels, phosphides, nitrides, chalcogenides, alloys, and composites. Synthesis methods, structural characterization, theoretical calculations, catalytic mechanisms concerning water splitting, and advantages of trimetallic catalysts over bimetallic benchmarks are also highlighted. Finally, the manuscript concludes with a discussion of challenges and future perspectives for scalable electrocatalytic applications.

## 2 Fundamentals of Water Splitting

The general efficiency of water splitting plays a vital role in the possibility of large-scale applications; thus, this requires complete understanding of the reaction mechanisms and other important steps that influence the rate and potential. Such understanding, however, serves to optimize the development of electrocatalysts and improve electrolyzer design to minimize energy losses of conversion. Therefore, in this case, this section provides a brief yet complete description of some well-known reaction mechanisms and concepts that help to facilitate a deeper understanding of the complexity of the water splitting reaction.

The conventional water electrolyzer comprises an anode, a cathode, and an electrolytic solution. In electrochemical water splitting, this involves two key half reactions: (1) proton reduction, a process at the cathode commonly called the HER; (2) water oxidation, the OER taking place at the anode. According to the Nernst equation under standard conditions (25 °C, 1 atm), the standard reduction potential is +1.23 V vs RHE for OER and 0 V vs RHE for HER. Thus, from a thermodynamical viewpoint, a potential of 1.23 V vs RHE is required for the overall process of water splitting in any electrolyte, a nonspontaneous process that can be driven by electrolysis.^50^ This requirement is due to the inherent stability of the water molecule that requires a net energy input of 237.2 kJ mol^–1^ at standard conditions (i.e., 25 °C, 1 atm) for dissociation into hydrogen and oxygen.^51^ Although the mechanism depends on the electrolytic solution, the overall equation of the water splitting process can be represented by several steps, as shown in Figure 1.

**Figure 1:**
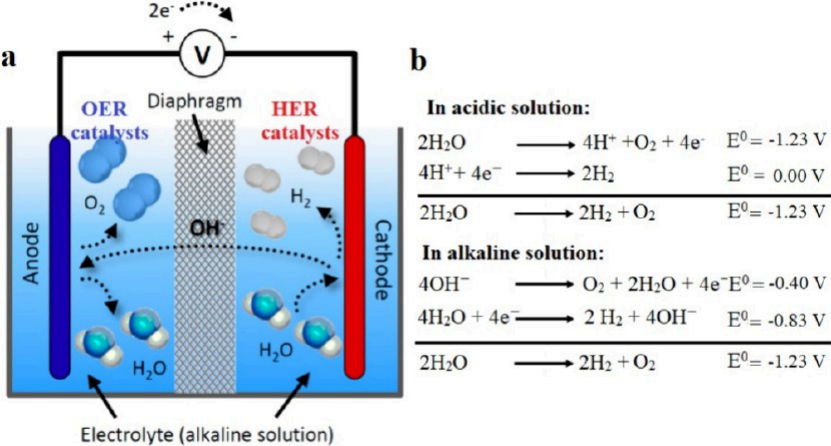
(a) Schematic representation of a conventional water electrolyzer. (b) Water splitting reactions under acidic and alkaline conditions.

Copyright 2021, Elsevier.^52^

In practical applications, water-splitting is hindered by high activation energy barriers, slow reaction kinetics, and low energy efficiency. Overcoming these barriers requires utilizing a potential greater than the theoretical 1.23 V vs RHE established from the Nernst equation.^53^ Consequently, the reaction will need to be driven quasi-equilibrially along its pathway under more kinetically favorable conditions by the application of an added potential, called the overall operational potential (OP).

$$E_{op}=1.23V+\eta_{a}+\left|\eta_{c}\right|+iR$$ 1

In the context of water splitting, *i*R means the ohmic potential drop arising from ionic electrolyte resistance; it thus has scope for some optimization in an electrolyzer setup. At the anode and cathode, just like with any other electrochemical system, η_a_ and η_c_ characterize the overpotential in overcoming the processes driven by activation energies. If highly efficient HER and OER catalysts are used, it can be expected that those activation barriers, η_a_ and η_c_, will become lower. Yet still, the resistance and activation energy of the electrode materials, electrolyte diffusion rates, dynamics of bubbles, and heat dissipation can give rise to impressive variations of the overpotentials. For this reason, it has become very important to develop effective electrocatalysts using low-cost and abundant materials to improve the overall performance in water electrolyzers. Following this, a detailed description of both anodic and cathodic reactions is presented.

### 2.1 Hydrogen Evolution Reaction (HER)

In acidic solutions, HER kinetics is largely dictated by hydrogen recombination processes. In contrast, alkaline HER requires a synergistic interplay between water dissociation and the dynamics of surface-adsorbed hydrogen intermediates.^54^ Here, the reaction proceeds through three key elementary steps at the catalyst’s active sites (A) (Figure 2A): in the acidic media, the HER initiates with the Volmer step (1), where the interaction between an electron (e^–^) and a proton (H^+^) at the electrode interface leads to the formation of a chemisorbed hydrogen species (H_a__d__s_) on the surface, facilitated by the high proton availability. In other words, the Volmer step is responsible for capturing and anchoring a proton to the electrode surface. Subsequently, the hydrogen desorption process can take one of two routes: electrochemical desorption via the Heyrovsky steps or chemical desorption via the Tafel step. The Heyrovsky step (2) involves the reaction of H_a__d__s_ with another proton and electron, leading to the formation and desorption of molecular hydrogen (H_2_) from the electrode surface. Alternatively, the Tafel step (3) entails the recombination of two H_ads_ species to generate H_2_, which then desorbs from the surface. The HER pathway may proceed via the Heyrovsky mechanism, the Tafel mechanism, or a combination of both, as described in eqs 2–5).^55,56^



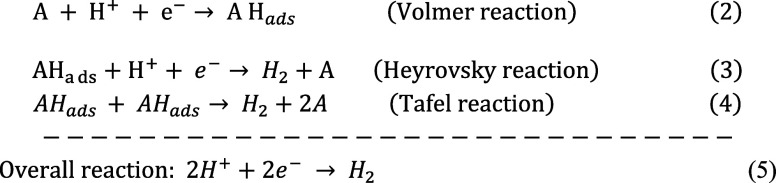

2



**Figure 2:**
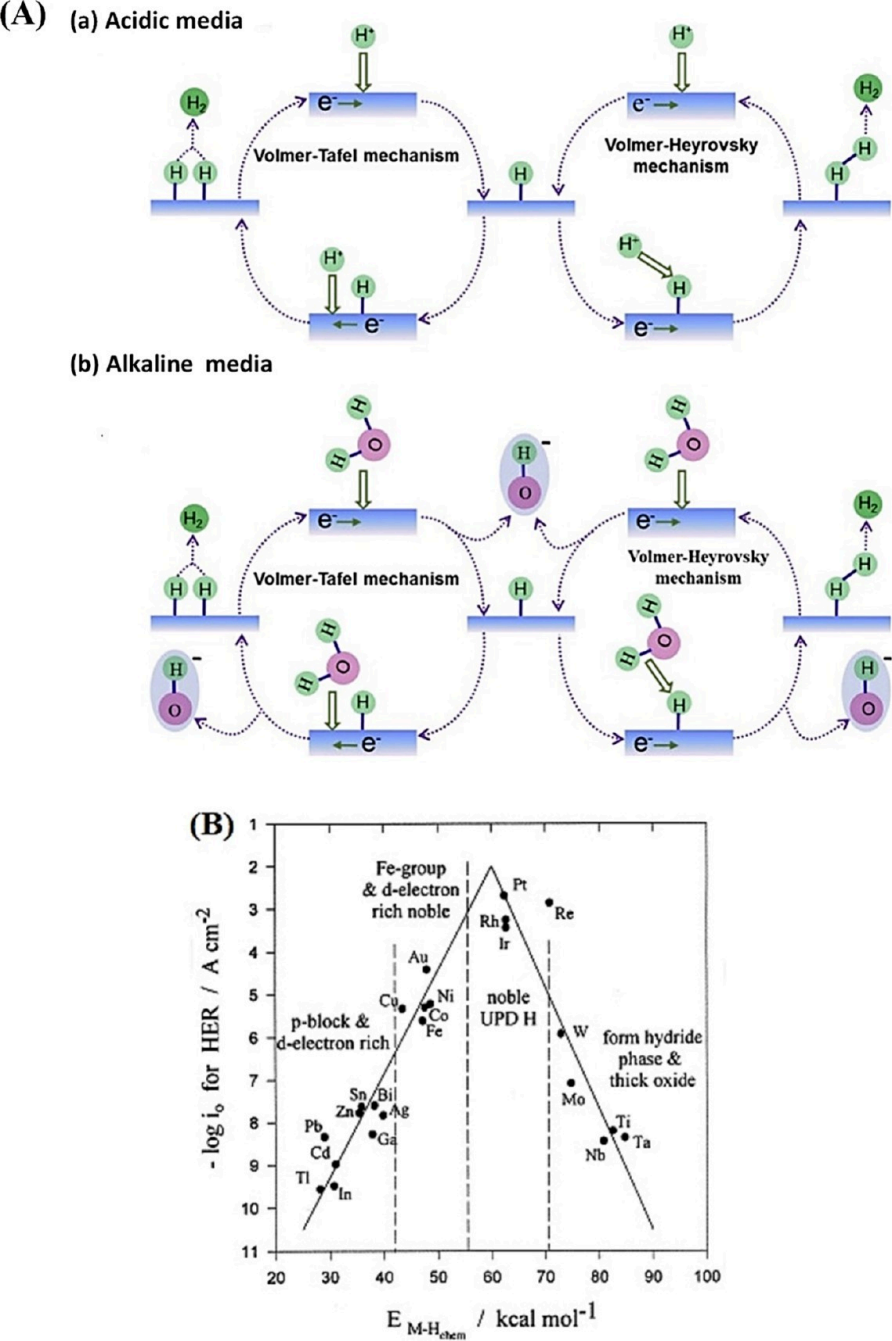
(A) HER mechanism in acidic and alkaline media. Adapted with permission from ref 66. Reprinted with permission, (B) Volcano curve for electrocatalysis of the HER at various pure metals in terms of dependence of log i_o_ values on metal-to-H bond energy.

Copyright 2020, MDPI Inc.

Copyright 1972, Elsevier.^62^

In alkaline media, the concentration of protons decreases, and as a result, the HER mechanism differs from that under acidic conditions. In the Volmer and Heyrovsky steps, water dissociation serves as the initiating reaction. The Tafel step, on the other hand, is similar to as it is in acidic conditions (eq 6–9).^57^


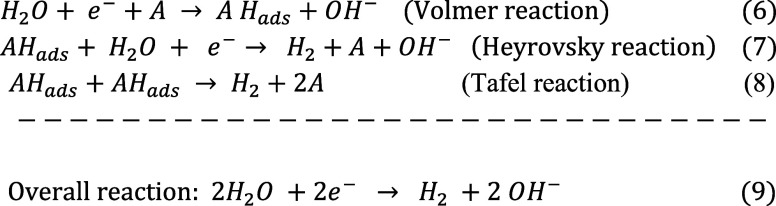

6



The Tafel slope obtained from linear sweep voltammetry (LSV) is crucial for understanding the extent of HER in these active sites. Generally, the Tafel slope represents the transfer of electrons via the interface of the electrode and electrolyte, and further, the slopes can indicate what might be the rate-determining step (RDS): ∼29 mV dec^–1^ for the Tafel step, ∼40 mV dec^–1^ for the Heyrovsky step, and ∼120 mV dec^–1^ for the Volmer step. Notably, in alkaline media, usually the RDS is the Volmer step, while this role might be reverse in acidic conditions, with the Heyrovsky step being the RDS. A lower Tafel slope shows improved kinetics, which allows for high current densities with low overpotentials.^58^

HER is strongly reliant on hydrogen adsorption free energy (Δ*G*_H*_) that directly determines the strength of hydrogen binding to the surface of the catalyst. In accordance with the Sabatier principle, a perfect electrocatalyst would ensure an optimal value of Δ*G*_H*_ (around 0 eV), which would allow for efficient proton–electron transfer of the surface reaction and, in addition, would easily allow H_2_ to desorb from the surface.^59–61^ Parsons’ volcano plot (Figure 2B) features the Δ*G*_H*_ vs *j*_0_ relationship, where Δ*G*_H*_ values closer to zero imply higher catalytic activity.^62^ Positive Δ*G*_H*_ values indicate weak adsorption of hydrogen with a limiting effect on activation, and negative values indicate strong adsorption and site blocking and poisoning of the catalyst.^63–65^ Δ*G*_H*_ is thus a determining parameter for HER performance.

In the context of trimetallic catalysts, determining active sites remains contentious, especially in alkaline environments where water molecules are polarized to release H^+^ ions. Enhanced HER activity is linked to metals that effectively polarize water, facilitating H_2_ production via surface M–H bonds. Conversely, within an acidic medium, the determination of active centers is contingent upon the materials employed and their hydrogen-binding affinity. In this context, mechanistic steps exhibit variation, exerting a discernible influence on the rate of hydrogen evolution.

### 2.2 Oxygen Evolution Reaction (OER)

It has been reported in various studies that the OER mechanism is centered on a sequence of the electrochemical adsorption and desorption of oxygen-based compounds on the active sites of the catalyst. A major complication for the OER process is the larger number of intermediates and steps involved for the transfer of the electrons along with proton transfer. Among other aspects, the most important are the four forms of intermediates (*OH, *O, *OOH, and *O_2_) and the slow kinetics due to the O=O bond formation occurring only at high overpotentials are the event response elements.^67^ Furthermore, OER manifests distinct overall reactions under acidic and alkaline conditions (Figure 3A and B), which has prompted the proposal of a myriad of reaction mechanisms for the OER at an acidic medium (eqs 10–15). In contrast to HER, the OER demands a significantly greater thermodynamic potential.^68,69^ This elevated potential increases energy costs, limiting water splitting efficiency for large-scale hydrogen production. Furthermore, it is widely acknowledged that the formation of oxygen typically occurs on metal oxide or oxyhydroxide surfaces or metal-based compounds like phosphides or sulfides rather than on bare metal surfaces.^70^ This is because metal oxides/oxyhydroxides provide more favorable active sites and surface chemistry for the formation of O_2_ molecules during the reaction.^68,69^ In practical terms, irrespective of the electrode type employed, electrocatalysts tend to undergo a preoxidation process, resulting in the formation of an in situ grown oxide or oxyhydroxide thin layer, with metal cations assuming higher oxidation states.^71^ The redox potential and electronic structure of these cations critically influence the activation energy and reaction kinetics.^72^ Similar to the HER, the OER pathways are pH-dependent, with electron transfer processes varying in acidic and alkaline conditions, as illustrated by the following eqs for alkaline medium (eqs 16–20).^73^

**Figure 3:**
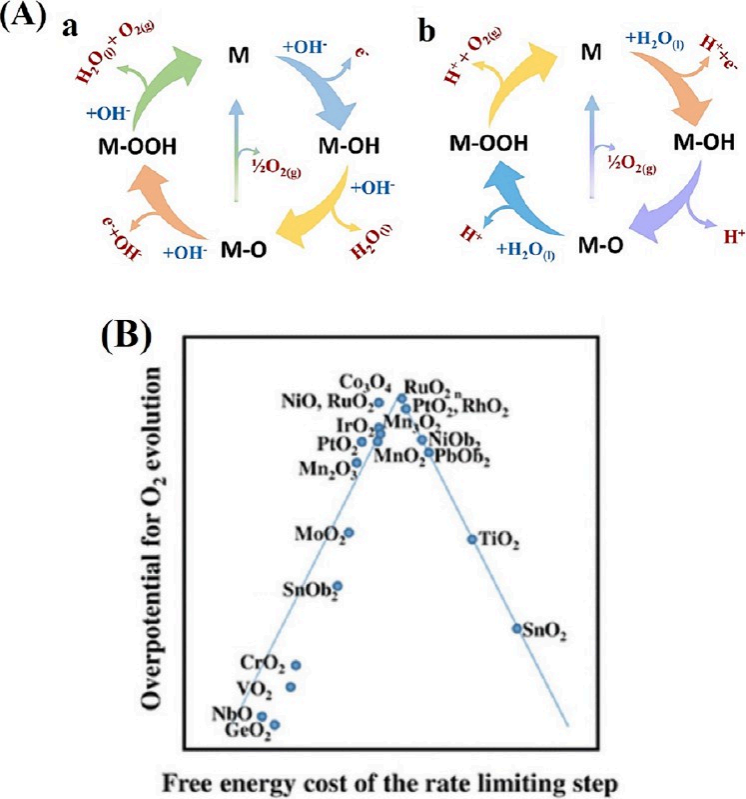
(A) OER mechanism in alkaline (a) and in acidic media (b; Reprinted with permission). (B) The OER volcano plot for metal oxides. Reprinted with permission.

Copyright 2021, Wiley Inc.^76^

Copyright 2011, Wiley.^75^

In acidic medium:

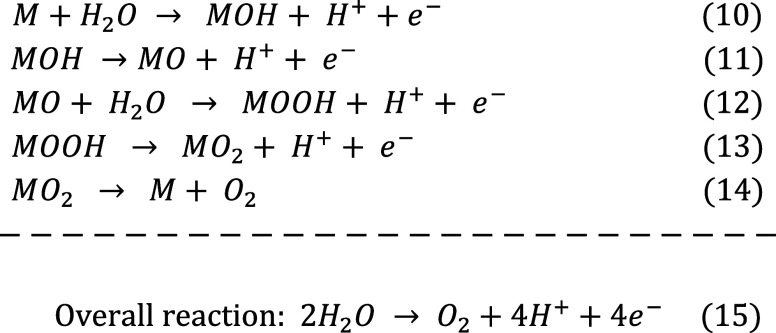

10



In alkaline medium:

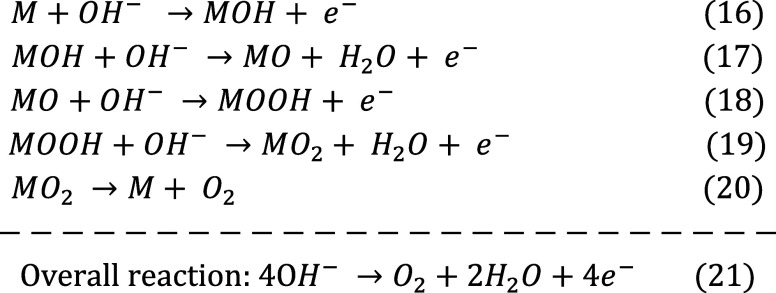

16



Noble and seminoble metal-based catalysts (e.g., Ir, Ru, and their oxides) exhibit superior OER activity in acidic media, while other transition metal-based electrocatalysts perform more effectively in alkaline conditions. The OER mechanism involves the adsorption of H_2_O or OH^–^ at metal (M) active sites, forming *OH intermediates via single-electron oxidation. Further oxidation yields *O, followed by *OOH intermediate formation through additional H_2_O or OH^–^ adsorption. However, the formation of *OOH is thermodynamically challenging due to its weak binding to the metal surface, which results in a positive free energy change. To reduce the overpotential for the OER, it is essential for one or more metal components to be oxidized to higher oxidation states. The cleavage of the metal–oxygen (M–O) bond is critical for regenerating the active metal site, necessitating an optimal bond strength that aligns with the Sabatier principle to achieve efficient catalysis.

Theoretical insights into the OER process identify the binding energies of HO*, O*, and HOO* intermediates on oxide surfaces as key descriptors of catalytic activity. Both excessively strong and weak oxygen binding hinder performance.^74^ Man et al. correlated the activity descriptor (Δ*G*_O_ – Δ*G*_OH_) with experimental overpotentials of various metal oxides, leading to the development of volcano plots that elucidate the overpotential origins and guide catalyst optimization.^75^ Revised volcano plots (Figure 3B) using the same descriptor reveal that no materials currently occupy the peak, indicating significant potential for enhancing OER activity by optimizing the binding strength between the catalyst surface and reaction intermediates.

## 3 Advancements in Trimetallic and Derived Materials for OER

### 3.1 Key Considerations in Electrocatalyst Development

OER typically follows the adsorbate evolution mechanism (AEM), involving successive proton–electron transfer steps on metallic sites through various oxygenated intermediates. However, the scaling relationship between the Gibbs free energy of *OOH and *OH imposes a theoretical overpotential limit of 0.37 V at a current density of 10 mA cm^–2^ (η_10_) for even the most efficient catalysts.^77^ A recent shift toward the lattice oxygen oxidation mechanism (LOM) has introduced an alternative approach that overcomes traditional O–O bond formation constraints, improving catalytic efficiency.^78^

For optimal industrial water electrolysis, key factors include ample active sites, efficient bubble diffusion, and strong mechanical and chemical stability. Non-noble transition metal-based electrocatalysts offer a cost-effective and abundant solution, enabling photo-assisted OER with high efficiency. But the OER electrocatalysts still have stability issues and slow reaction kinetics under acidic conditions due to the sluggish electron and proton transfer. Selecting stable, active catalysts for acidic media is crucial. Enhancing efficiency involves minimizing overpotential, reducing side reactions, ensuring stability, and maximizing catalytic activity.

Groundbreaking advances such as perfecting the porosity of the material and increasing the surface area promote the interaction of the electrode and the electrolyte and create a larger exposure of the active sites. While Ir and Ru oxides are highly efficient OER catalysts, their scarcity and poor durability limit practical use. Therefore, the development of non-noble metal-based electrocatalysts, in particular, first-row transition metals (e.g., Fe, Co, Ni) that achieve high oxidation states, is vital for the efficient OER in water splitting (Table 1).^79,80^ Optimizing catalytic activity in oxides based on transition metals is very important. Including heteroatom ligands (N, P, S) increases cooperation among metal ions and therefore improves OER activity. These ligands, with multiple chelation sites, coordinate with multiple metal ions, forming cross-linked networks that facilitate electron transfer and organized structures. Secondary stage dopants infer more change in the electronic and chemical environment of active sites, therefore enhancing OER efficiency and catalytic performance.^81^ Analyzing the binding energy of Ni^2^^+^ and Fe^2^^+^ shows that POx doping enhances charge transfer rates and redistributes the electronic structure in electrocatalysts. To overcome metal oxide resistance, carbon-matrix materials can improve conductivity and efficiency.

**Table 1:** OER Performance of Trimetallic Electrocatalysts in Alkaline Electrolyte (1 mol L^–1^ KOH)

catalysts	substrate	(mA cm^–2^)	η (mV)	Tafel slope (mV dec ^–1^)	ref
NiV_2_P/FeSe	CC	10	168	44.32	^45^
FeCoNi-PA-300	NF	50, 100	271, 286	52	^82^
Ni(OH)_2_/NiFe LDHs	NF	10	220	48.5	^83^
Cu/NiFe LDH	NF	100	259	36.35	^84^
CuCoZn–S-3	CF^a^	10, 100	175, 242	62.3	^68^
FeCoNiOxHy	NF	200	257	2	^11^
FeCoNi-PBA	NF	10	236	43.8	^85^
Co_3_O_4_@CoNi-LDH	NF	50	360	73.5	^86^
Co_3_O_4_@NiFe-LDH/NF-100	NF	50	270	34.59	^87^
Ru-NiFe LDH/NiCo_2_O_4_	CC	10	225	78	^88^
NiCo_2_S_4_@NiFe LDH	RDE^b^	10	287	86.4	^89^
Ni–Mo–S@NiFeLDH	NF	100	274		^90^
Co_9_S_8_@NiFe LDH	GCE	10	220	52	^91^
(Fe, Ni, Co)_9_S_8_@CS	GCE	10	260	46.50, 50	^92^
FeCoNiOx/C	NF	10	221	21	^93^
PdFeCo_3_-x O_4_	NF	100	300, 340	52	^94^
MoCoFe-HO@CoMo-LDH	GCE	10	324	45.11	^95^
Pd-ZIF-8/ZIF-67	CC	10	340	85	^96^
CoNiMn-MOF	NF	20	220	66	^97^
CoFeMo-A (A = P, Se)	GCE	10	273, 299	31.2, 39.5	^98^
Co_2_FeO_4_ @ PdO	GCE	20	259	59	^99^
Cu_0.19_Mo_0.19_/Co_0.62_O NPs@RGO	GCE	10	250	61	^100^

a Copper foam.

b Rotating disk electrode.

### 3.2 Advances in Transition Metal-Based Electrocatalysts

Carbon matrices offer high conductivity, surface area, and stability, preventing agglomeration and enhancing OER performance. Heteroatom (e.g., N, P, S) doping in carbon matrices adjusts electron density, enhancing conductivity and catalytic activity. Heteroatom-doped carbon networks produce efficient electron transfer routes and more reactive sites, thereby improving general catalytic activity and long-term stability. For example, Zhang et al.^101^ reported that V-doping improves the conductivity and reduces OER overpotential. Powdered electrocatalysts suffer from low conductivity, detachment, and poor electron/mass transfer, therefore limiting their industrial applicability.^102^

Free-standing electrocatalysts with active components on conducting porous substrates including carbon cloth are important to tackle these difficulties. By virtue of being very flexible, highly conductive, and low-cost, carbon cloth is preferred for sophisticated catalytic applications.^103,104^ TMPs on carbon cloth are good OER electrocatalysts since they are more damage resistant under alkaline circumstances than metal oxides/hydroxides but unstable under oxidation. With the TMP core producing conductivity, their active sites develop as surface metal oxides/hydroxides. On carbon cloth, Qin et al.^45^ designed a Ni–Fe–V trimetallic phosphorus–selenium composite (NiV_2_P/FeSe@CC) with high conductivity, a multilevel pore structure, and efficient charge/mass transport to improve OER performance.

Electrochemical deposition enables uniform Ni and Fe deposition on V-metal organic frameworks (V-MOF), while P and Se codoping modulates the catalyst’s electronic structure, enhancing OER activity by promoting intermediate adsorption (Figure 4A). Anion incorporation, particularly Se with higher electronegativity than P, optimizes TMPs’ electronic configuration, improving electron density and kinetics for superior OER performance.^105^ The carbon cloth substrate, with its 3D porous structure, supports uniformly distributed cuboid MOF crystals (300–350 nm). Fine particles uniformly cover the V-MOF surface following electroplating, as shown in Figure 4B. Their successful incorporation of Ni and Fe into the VMOF@CC system is further proved by the carbon fiber diameter increases. After phosphorus-selenization, SEM analysis of NiV_2_P/FeSe@CC reveals a porous honeycomb morphology, enhancing gas transport and catalytic site utilization during OER. The electrocatalyst achieves a low overpotential of 168 mV at 10 mA cm^–2^ and a Tafel slope of 44.32 mV dec^–1^ in alkaline conditions. In an integrated electrolyzer, it maintains excellent performance with a voltage of 1.53 V at 10 mA cm^2^ and stability over 160 h at 100 mA cm^2^ (Figure 4C). In comparison to Se-NiFeV@CC (62.47 mV dec^–1^), P-NiFeV@CC (89.03 mV dec^–1^), PSe-NiFe@CC (92.36 mV dec^–1^), NiFeV@CC (95.68 mV dec^–1^), and NiFe@CC (97.8 mV dec^–1^), the Tafel plot in Figure 4C shows that NiV_2_P/FeSe@CC has the lowest slope (44.32 mV dec^–1^). Since NiV_2_P/FeSe@CC has a smaller Nyquist semicircle diameter, EIS measurements further support a lower charge transfer resistance (8.1 Ω), indicating enhanced interfacial charge transfer during OER. This superior performance is attributed to the multilevel pore structure of the 3D carbon cloth-supported honeycomb composite, enhancing the electrocatalytic activity of the freestanding catalyst. He et al. utilized phytic acid (PA) as a carbon matrix precursor, forming a cross-linked network with transition metal ions (Fe, Co, Ni) through phosphate complexation.^82^ PA complexation forms a cross-linked network, enhancing metal site synergy. Shifting from metal phytates to phosphate species regenerates carbon structures and creates a porous morphology by decomposing PA. This reorganization helps to expand the electrochemically active surface area by means of improved metal ion distribution and conductivity. Phosphate ligands raise catalytic activity even more by changing electron density around transition metals and carbonaceous rings as well as chemical environment. At modest overpotentials of 271 mV and 286 mV, respectively, the FeCoNi-PA-300 catalyst outperforms FeCoNi-300, attaining present densities of 100 and 50 mA cm^–2^. Moreover, the nonlocal density functional theory (NLDFT) is used to estimate the pore size distributions (PSDs) for FeCoNi-300 and FeCoNi-PA-300. Because of the separation of big phosphate groups from carboatomic rings, FeCoNi-PA-300 has more mesopores than FeCoNi-300, despite both being mesoporous materials. Phosphorus-containing groups have the potential to introduce a multimodal pore size distribution with peaks in both the microporous and mesoporous/microporous regions. In order to enable ion transport and increase OER activity, the hierarchical porous structure made up of micropores, mesopores, and macropores can efficiently increase the permeability of the electrolyte to the internal surface and offer short, low-resistance ion diffusion channels

**Figure 4:**
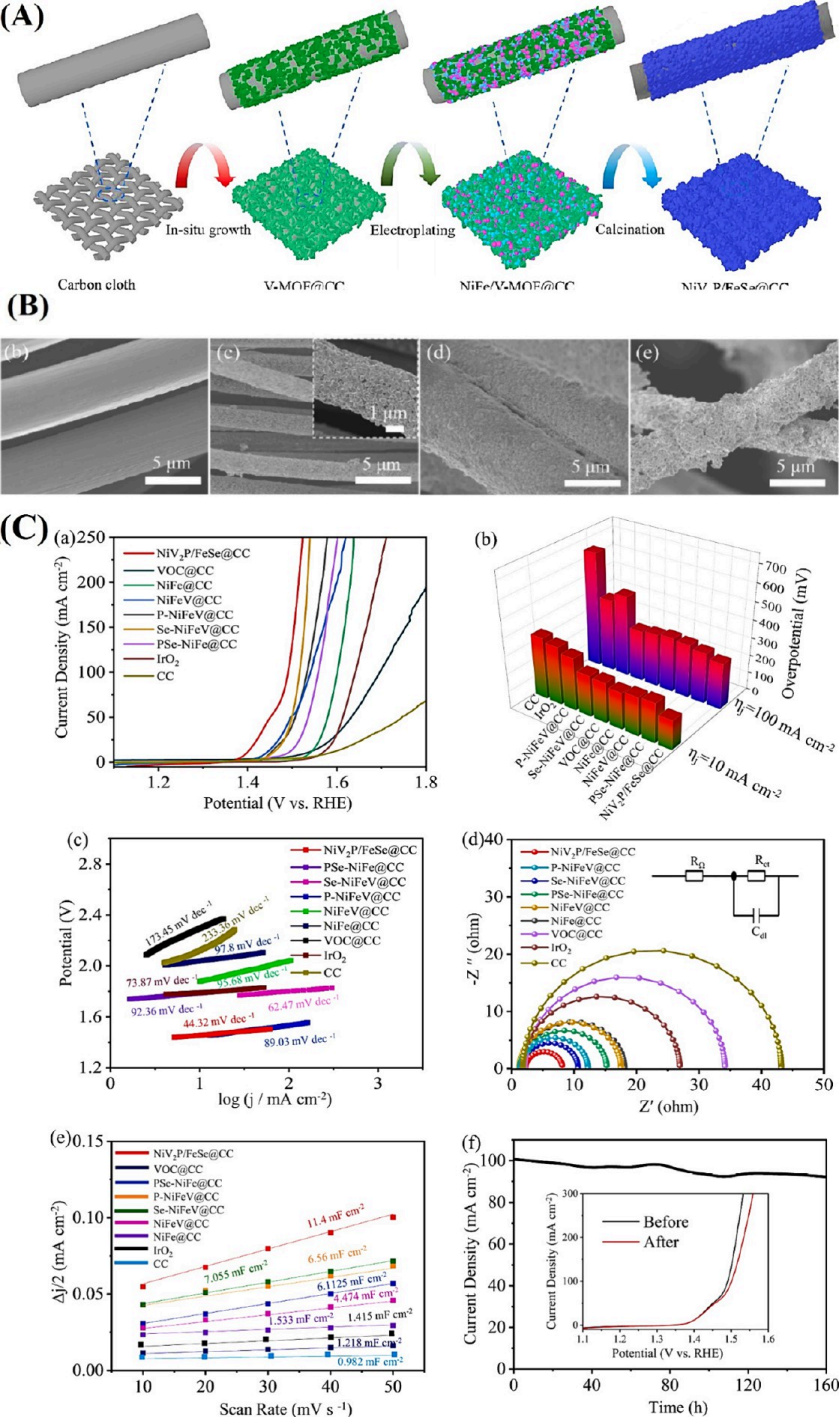
(A) Scheme showing the fabrication of NiV_2_P/FeSe@CC. (B) SEM images of (b) carbon cloth, (c) V-MOF@CC, (d) NiFe/V-MOF@CC, and (e) NiV_2_P/FeSe@CC. (C) (a) LSV plots under 5 mV s^–1^, (b) overpotentials, (c) Tafel plots, (d) Nyquist curves, and (e) *C*_dl_ of different electrocatalysts. (f) Chronopotentiometry profiles of NiV_2_P/FeSe@CC under 100 mA cm^–2^ (inset: polarization curves before and after stability testing).

Copyright 2024, Elsevier.^45^

Among various LDH systems, NiFe LDH nanosheets are a leading option for electrocatalytic OER because of their anionic clay makeup, hydrotalcite-like structure, and many active sites. In alkaline settings, they beat other LDH systems including NiMn, NiCo, and CoFe. In bimetallic LDHs including iron, such as NiFe, the inclusion of iron allows the formation of oxygen-bridged Fe–M centers that stabilize the *O intermediate and lower the overpotentials at 10 mA cm^–2^ to 348 mV for NiFe, in comparison to CoFe with 404 mV. At the same current density, the partnership of iron and nickel in sites like (Fe, Ni) OOH encourages the creation of O and OOH species, therefore attaining overpotentials of 300 to 400 mV. Coordinating tuning, structural adjustments, and vacancy engineering all together can lower overpotentials to 230–270 mV.^106^ On the other hand, the impact of fostering cooperation among various metal ions in this OER catalyst may extend beyond just nickel and iron. According to a recent study, the catalytic activity could be further enhanced by adding a third metal, such as Co, Cr, Mn, or Mo.^107^ Better inherent electrical conductivity of these multielement electrocatalysts also boosts charge transfer efficiency, hence OER kinetics and total catalytic performance. Greater intrinsic conductivity maximizes catalytic site use and deals with thickness-dependent constraints on OER activity. Using traditional approaches based on conductive support, integration of Fe with Ni and Co oxyhydroxides activates the Fe enters, therefore lowering overpotentials and boosting catalytic activity greatly. These interactions improve charge transport efficiency. Moreover, improving both conductivity and catalytic activity helps Fe, Co, and Ni-based electrocatalysts to have greater OER efficiency. Iron helps to slow the oxidation of Co and Ni, therefore guaranteeing formation of conductive oxyhydroxides that maximize the inherent catalytic ability of Fe. Leading nonprecious metal OER electrocatalysts, NiFe-LDHs have stacked-sheet design, cationic defects, and outstanding electrical qualities. Their Fe–Ni synergy enhances performance, offering superior stability and durability in alkaline conditions compared to RuO_2_ and IrO_2_.^108^ A favorable OER process is produced by the introduction of a third metal and its beneficial influence on the local electronic structure, even though the identity of the catalytic active sites is still unknown.

The fabrication of porous and hierarchical-structured trimetallic NiFeX-LDHs (where X represents a third metal such as Co, Cu, or Mn) significantly enhances surface area, active site exposure, electrolyte diffusion, and gas bubble release, leading to superior catalytic performance for water splitting. The incorporation of a third metal into the NiFe-LDH framework introduces synergistic electronic interactions that optimize active site reactivity and stability compared to bimetallic NiFe-LDHs.

Implementing heterojunctions with elements like nitrogen, phosphorus, and sulfur between NiFeX-LDH and semiconductors reduces aggregation, further enhances active site exposure, and improves electrocatalytic performance by modulating bandgaps for optimized light absorption.^109^

Integrating these trimetallic LDHs with 3D conductive frameworks (e.g., nickel foam, copper foam, carbon fiber paper) enhances conductivity and eliminates the need for insulating polymeric binders, ensuring efficient electron transport during OER. Recent studies reveal that adding Ni­(OH)_2_ layers into the NiFeX-LDH interlayer improves OER activity and durability with the oxidation of Ni­(OH)_2_ to NiOOH serving as a primary catalytic site, facilitating a faster transformation sequence compared to Ni-based catalysts (Ni → NiO → Ni­(OH)_2_ → NiOOH). However, NiFeX-LDHs remain susceptible to dissolution under harsh conditions. While adding secondary materials to generate heterogeneous composites enhances structural integrity, incorporating such materials into the LDH interlayers remains challenging due to the complex interplay of the trimetallic components. The Ni­(OH)_2_/NiFe LDHs exhibit a distinct multilayer microstructure that differs from the monolayer of NiFe LDHs, as seen in Figure 5A, suggesting that interlayers were implanted into the NiFe LDHs. To verify the effect of the introduction of Ni­(OH)_2_ on OER performance, the self-supported electrodes Ni­(OH)_2_/NiFe LDHs/NF were directly employed as working electrodes to determine their OER electrocatalytic activities. LSV curves show that OER performance varies with Ni­(OH)_2_ content, with Ni­(OH)_2_/NiFe LDHs-0.1 (0.1 Ni­(OH)_2_) exhibiting the best activity, achieving the lowest overpotential of 220 mV at 10 mA cm^–2^, outperforming NiFe LDHs (258 mV), Ni­(OH)_2_ (280 mV), NF (342 mV), and RuO_2_ (290 mV). This enhancement is attributed to the synergistic interaction between NiFe LDHs and Ni­(OH)_2_ layers. Additionally, Ni­(OH)_2_/NiFe LDHs-0.1 shows the lowest Tafel slope (48.45 mV dec^–1^) and the highest specific activity (90 mA cm^–2^ at 1.6 V RHE). EIS and electrochemical surface area (ECSA) analyses reveal that Ni­(OH)_2_/NiFe LDHs-0.1 has the highest double-layer capacitance (*C*_dl_ = 34.5 cm^2^) and lowest charge transfer resistance (*R*_ct_ = 0.74 Ω), indicating improved active sites and faster charge transfer, contributing to its superior OER performance. These findings underscore the pivotal role of Ni­(OH)_2_ in governing the electronic and microstructural properties, optimizing OER kinetics, and enhancing both activity and stability in NiFeX-LDHs (Figure 5B).^110^

**Figure 5:**
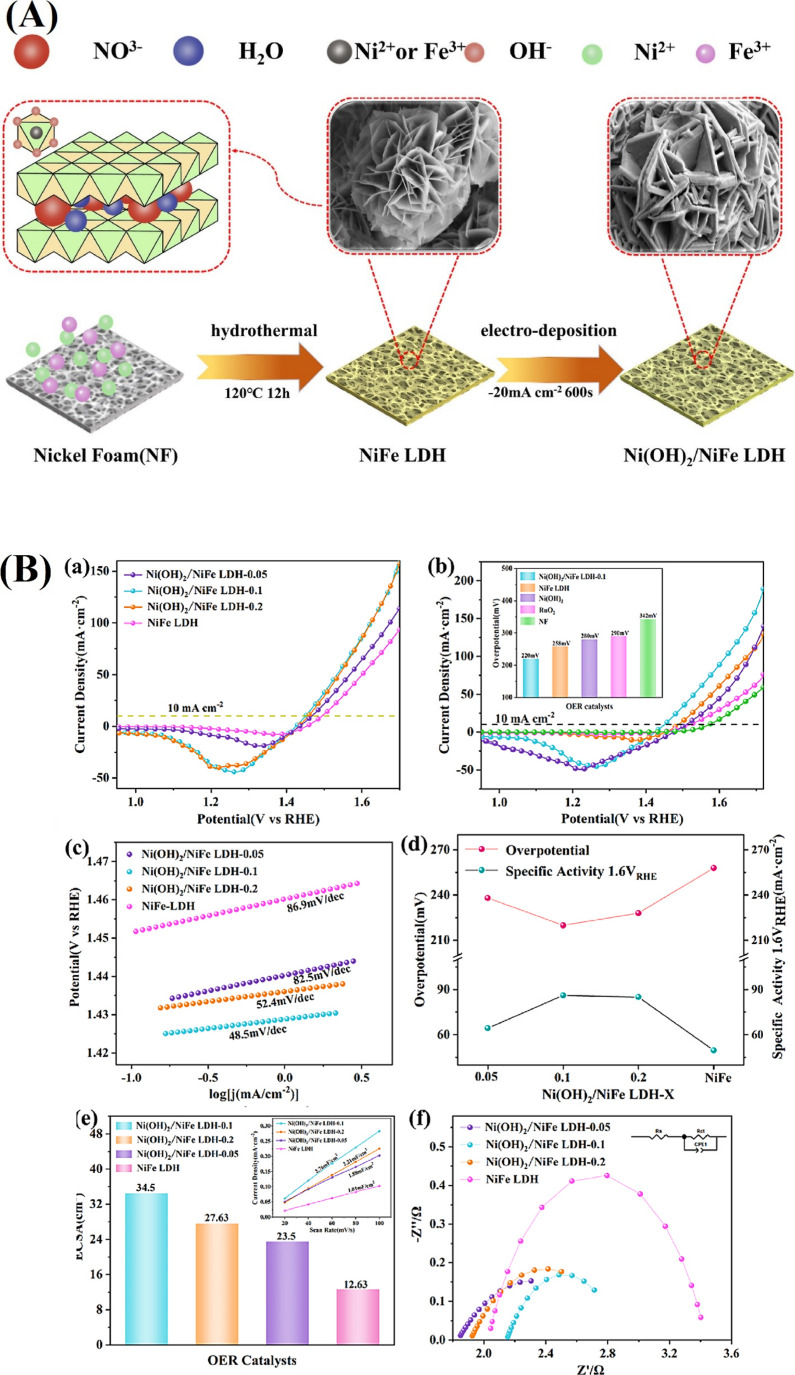
(A) Schematic illustration of the synthetic route for Ni­(OH)_2_/NiFe LDHs. (B) Comparison of LSV curves of (a) Ni­(OH)_2_/NiFe LDHs-x and (b) Ni­(OH)_2_, NiFe LDHs, Ni­(OH)_2_/NiFe LDHs-0.1, RuO_2_, and NF. (c)­Tafel slope of Ni­(OH)_2_/NiFe LDHs-x. (d) Overpotential and specific activity. (e) ECSA, inset is the linear relationship between Δ*j* and the different scan rates. (f) EIS spectra and the inset is the equivalent circuit.

Copyright 2023, Elsevier Inc.^110^

Introduction of the third metals (e.g., Co, Cr, Mn) advances oxidation state adjustment and adds yet another step toward enhanced catalysis by accelerating electron interactions.^111^ As with the mechanism at large, identifying the very specific nature of OER catalytic active sites still evades research, though insertion of a third metal species with effects upon localized electronic structure is found to instigate optimal OER kinetics.

The Cu/NiFe-LDH nanosheets, pieced together by the entrapment of copper nanoclusters into the nickel–iron LDH matrix, improve the metal site complex via carefully tuned contact with surface oxygen moieties, enabling rapid electron transport and improved active-site availability and durability, via bonded electron transfer and structural protection from the LDH.^84^ Additionally, the charge density contrast simulation findings showed that electrons moved from Cu to the coupled interface between NiFe LDH and Cu, where oxygen gained electrons and the hydrogen at the outermost layer of LDH lost electrons. In testing, Cu/NiFe-LDH-2.5 demonstrated exceptional OER performance with a low overpotential of 259 mV at 100 mA·cm^–2^ and a Tafel slope of 36.35 mV·dec^–1^, showcasing the potential of heteroatom modifications in enhancing electrocatalyst efficiency. Employing heteroatoms to modify the electronic structure of metallic compounds represents a viable strategy for enhancing the intrinsic activity of electrocatalysts. Conversely, due to the limited electrical conductivity inherent in LDHs, carbon-based materials, including graphene oxide (GO) and carbon nanotubes, doped with heteroatoms such as B, N, S, and P, have emerged as electrochemically active alternatives. Nguyen et al. developed a trimetallic nitrogen-doped CoNiFe catalyst to improve OER efficiency. The enhanced performance results from (1) synergistic interactions among metal sites, (2) uniform active site distribution, (3) LDH’s ability to intercalate water and anions, enhancing redox capabilities, and (4) nitrogen doping in the CoNiFe matrix, which optimizes conductivity and OER intermediate adsorption.^112^

Introducing heteroatoms near transition metals in trimetallic catalysts enhances catalytic efficiency by allowing precise control over structural configurations and morphology, which significantly impacts reaction outcomes. Transition metal sulfides often face issues like self-agglomeration, limited intrinsic conductivity, and insufficient active sites, but integrating them with conductive substrates can improve architecture, conductivity, and active site exposure, enhancing electrochemical performance.^113^ The CuCoZn–S-3 catalyst, shaped like a nanoflower, exhibits a high specific surface area that enhances active site exposure and charge transfer kinetics. Its flower-like configuration produces interfacial gaps that help OER kinetics as well as enhance electrolyte–catalyst interactions. The combined interactions of Cu, Co, and Zn help to maximize redox pathways, therefore improving electrochemical performance. The surface Cu ions in the copper foam (CF) were replaced with Cu­(OH)_2_, followed by hydrothermal addition of Co and Zn to form CuCoZn–OH precursors with an acupuncture-like structure in fabricating CuCoZn–S-3@CF. The three-dimensional nanoflower architecture of nanorod and leaf-like structures resulted from a 3 h sulfidation treatment (Figure 6A). Additionally, the catalyst’s shape changed slightly as a result of controlling the curing duration. CuCoZn–S-1 first developed a flower-like structure after 1 h of curing, which grew from rods to a single flower-like structure. The CuCoZn–S-6 catalysts’ morphology changed when the curing time was extended to 6 h. The rods came together to form a large blade, which was subsequently stacked to create a new flower-like structure. Stated otherwise, the catalyst’s nanostructure will accumulate as a result of the sulfurization process, changing its shape. CuCoZn–OH assumed a flaky nanoflower morphology, as revealed in the SEM images, which changed in CuCoZn–S-3 to a stereoscopic nanoflower (Figure 6B). The CuCoZn–S-3 catalyst has the highest electrocatalytic activity, as seen in Figure 6C. It requires only 175 mV of overpotential to reach a current density of about 10 mA cm^–2^, while Cu­(OH)_2_ requires 322 mV. It also achieves a high current density of 100 mA cm^–2^ with a lowest overpotential of 242 mV, as opposed to 446 mV for Cu­(OH)_2_. This indicates that sulfurization, which creates ordered nanostructures and increases active sites on rough surfaces, significantly enhances electrocatalysis. Furthermore, CuCoZn–S-3 performs better than CuCo-S and CuZn-S, indicating that the three metals have stronger synergistic interactions.^68^

**Figure 6:**
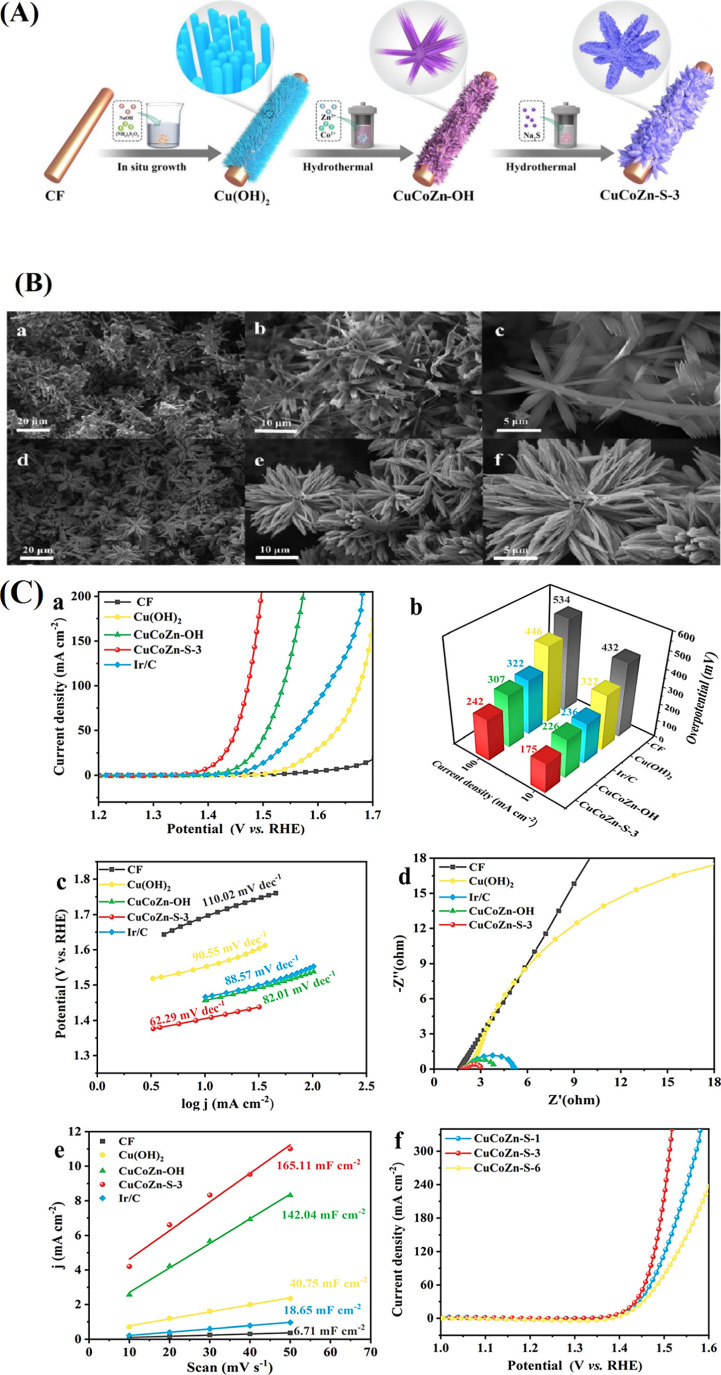
(A) Schematic diagram of the CuCoZn–S nanoflower catalyst supported on CF. (B) SEM images of as synthesized (a–c) CuCoZn–OH and (d–f) CuCoZn–S-3. (C) OER performance test of electrocatalysts under alkaline conditions. (a) LSV polarization curves, (b) overpotential at 10 and 100 mA cm^–2^, (c) corresponding Tafel plots, and (d) Nyquist plots. (e) *C*_dl_ values and (f) LSV polarization curves at different times.

Copyright 2022, RSC.^68^

The FeCoNiOxHy material takes advantage of the electronic properties of Fe, Co, and Ni hydroxides to improve OER performance. This synergy lowers the Gibbs free energy for *OOH intermediate formation, a key OER step, and improves conductivity by promoting Co^3+^ generation. The combined OER activity of Fe^3+^ and the conductivity of Co^3+^ increase OER kinetics and produce a small overpotential of 257 mV at 200 mA cm^–2^ in 1 mol L^–1^ KOH.^11^ Furthermore, FeCoNiOxHy’s abundant ultrasmall crystalline grains contained in its huge amorphous region should produce a broad, hierarchical porosity that enhances mass transfer and active site exposure during OER. As the associated anodic peak charge rises from 3.1 to 8.8 and then 58.0 mC, the OER overpotential falls from 363 mV for NiOxHy to 295 mV for CoOxHy and then to 268 mV for CoNiOxHy. As the equivalent OER overpotential drops, it goes from 555 mV for FeOxHy to 292 mV for FeNiOxHy, 268 mV for FeCoOxHy, and eventually to 227 mV for FeCoNiOxHy. On the other hand, the DFT data showed that the electron density around Co in bimetallic CoNiOxHy and trimetallic FeCoNiOxHy clearly decreases when compared to monometallic CoOxHy, while the electron density around Fe and Ni in FeCoNiOxHy clearly increases when compared to FeNiOxHy because of the electron transfer from Co to Fe and Ni.

M-site transition metal ions are tethered on a (Prussian blue analogues) PBA scaffold in the synthesis of trimetallic FeCoNi-PBA materials, resulting in metal hydroxides that upon electrochemical oxidation convert into metal (oxy)­hydroxides and release metal cyano groups ([M’(CN)_6_]­y^–^).^85^ This reorganization boosts active site reactivity and accessibility for OER. Under anodic potential, the PBA-based structure converts into carbon-encapsulated metal (oxy)­hydroxides, optimizing catalytic center exposure. The conductive, hydrophilic carbon matrix enhances electron transfer and electrolyte diffusion, yielding a minimal overpotential of 236 mV at 10 mA cm^–2^, a modest Tafel slope of 43.8 mV dec^–1^, and long-term stability under current densities of 100 and 400 mA cm^–2^. Integrating transition metal oxides with double-layer hydroxides offers a promising route for efficient, nonprecious metal OER catalysts. Co_3_O_4_ spinel catalysts attract interest for OER due to the interplay of Co^2+^ and Co^3+^, but their low conductivity limits performance.^114,115^ Introducing heterojunction interfaces, such as with NiFe-LDHs, can enhance conductivity and catalytic activity by redistributing charge toward Fermi-level alignment and optimizing *OOH adsorption. Despite progress, the long-term effects of heterojunctions on metal–water interactions and stability across current densities remain an ongoing research challenge.^86^ The combination of NiFe-LDH nanoparticles with Co_3_O_4_ creates diverse interfaces that improve surface area and electron density distribution. As shown in Figure 7A, on NF, the Co_3_O_4_@NiFe-LDH/NF fabrication process included several complex stages. The fabrication entails thermally oxidizing nickel foam to produce Co_3_O_4_, which then acts as a substrate for in situ growth of NiFe-LDH. The fabrication involves synthesizing Co­(OH)_2_ on nickel foam, which is thermally oxidized to form Co_3_O_4_, serving as a substrate for in situ growth of NiFe-LDH (Figure 7B). The Co_3_O_4_ nanowires acted as a core during this stage, offering nucleation sites for the adsorption of Ni^2+^ and Fe^3+^ and therefore helping in situ creation of the outer shell including NiFe-LDH particles. Core–shell Co_3_O_4_@NiFe-LDH allows better accessibility of the electrolyte to the interface, and charge transfer helps to increase OER efficacy. NiFe-LDH nanoparticle growth started by Co_3_O_4_ nanowires acting as a seed; 3D architecture enhances electron transport, and electrolyte access arose. This structure, with unsaturated Co sites and a crystalline/amorphous NiFe-LDH phase, achieved a low overpotential of 270 mV at 50 mA cm^–2^ and excellent stability (Figure 7C).^87^ For comparison, the Co_3_O_4_@NiFe-LDH/NF-100 electrode achieves an overpotential of merely 270 mV at 50 mA cm^–2^ (η_50_), substantially lower than Co_3_O_4_@NiFe-LDH/NF-50 (η_50_ = 285 mV), Co_3_O_4_@NiFe-LDH/NF-200 (η_50_ = 306 mV), and Co_3_O_4_/NF (η_50_ = 393 mV). The optimal performance of Co_3_O_4_@NiFe-LDH with a Ni/Fe ratio of 1:1 arises because a too low NiFe-LDH loading decreases the amount of active sites, while excessive loading hinders electron transport from Co_3_O_4_ to NiFe-LDH. Additionally, the EIS data show that Co_3_O_4_@NiFe-LDH/NF-100 has the lowest *R*_ct_ (0.10 Ω) compared to Co_3_O_4_/NF (0.32 Ω), Co_3_O_4_@NiFe-LDH/NF-50 (0.25 Ω), and Co_3_O_4_@NiFe-LDH/NF-200 (0.27 Ω), indicating enhanced charge transfer. The cointroduction of Ni and Fe boosts activity, with core–shell heterostructures via electrodeposition significantly improving Co_3_O_4_/NF performance. Finally, Co_3_O_4_ and NiFe-LDH can create a special electron channel of Co–Ni–Fe during OER, lowering the reaction barrier to increase catalytic activity, as shown by microstructure analysis and the density of states (DOS) computation.

**Figure 7:**
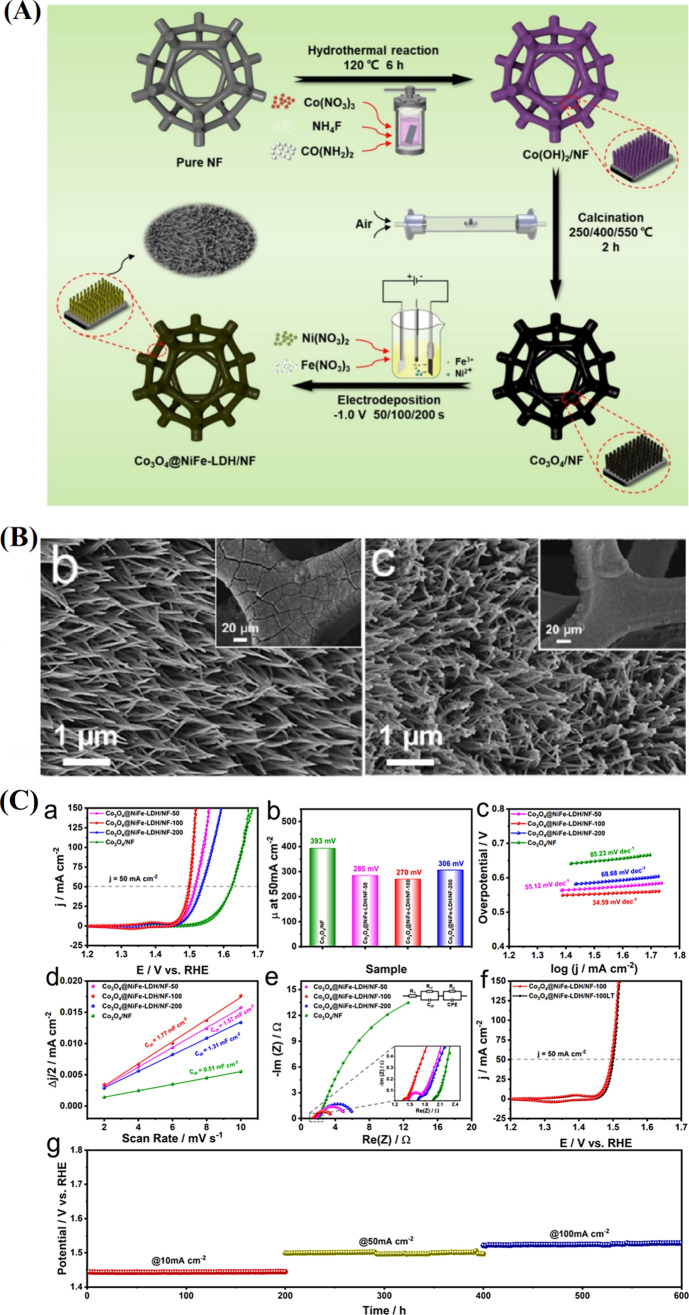
(A) Schematic illustration for the synthesis process of the Co_3_O_4_@NiFe-LDH/NF hybrid material. (B) SEM images of (b) Co_3_O_4_/NF and (c) Co_3_O_4_@NiFe-LDH/NF-100. (C) (a) CVs of Co_3_O_4_@NiFe-LDH/NF-50, Co_3_O_4_@NiFe-LDH/NF-100, Co_3_O_4_@NiFe-LDH/NF-200, and Co_3_O_4_/NF. (b) Histogram of each sample’s overpotential at 50 mA cm^–2^. (c) Tafel slopes. (d) Plot of scan rates versus capacitor current at 0.0 V (vs Hg/HgO). (e) Nyquist graphs at 1.50 V (vs RHE). (f) CVs of Co_3_O_4_@NiFe/NF-100 and Co_3_O_4_@NiFe/NF-100LT. (g) *V*–*t* plots of Co_3_O_4_@NiFe-LDH/NF-100 at 10, 50, and 100 mA cm^–2^ for 200 h.

Copyright 2024, Elsevier.^87^

Similarly, Co_3_O_4_@CoNi-LDH nanoarrays on nickel foam, synthesized via MOF templating and atomic layer deposition, improve surface reactivity and electron transport. This binder-free 3D catalyst showed enhanced OER performance with lower overpotential and higher current density than IrO_2_.^86^ Surface reactivity and electron mobility were higher in the CoNi-LDH layer. Dao et al. engineered a novel composite catalyst with ruthenium-doped NiFe-LDH nanosheets enriched with defects, encapsulating NiCo_2_O_4_ nanowires (NCO NWs) on a carbon cloth (CC) scaffold.^88^ The core–shell configuration enhanced the surface area, edge exposure, and thus electrocatalytic performance (Figure 8A). SEM observation confirms the vertical uniform growth of NCO NWs on CC, with Ru SA-doped NiFeAl-LDH in parallel neatly covering the nanowires (Figure 8B). Single-atom (SA) doping increases electrocatalytic activity but presents difficulties including dissolution in electrolytes during OER owing to high-valence state transition.^116^ In electrocatalysis, integration of vacancies and defects in the materials enhances catalytic activity as well as SA structure stabilization. In terms of material modification, this strategy enhances the electronic structure and coordination environment that the materials are more accessible to withstand high-valence transitions (e.g., OER).^117^ Defects also reveal more unsaturated sites, which become active sites for water reduction and oxidation. This also raises metal–oxygen bonding, reduces lattice distortions, and hence promotes long-term stability and catalytic activity.^118^ Hence, by adopting a core–shell architecture incorporating NCO and def-Ru-NiFe LDH, the surface area can be maximized, edges can be exposed, and the electrolysis performance of def-Ru-NiFe-LDH nanosheets can be significantly enhanced. As illustrated in Figure 8C, def-Ru-NiFe LDH/NCO performs better than other materials and IrO_2_, requiring only 225 mV overpotential for 10 mA cm^–2^, compared to 250 mV (Ru-NiFeAl LDH/NCO), 275 mV (NiFe LDH/NCO), 340 mV (NCO), and 375 mV (IrO_2_). Its superior activity stems from strong Ru single atom interactions with defect-rich NiFe LDH, enhanced by atomic cation vacancy defects that activate new catalytic sites. According to the DFT analysis, strong interactions between Ru atoms and defects in NiFe LDH reduced the OER overpotential (340 mV at the Ru site).

**Figure 8:**
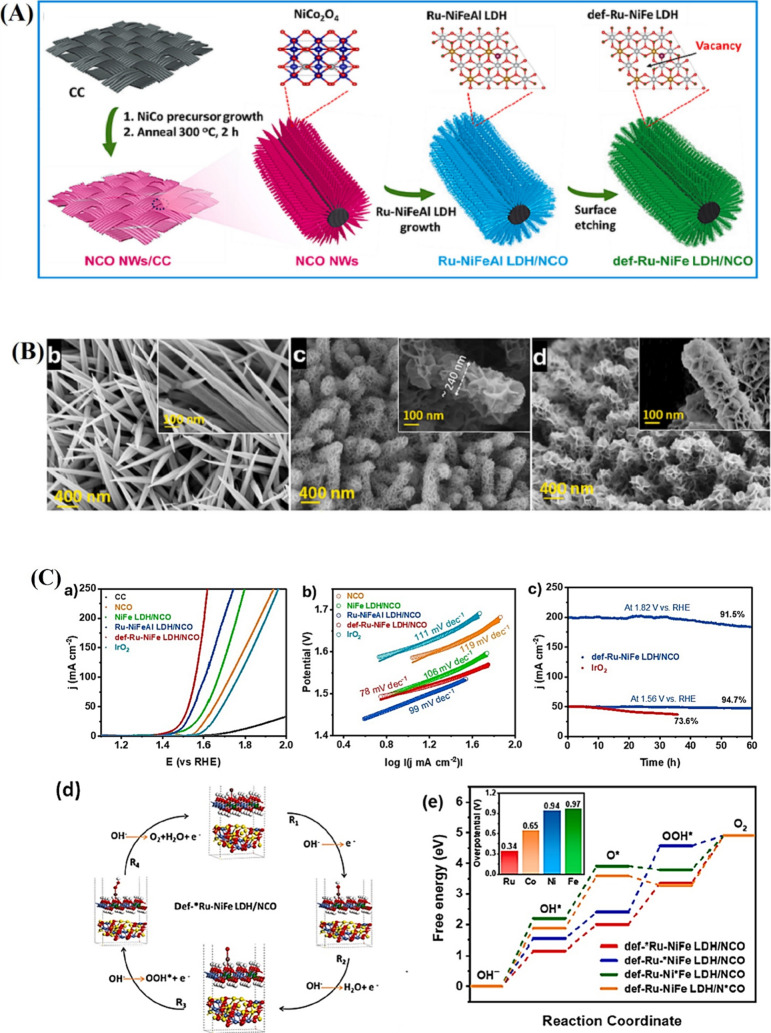
(A) Schematic illustration of the synthetic procedure of def-Ru-NiFe LDH/NCO. (B) (b–d) FE-SEM images of NCO, Ru-NiFeAl LDH/NCO, and def-Ru-NiFe LDH/NCO. (C) (a) 90% *iR*-corrected OER polarization curves of the fabricated samples and IrO_2_. (b) Tafel plots of the fabricated samples for OER. (c) Chronoamperometric test of def-Ru-NiFe LDH/NCO and IrO_2_ for long-term OER operation at low and high applied voltages. (d) Proposed mechanism for OER on the Ru site of def-Ru-NiFe LDH/NCO. (e) Gibbs free energy changes for OER pathways involving Ru, Ni, Fe, and Co active sites of def-Ru-NiFe LDH/NCO.

Copyright 2024, Elsevier.^88^

### 3.3 Transition Metal Chalcogenides and Nonoxides

In addition to their environmental friendliness, structural versatility, low cost, and high conductivity, transition metal sulfides (TMSs) are especially attractive for electrocatalysis. Materials like M_*x*_S_γ_ (M = Fe, Co, Ni, etc.) show potential for OER, with multimetal systems enhancing d-orbital interactions and optimizing intermediate adsorption/desorption. Ternary metal sulfides, in particular, offer superior catalytic performance but face challenges due to complex chemical bonding and phase heterogeneity during the synthesis.^119^ Recent advances in structure-tailored synthesis aim to overcome these challenges, exploring novel configurations beyond traditional pyrite-type (ABS_2_) and spinel-type (AB_2_S_4_) structures.^120^ Some of the bimetallic TMSs include NiCo_2_S_4_, which are promising for ORR because of different valence principles or crystalline structures, but their OER activities are limited by sulfur leaching, crystallographic decomposition at oxidative states, and structural alterations.^89^ On the contrary, the most noticeable OER catalytic performers are NiFe-LDH, which have very good stability, and can be integrated in. The heterostructured composite retains the intrinsic characteristic of the building blocks and exhibits novel or enhanced characteristics that are a product of synergistic effects offered by the heterointerface. Additionally, enhanced bifunctional catalysis is made possible in heterostructured composites, such as Ni–Mo–S core layers encapsulated by NiFe-LDH layers. For example, a Ni–Mo–S/NiFe-LDH core–shell structure on nickel foam demonstrated exceptional OER and urea oxidation reaction (UOR) performance, highlighting the benefits of heterointerface design in improving catalytic activity.^90^

Recent studies suggest that cobalt-based sulfides are high-quality substitutes for noble metals in OER industries, and cubic Co_9_S_8_ is considered as a suitable catalyst in this regard because of its outstanding electrocatalytic properties and exceptional redox ability. However, traditional TMSs often degrade into metals or (oxy)­hydroxides during OER, losing sulfur and compromising their stability and efficiency. In contrast, NiFe-LDHs have gained attention for their adaptable composition, resistance to degradation, and superior catalytic performance. Hybridization of NiFe-LDH with conductive materials enhances interfacial electronic properties and OER activity. Combining NiFe-LDH with conductive materials like Co_9_S_8_ enhances interfacial electronic properties, improving OER activity. Nanostructuring enhances electrochemical performance to a further level. In addition, these hierarchical hollow structures with hierarchical surface areas and hydrophilic properties facilitate diffusion of the reactants and detachment of the bubbles, which promote the OER process. For example, in the urchin-like Co_9_S_8_@NiFe-LDH heterostructured hollow spheres shown in Figure 9A, the hydrophilic Co_9_S_8_ hollow spheres allowed for uniform Fe^3^^+^, Ni^2^^+^, and urea coating, forming porous NiFe-LDH nanowires in situ. The Co_9_S_8_ hollow spheres are coated with NiFe-LDH nanowires, functioning as the skeletal nucleation sites for Ni^2+^ and Fe^3+^ ion adsorption and formation in situ of the NiFe LDH nanowires. The pure NiFe LDH nanowires are randomly coupled and tend to congregate, unlike the widely dispersed urchin-like Co_9_S_8_@NiFe LDH microspheres. This core–shell structure enhances liquid transport, structural integrity, and catalytic efficiency by exposing active sites and optimizing electron transfer. This design feature provides a high specific surface area (SSA) and exposed reactive sites for Co_9_S_8_@NiFe LDH, which exhibits high OER activity, requiring an overpotential of only 220 mV at 10 mA cm^–2^, outperforming Co_9_S_8_ (282 mV), NiFe LDH (342 mV), and commercial IrO_2_ (341 mV). Its Tafel slope of 52.0 mV dec^–1^ is lower than Co_9_S_8_ (57.7 mV dec^–1^), NiFe LDH (89.0 mV dec^–1^), and IrO_2_ (91.6 mV dec^–1^), indicating favorable OER kinetics. Additionally, its lower *R*_ct_ value (5.87 Ω) compared to Co_9_S_8_ (7.24 Ω) and NiFe LDH (9.96 Ω) reflects enhanced charge transfer ability (Figure 9D). According to DFT calculations, the synergistic interaction of Co_9_S_8_ and NiFe LDH at heterointerfaces significantly alters the RDS and lowers their Gibbs free energy, which in turn increases OER catalytic activity (Figure 9C).^91^ Hierarchical hollow structures enhance catalysis with abundant active sites, fast ion/electron transport, and improved structural stability during OER.

**Figure 9:**
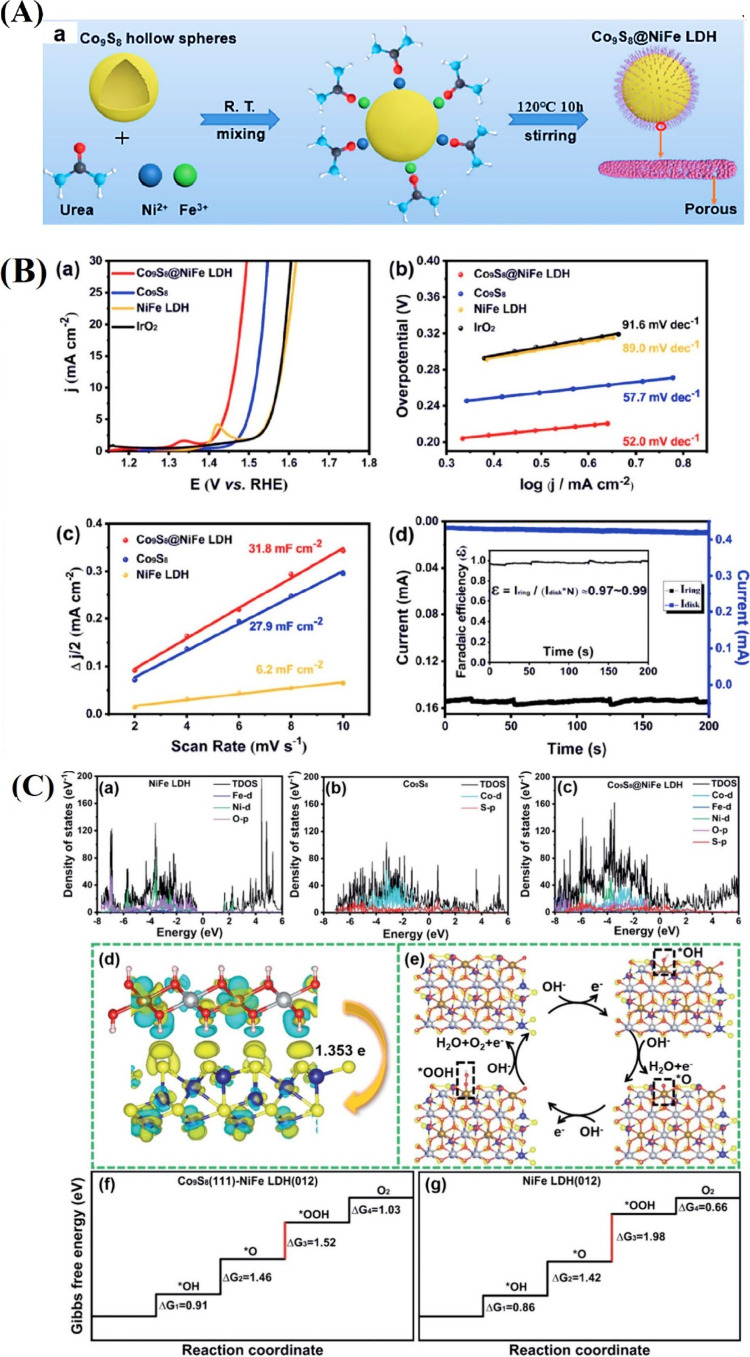
(A) (a) Schematic diagram for the synthetic process of the Co_9_S_8_@NiFe LDH. (B) (a) OER polarization curves and (b) Tafel plots of the Co_9_S_8_@NiFe LDH, Co_9_S_8_, NiFe LDH, and IrO_2_. (c) The plots of half of the current density variation (*j*_anodic_ – *j*_cathodic_) at 1.29 V vs scan rate for the Co_9_S_8_@NiFe LDH, Co_9_S_8_, and NiFe LDH. (d) Disk and ring currents of Co_9_S_8_@NiFe LDH (inset: faradaic efficiency of Co_9_S_8_@NiFe LDH). (C) The density of states (DOS) of (a) NiFe LDH, (b) Co_9_S_8_, and (c) Co_9_S_8_@NiFe LDH. (d) Charge density difference at the Co_9_S_8_@NiFe LDH interface with an isosurface level of 0.002 e bohr, and yellow and light blue represent the charge accumulation and depletion regions, respectively. Schematic illustration of the OER pathway at the (e) Co_9_S_8_@NiFe LDH interface and (f) on NiFe LDH. The free energy diagram of the OER processes at 0 V at the (g) Co_9_S_8_@NiFe LDH interface and (h) on NiFe LDH.

Copyright 2021, RSC.^91^

Transition metal chalcogenides (TMChs) are becoming next-generation bifunctional catalysts for OER and HER, with tuning of the catalytic activity via doping, shape control, and heterostructure.^121^ Despite their potential, the lower conductivity compared to noble metals necessitates surface engineering, such as nanostructuring the catalysts themselves to enhance surface area and defect density, as well as anchoring them onto highly conductive substrates to facilitate rapid charge transfer.^122^ Pentlandites (TM_9_Ch_8_), a subgroup of TMChs, exhibit high catalytic efficiency and can be used in bulk forms, simplifying synthesis while maintaining performance. The crystal structure of cobalt pentlandite (Co, Ni, Fe)_9_S_8_ is characterized by having a wide range of composition in the Fe–Ni–Co-S system, with a cubic crystal structure, and crystallizes in the space group *Fmm* with a unit cell with 36 metal cations distributed among 4 octahedral sites, 4b sites, and 32 tetrahedral sites, 32­(f) in this space group. The 32 S anion atoms in the cobalt pentlandite structure are accommodated in two distinct sites, with 8 atoms occupying the 8c site and the remaining 24 atoms located in the 24e site. Metal atoms bind these anions in 4- and 5-fold coordination geometries. Such structural plasticity implies that conducting constituents of the material may be tunable.^123^ Incorporation of heavy metals such as palladium, silver, rhodium, and ruthenium into the lattice of cobalt pentlandite provides a versatile composition that allows for the precise tuning of active sites for enhanced catalytic performance.^92^ In a recent study, Kim et al. synthesized a cobalt pentlandite structure, (Co,Ni,Fe)_9_S_8_, as an active OER catalyst via controlled synthesis.^92^ The controlled atomic arrangement in the crystalline structure enables precise tuning of electronic properties, enhancing performance. The researchers combined ball milling with pyrolysis, successfully facilitating pentlandite synthesis and carbon encapsulation. Carbon encapsulation was provided by the aniline monomer, and sulfur for sulfide formation was supplied by ammonium persulfate. By adjusting the Ni/Co ratio, three compounds (FeNiCoS-21, FeNiCoS-11, FeNiCoS-12) were synthesized (Figure 10A). SEM images revealed spherical structures on a carbon network, highlighting their morphology (Figure 10B). The unique crystal structure and nanoflower morphology enabled synergistic metal interactions, achieving a low overpotential of 260 mV at 10 mA cm^–2^ in alkaline conditions, showcasing enhanced catalytic stability (Figure 10C).

**Figure 10:**
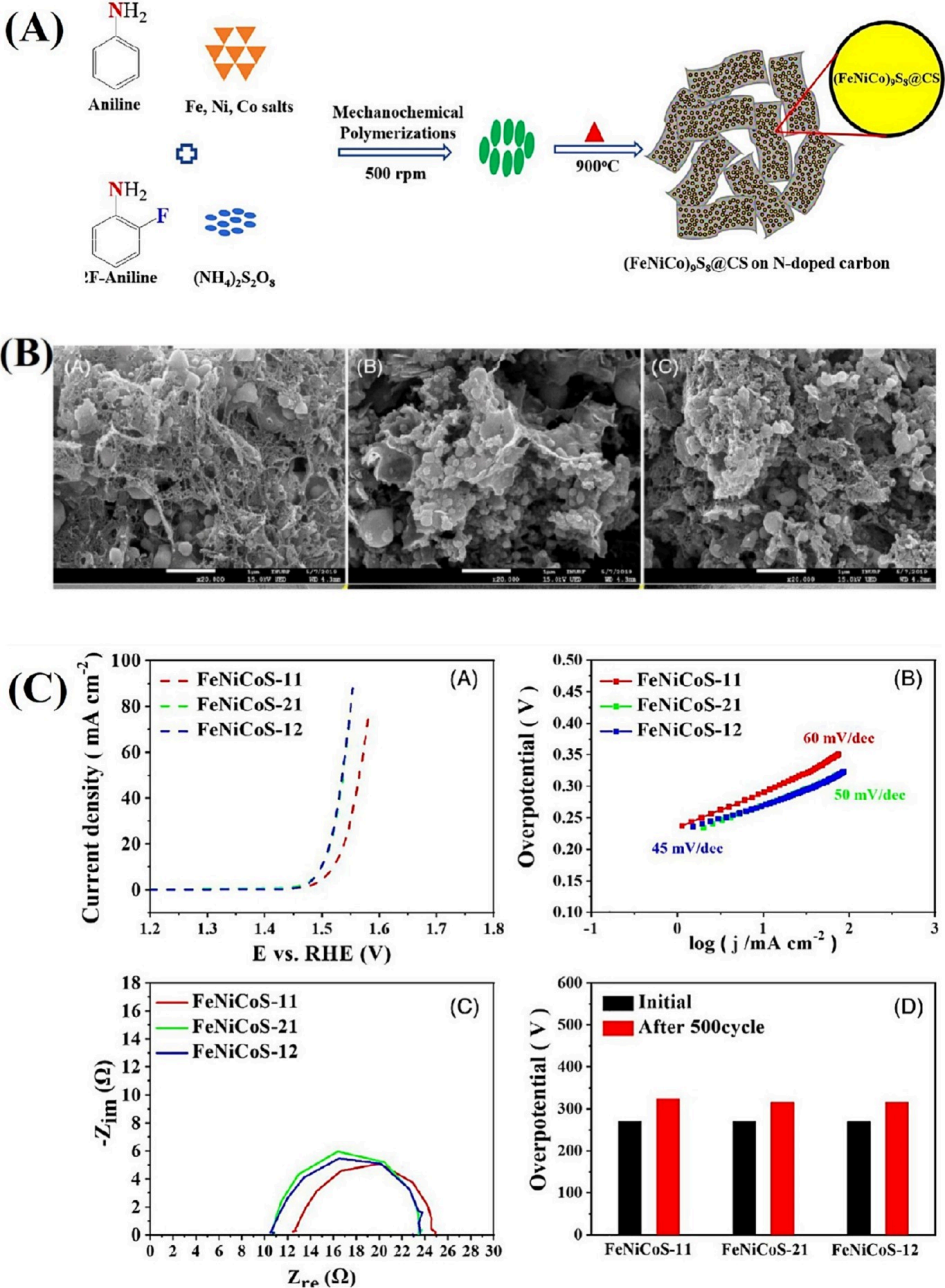
(A) Schematic illustration for the synthesis of (Fe, Ni, Co)_9_S_8_@CS-decorated N-doped carbon. (B) FE-SEM images of (A) FeNiCoS-21, (B) FeNiCoS-11, and (C) FeNiCoS-12. (C) (A) Polarization curves for OER and their corresponding (B) Tafel plots and (C) electrochemical impedance spectroscopy obtained at 10 mA/cm^2^. (D) Overpotential before and after 500 cycles of CV.

Copyright 2021, Wiley.^92^

### 3.4 Advancements in Trimetallic Electrocatalyst Design for Enhanced OER Performance

To enhance electrode performance, it is crucial to design electrodes with superior wetting and gas bubble-repelling properties, as oxygen bubbles can obstruct active sites and hinder reaction kinetics. A trimetallic (Fe, Co, Ni) spinel embedded in a carbon framework on nickel foam (FeCoNiOx/C/NF) was developed to improve OER efficiency. This 3D network structure harnesses superaerophobic and superhydrophilic properties in order to create a rapid release of bubbles and thus optimizes the triple-phase boundary (electrolyte, electrode, O_2_). This catalyst’s 3D network architecture was especially planned to increase OER effectiveness. Exploring how superaerophobic and superhydrophilic properties affect the electrochemical activity of the catalyst was the focus of the study. One of the considerations revolves around the fact that spinel-type oxides such as NiCo_2_O_4_ have attracted attention given their superior activity, corrosion resistance, general availability, and satisfactorily high electrical conductivity. Notably, Fe doping brought the benefit of a measurable specific surface area, as well as effective electron transport pathways, which improved the activity of nickel and cobalt supports/oxides. The FeCoNi/C/NF catalysts exhibited excellent performance and durability, maintaining stability over 250 h in alkaline conditions.^93^

Electrocatalysts based on palladium are gaining attention for cost-effective hydrogen production due to their strategic design and enhanced electrochemical performance in OER. This improvement stems from the synergy between Pd active sites and their support matrices, influenced by unique electronic configuration of Pd. Additionally, the introduction of Pd nanoparticles into different supports has been reported to enhance the catalytic activity, enabling thousands of catalytic sites nearby, which includes metal oxides, sulfides, and carbides dopes to increase active sites, charge carrier densities, etc. Incorporating Pd into Co–Fe oxides or nickel–iron layered hydroxides significantly boosts catalytic activity. Current research points toward possible improvement in the case of water and hydroxide adsorption for the OER intermediate conversion and O–O bond coupling when Fe is taken in as an additive either into the bulk or onto the surface.^99^ However, challenges like low conductivity and stability in alkaline environments under high current densities persist. To solve these issues, scientists are changing iron-based catalysts and using conductive carbon supports to improve electronic properties and life expectancy.^94^

OER electrocatalysis is particularly good in MIL-88A, an MOF with great surface area and adjustable pores and high stability. Creating ternary alloy-like MOFs with multimetallic building units may be an effective method to boost the intrinsic activity of pure MOFs without changing their structure, as the metal center has been identified as a potential active site that alters the properties of MOF. Incorporating multimetallic elements into catalysts enhances the availability of extra electrons from neighboring atoms and increases the number of active sites. A recent report details the in situ growth of diamond-shaped FeNiCo-MIL-88A structures on two-dimensional conductive Ti_3_C_2_Tx MXene. By precisely controlling the size and distribution of MOF particles, this technique produces a very homogeneous composite with a robust MOF-MXene connection. By mixing the metal ions uniformly with Ti3C2Tx before adding the linkers, the metal ions of the MOF were cross-linked with the surface termination groups of the Ti_3_C_2_Tx nanosheets. This broke the electrostatic repulsion of the Ti_3_C_2_Tx nanosheets and acted as nucleation sites for the in situ development of the MOF particles on the nanosheets, resulting in Ti_3_C_2_Tx/trimetallic MIL-88A composites (Figure 11A). As illustrated in Figure 11B, the laminar structure of an MXene, which features a high density of functional groups (−OH, −COOH) on its expansive surface, serves as an excellent platform for the in situ formation of MOFs. It is clear from Figure 11B that the morphology of FeNiCo-MIL resembles that of diamonds, and the solvothermal process led to the production of trimetallic MIL-88A diamonds in substantial quantities, which were densely coated onto Ti_3_C_2_Tx nanosheets, resulting in the formation of FeNiCo-MIL-coated Ti_3_C_2_Tx nanosheets (FeNiCo-MIL/Ti_3_C_2_). Figure 11C illustrates that the FeNiCo-MIL/Ti_3_C_2_ composite exhibits the highest OER activity among the tested electrocatalysts, demonstrating the lowest overpotential of 231 mV at a current density of 10 mA cm^–2^. This value is significantly lower than those observed for FeNiCo-MIL (1.490 V, η_10_ = 260 mV), NF (1.557 V, η_10_ = 327 mV), Ti_3_C_2_ (1.570 V, η_10_ = 340 mV), and commercial RuO_2_ (1.515 V, η_10_ = 285 mV). The incorporation of the synergistic effects from the three metals allows for a notable reduction in the overpotential of the OER, thereby enhancing both reaction rate and overall efficacy. Furthermore, FeNiCo-MIL/Ti_3_C_2_, FeNiCo-MIL, Ti_3_C_2_, RuO_2_, and NF had observed Tafel slopes of 34.5, 42.9, 81.2, 76.3, and 103.9 mV dec^–1^, respectively. Inserting trimetallic MOF diamonds between the MXene layers produced a composite with advantages like improved accessibility to active sites, superior conductivity, and significant synergistic and electronic coupling effects, in addition to the electronic interaction and/or cooperative effect between metal centers that was the primary factor in increasing the electrocatalytic activity.^124^

**Figure 11:**
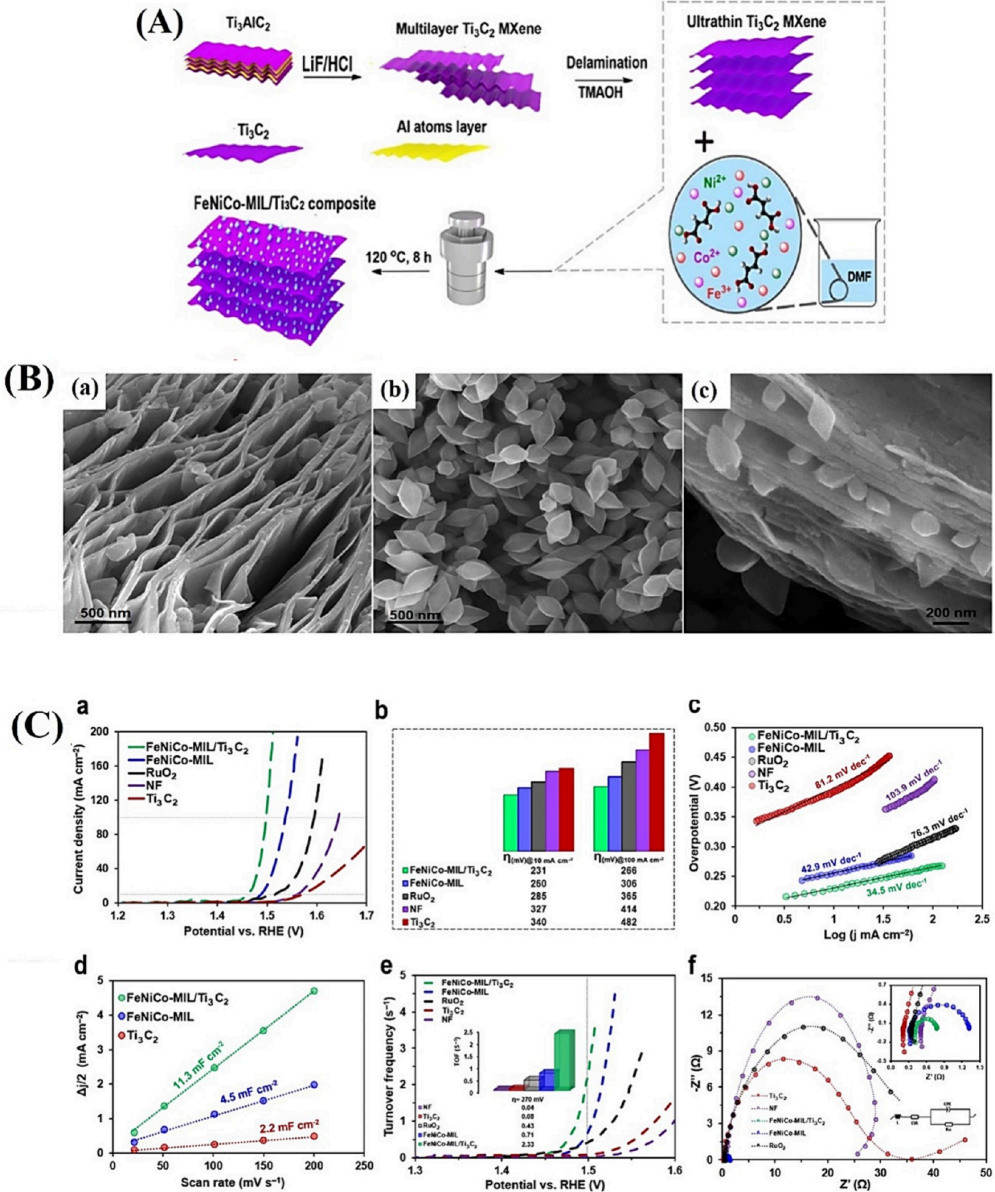
(A) Schematic description of the fabrication of the FeNiCo-MIL/Ti_3_C_2_ composite. (B) FESEM images of (a) Ti_3_C_2_ MXene, (b) FeNiCo-MIL diamonds, and (c) the FeNiCo-MIL/Ti_3_C_2_ composite. (C) OER performance of the samples (FeNiCo-MIL/Ti_3_C_2_, FeNiCo-MIL, commercial RuO_2_, nickel foam, and Ti_3_C_2_). (a) *iR*-corrected polarization LSV curves, (b) the comparison of the overpotential at *j* = 10 mA cm^–2^ and *j* = 100 mA cm^–2^, (c) corresponding Tafel plots, (d) *C*_dl_ estimated from the plot of capacitive currents in the middle of the potential window versus scan rate, (e) TOF versus potential (the inset shows TOF values calculated at an overpotential of 270 mV), and (f) EIS curves at 1.5 V vs RHE (inset shows the equivalent circuit).

Copyright 2024, RSC.^124^

MOF-74­(M) represents a class of MOFs formed through the coordination of various metal cations (M = Mg, Ca, Co, Ni, Zn, Fe, Mn, Cu) with 2,5-dioxido-1,4-benzenedicarboxylate (DOBDC). This MOF platform was selected for research because of its following special qualities: (1) the rod-shaped metal nodes’ type and composition can be easily changed, (2) the large one-dimensional hexagonal channels of MOF-74 facilitate rapid diffusion of reactants, allowing for efficient transformations within the framework even under challenging conditions, and (3) it features highly accessible unsaturated metal centers, where the metal atoms are not completely surrounded by the ligand’s native oxygen, thereby maximizing the density of active sites available for hosting reaction intermediates. Abdelhafiz et al. synthesized trimetallic MOF-74 and evaluated the impact of a specific metal (such as Ni, Co, or Fe) on OER activity, along with the associated structural and chemical properties.^125^ They examined different ratios of metals and found that using FeSO_4_ as the iron source in the synthesis not only stabilizes Fe^2+^ but also results in the creation of a pure trimetallic MOF-74 without any impurities. Figure 12A illustrates how altering the metals concentrations in the trimetallic NiCoFe MOF significantly impacts the performance, revealing that OER activity decreased with higher nickel and iron concentrations but increased with higher cobalt content. An analysis of the different compositions suggests that the Co ratio is essential for enhancing the activity and maximizing ECSA of the catalysts. To explore the effects of varying Co loading on activity and stability, it was found that the metal centers elevate their oxidation state under OER conditions. This change enhances metal–oxygen covalency, thereby facilitating the reaction. Furthermore, it was noted that there is a tendency for oxygen vacancy formation near the Co sites during the OER, which improves electronic conductivity and speeds up charge transfer kinetics (Figure 12A). Furthermore, in situ X-ray absorption spectroscopy (XAS) revealed that cobalt (Co) in the ternary NiCoFe MOF undergoes oxidation from Co^2^^+^ to a state closer to CoOOH under OER conditions, with the absorption edge shifting to higher energies in alkaline electrolyte and under applied biases (1.1 and 1.5 V vs RHE). The formation of oxygen vacancies and compressive strain around Co, as confirmed by X-ray absorption fine structure (EXAFS), enhances electronic conductivity and charge transfer, significantly boosting OER catalytic activity (Figure 12B).^125^

**Figure 12:**
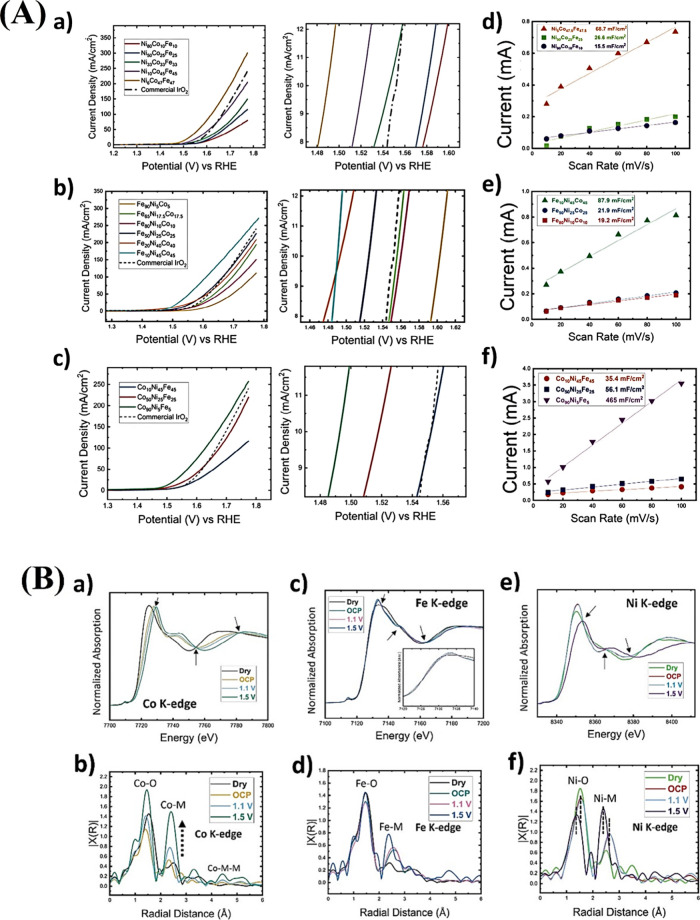
(A) Electrochemical OER test probed in O_2_-free 1 mol L^–1^ KOH electrolyte. (a–c) Non-*iR* compensated LSV scans of the three MOF-74, with the right panel showing LSV scans at 10 mA cm^–2^ current density. (d–f) ECSA of the families tested (a–c), respectively. Families tested: (a,d) Ni100–2xCoxFex, (b,e) Fe100–2xNixCox, and (c,f) Co100–2xNixCox. (B) In situ XAS spectra of trimetallic MOF-74 tested in an airtight 2-electrode cell with 1 mol L^–1^ KOH electrolyte. (a,b) XANES and EXAFS of Co K-edge spectra, respectively. (c,d) XANES and EXAFS of Fe K-edge spectra, respectively. (e,f) XANES and EXAFS of Ni K-edge spectra, respectively. In situ scans were performed under different conditions: as-synthesized (dry), OCP, pre-OER (1.1 V vs RHE), and around OER onset (1.5 V vs RHE).

Copyright 2024, Wiley.^125^

Promising OER catalysts with zeolitic imidazolate frameworks (ZIFs), a subclass of MOFs, are characterized by their tunable architecture and improved properties. Converting ZIFs into yolk–shell or core–shell structures integrates active metal sites and custom ligands therefore increasing catalytic performance. With changes including metal sulfides,^126^ oxides, and nanoalloys,^127^ ZIF-67 and its derivatives are excellent for OER electrocatalysis, thus improving output even more. By utilizing its well-defined cavity and functional shell, the HS-ZIF-67 approach was used to synthesize a series of trimetallic CuCo-based oxides supported on C–N networks. The microstructure was optimized through cation exchanges (introducing Cu, Fe, Ni, or Zn) and thermal treatments. The hollow structure of these ternary oxides improves mechanical and chemical stability, increases active site exposure, decreases electrolyte migration paths, and improves OER performance, outperforming binary and monometallic oxides (Figure 13A). Among them, HS-CuFeCoO_*x*_ exhibited the best performance with a low overpotential of 309 mV and a Tafel slope of 71.4 mV dec^–1^ at 10 mA cm^–2^, outperforming HS-CuNiCoO_*x*_ (341 mV), HS-CuCoO_*x*_ (354 mV), HS-CuZnCoO_*x*_ (362 mV), and HS-Co_3_O_4_ (405 mV). Its unique microstructure and metallic content account for its exceptional activity; stable spherical form and early current response are confirmed by SEM (Figure 13B) and LSV (Figure 13C). The smallest Tafel slope (71.4 mV dec^–1^ vs 73.1 and 80.1 mV dec^–1^) highlights the fastest OER kinetics of HS-CuFeCoO_*x*_, aided by its ultrathin shell and larger ECSA, while agglomeration in HS-CuZnCoO_*x*_ reduces its active surface. The hollow structure enhances performance with a larger surface area and reduced electrolyte pathways.^128^

**Figure 13:**
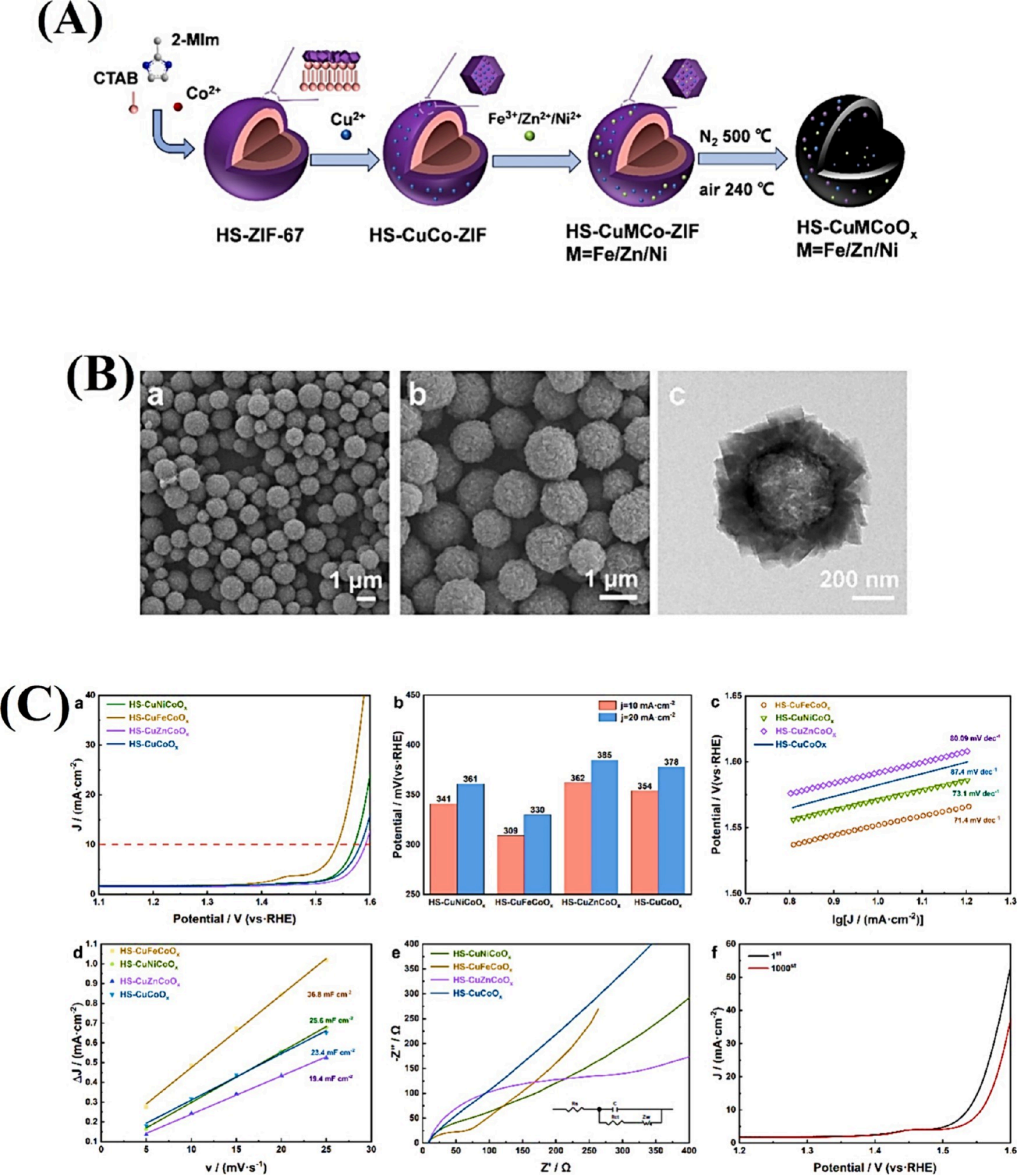
(A) Synthetic approach of the HS-CuMCoOx (M = Fe/Zn/Ni) composites. (B) SEM images of (a) HS-CuCo-ZIF, (b) HS-CuFeCo-ZIF; HRTEM images (c). (C) Electrochemical characterization of the prepared samples for OER. (a) LSV curves after 95% *iR* compensation, (b) overpotentials at the specific current densities, (c) Tafel plots, (d) capacitive current densities of the different catalysts at different scan rates, (e) Nyquist plots of impedance spectroscopy analysis of the as-prepared electrocatalysts in 1 mol·L^–1^ KOH, and the inset in panel (e) showing the fitted equivalent model for the impedance tests. (f) LSV curves of 1st and 1000th cycles.

Copyright 2024, Elsevier.^128^

MOFs provide two approaches for electrocatalysis: (1) as templates for manufacture of porous materials including metal, oxides, and carbon composites^129^ and (2) as direct catalysts.^130^

Multivariate MOFs (MTV-MOFs) introduce several metal ions to address the problem of poor electronic conductivity, thereby improving their catalytic, optical, and electronic properties by synergistic effects. This technique greatly improves electrocatalysis.^131–133^ Manganese doping improves electronic properties, while cobalt and nickel, widely found and highly catalytically effective, abound in OER catalysts.^134,135^ When used as catalysts, combining Ni, Co, and Mn should yield good OER performance. With excellent stability, a low overpotential of 220 mV at 20 mA cm^–2^, and a Tafel slope of 66 mV dec^–1^, the trimetallic CoNiMn-MOF displays strong electrocatalytic performance.^97^ Through OER, transition-metal nanomaterials with low-electronegativity heteroatoms turn into (oxy)­hydroxides and oxides while maintaining good conductivity. The energy level is lowered using intermediate binding tuning, thus improving catalytic effectiveness by maximizing the key OER step.^136^ Multimetal nonoxides that have been structurally optimized expose more active sites, therefore improving OER performance. Hierarchical porous nanomaterials with many electrical sites, reduced charge/mass transfer pathways, and improved adsorption–desorption rates help to optimize the reactive sites for effective OER catalysis by decreasing electrolyte diffusion and charge transfer barriers.^137,138^ Changing crystal structures such as using amorphous or low-crystalline forms raises active site exposure and unsaturated site density because of a special combination of long-range disorder and short-range order, therefore lowering energy barriers for reactant adsorption.^139^ Furthermore, heterointerfaces affect the speed of OER kinetics, and polycrystalline amorphous structures frequently exhibit higher oxidation states at catalytic sites. Progress notwithstanding, the creation of mixed-transition metal nonoxides with hierarchical porosity for OER catalysis remains hard and little explored.

Trimetallic CoFeMo-A (A = P, Se) nonoxides, with a hierarchically porous structure derived from ZIF-67 LDH, use cobalt as the primary matrix. The integration of iron and molybdenum promotes electron delocalization, creating active sites. Phosphorus and selenium form metal phosphides/selenides, which can convert to oxide/(oxy)­hydroxides during OER, enhancing catalytic performance of CoFeMo-A while preserving electronic conductivity and generating novel active sites.^98^

Transition metal oxides (TMOs) optimize electrocatalytic performance through interfacial interactions, microstructure manipulation, and increased active site exposure. Particularly because of cobalt and iron incorporation, transition metal ferrites especially Co_2_FeO_4_ are remarkable for their gyromagnetic properties and increased hardness. Cobalt ferrite oxide emerges as a distinctive compound within binary spinel systems due to its array of desirable physical and chemical properties. The Kirkendall effect changes strong Co_2_Fe nanoparticles into hollow Co_2_FeO_4_ particles, thereby increasing active points and enhancing interactions between electrolyte and catalyst, which greatly improves electric performance by means of electrocatalysis. Incorporating Fe^3+^ into Co_3_O_4_ spinel activates Co^3+^ sites by dispersing Co 3d electrons, while doping with metals or oxides fine-tunes shape, architecture, and conductivity for electrochemical applications.^140^ Adding PdO as a dopant further improves catalytic efficiency of Co_2_FeO_4_ by increasing active sites, reducing band gap, and boosting charge transfer.^141^ Moreover, at high temperatures, PdO severs adsorbed H_2_ and O_2_ to make reactive H and O atoms. Co_2_FeO_4_@PdO nanostructures created by hydrothermal synthesis (Figure 14A) display a convoluted spherical and cubic morphology, hence increasing electrocatalytic efficiency even more (Figure 14B).^99^ In comparison to Co_2_FeO_4_, the Co_2_FeO_4_@PdO material exhibited improved conductivity and enhanced surface properties, characterized by smaller particles in both spherical and cubic shapes. These particles are further contributing to stronger electrochemical characteristics and offer a broad surface for the rapid activity of water molecules during electrolysis as well as improved conductivity. Illustrated in Figure 14C, the overpotential values at a current density of 20 mA cm^−2^ are 293 mV for Co_2_FeO_4_, 259 mV for Co_2_FeO_4_@PdO, and 210 mV for RuO_2_. Tafel slope values were calculated as 67, 59, and 52 mV dec^–1^ for Co_2_FeO_4_, Co_2_FeO_4_@PdO, and RuO_2_, respectively. The lower Tafel slope of Co_2_FeO_4_@PdO, which is comparable to that of precious metal-based electrocatalysts, indicates its improved kinetics. This enhanced performance can be attributed to several factors, including the composite’s high active surface area of 395 cm^2^, structural irregularities, the presence of oxygen vacancies, rapid charge transport, and the significant exposure of cobalt ferrite oxide active sites on the palladium oxide surface. Using reduced graphene oxide (rGO), Niyitanga and Kim created supported trimetallc Cu_*x*_Mo_*x*_/Co_1–*x*_O nanoparticles for OER. Their outstanding electrocatalytic efficiency is shown by overpotentials of 269 mV and 259 mV at 10 and 20 mA cm^–2^, respectively. Doping cobalt oxide with high-valence Mo^6+^ ions modifies the electronic structure of the 3d metal due to the ability of Mo^6+^ to attract electrons. Incorporation of Cu and Mo dopants creates bridged active sites, bolstering catalytic performance in OER applications. Additionally, RGO has garnered significant attention as a promising support matrix for semiconducting transition metal oxide compounds, thanks to its high electronic conductivity and large specific surface area, which can enhance their electrocatalytic properties for OER. Via interface engineering, the strategic synergy between cobalt-based oxides and conductive materials improves charge-transfer kinetics, stimulates electric activity, and notably increases OER catalytic efficiency. By customizing electronic configurations and maximizing oxygen adsorption, heterobimetallic oxides improve electrocatalytic properties. Improving charge transfer raises the OER performance of Co-based oxides by engineering heterostructures with active sites. The Cu_0.19_Mo_0.19_/Co_0.62_O NPs@RGO hybrid catalyst outperformed CoO NPS (430 mV), CoO NPS@RGO (410 mV), Mo_0.38_/Co_0.62_O NPS@RGO (390 mV), Cu_0.38_/Co_0.62_O NPS@RGO (340 mV), Cu_0.1_Mo_0.28_/Co_0.62_O NPS@RGO (290 mV), and Cu_0.28_Mo_0.1_/Co_0.62_O NPS@RGO (270 mV), in this investigation with a low overpotential of 250 mV at 10 mA cm^–2^, exhibiting the best OER performance. The highly conductive RGO nanosheets, Cu and Mo dopants, and Co active sites work in concert to improve conductivity and electron transport, which accounts for this improved activity. In addition, the catalyst showed the lowest Tafel slope of 61 mV dec^–1^, indicating optimal electrocatalytic kinetics, in contrast to Cu_0.28_Mo_0.1_/Co_0.62_O NPs@RGO (63 mV dec^–1^), Cu_0.38_/Co_0.62_O NPs@RGO (75 mV dec^–1^), Mo_0.38_/Co_0.62_O NPs@RGO (82 mV dec^–1^), CoO NPs@RGO (92 mV dec^–1^), and CoO NPs (94 mV dec^–1^).^100^ The synergistic impact is maximized by the equal concentration of bimetallic doping, which also improves catalytic kinetics and aids in the creation of large-scale, reasonably priced water-oxidation catalysts for hydrogen production.

**Figure 14:**
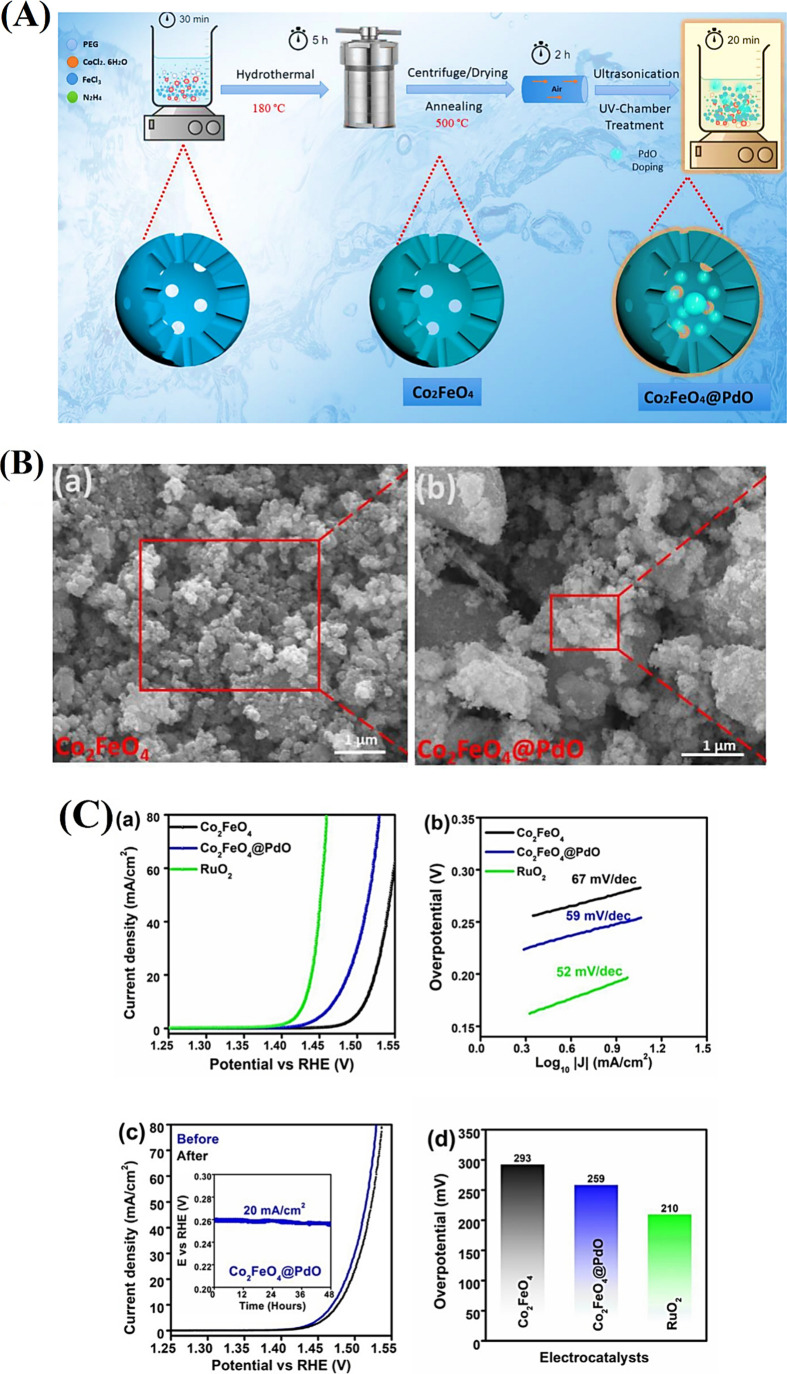
(A) Overall experimental illustration toward the final product (Co_2_FeO_4_@PdO. (B) SEM images of (a) Co_2_FeO_4_ and (b) Co_2_FeO_4_@PdO. (C) (a) LSV curves for OER; Co_2_FeO_4_, Co_2_FeO_4_@PdO, and RuO_2_. (b) Corresponding Tafel slope values. (c) Durability and chronopotentiometry at 20 mA cm^−2^ current density (as given in the inset). (d) Histogram for overpotential values.

Copyright 2023, Elsevier Inc.^99^

## 4 Enhancing HER Efficiency for Trimetallic and Derived Materials

### 4.1 Alternative Catalysts for HER

Hydrogen evolution reaction also poses challenges since it suffers from sluggish kinetics, high overpotentials, and dependence of expensive noble metal catalysts such as platinum.^142^ Ongoing research efforts are concentrated on the development of lower cost, more abundant, non-noble metal-based, eco-friendly catalyst alternatives. The main goal is to maximize catalyst activity, efficiency, and stability while elucidating electronic structures (the theory and mechanism) and surface interactions to enhance H_2_ production. Although platinum is the most efficient HER catalyst with good stability and meager onset potential, its high cost restricts its widespread use.

First-row transition metal-based trimetallic electrocatalysts have recently emerged as promising candidates for HER. In situ construction of multiphase electrocatalysts is a promising approach to enhance catalytic activity by taking advantage of the synergistic effects of transition metals such as Cu, Ni, Fe, Co, Mn, and Mo (Table 2). This optimizes the electronic properties in alkaline media, facilitating hydrogen production efficiency via selectively tuned reaction intermediates.^143^ While being inert on their own, the alloyed systems using platinum combined with relatively inexpensive transition metals (Ni, Fe, Cu, Co) largely form bimetallic Pt-based catalysts, which further pertain to the adjustment of lattice parameters and electronic structures that lower adsorption energy and enhance the activity.^144^ The introduction of a tertiary transition metal in the bimetallic platforms augments catalyst performance and structural relative stability, resulting in a viable area of catalytic exploration. Many systematic research studies on the unique capabilities of copper-based compounds in electrocatalytic and photocatalytic procedures for HER have caught the attention of the scientific community.^145,146^ In this scenario, copper-based compounds containing both bimetallic and trimetallic configurations demonstrate high current densities with low overpotentials, thus rendering them as viable substitutes for platinum.^147^ Recent studies have broadly focused on copper-containing materials as promising candidates to replace platinum as sustainable alternatives for green hydrogen production and reductions of carbon emissions.^148^ PtCuNi nanoparticles were synthesized via a modified polyol method, enabling the efficient production of oxide-free nanocrystals at lower temperatures and precise control over the Pt, Cu, and Ni composition ratios, thereby enhancing the catalytic properties. The Pt_39_Cu_24_Ni_37_ catalyst apparently showed excellent HER activity over all the compositions (Pt_21_Cu_55_Ni_24_ - 257.8 mV and Pt_45_Cu_32_Ni_23_ - 207.33 mV) when evaluated in 1 mol L^–1^ KOH, with a superior onset potential of 30.3 mV (vs RHE) and overpotential of −71.30 mV at 10 mA cm^–2^, respectively.^149^ The highest current densities for HER activity at the same potential are in the order of Pt_39_Cu_24_Ni_37_ > Pt_45_Cu_32_Ni_23_ > Pt_21_Cu_55_Ni_24_. Based on microstrain and structural characteristics, the results show that a larger PtNi ratio in ternary catalysts increases HER activity. With the greatest PtNi ratio, the Pt_39_Cu_24_Ni_37_ alloy demonstrated excellent catalytic (onset potential, overpotential, ECSA) and structural (dislocation density, microstrain) performance. Two factors contribute to this increased HER activity: (1) doping Pt with Cu and Ni changes its electronic structure, shifting the d-band center and decreasing hydrogen binding strength, improving HER efficiency according to d-band theory; and (2) synergistic effects, where Pt facilitates the adsorption and conversion of hydrogen intermediates to H_2_ while Ni and Cu initiate water decomposition, overcoming the limitations of each metal individually.

**Table 2:** HER Performance of Trimetallic Electrocatalysts in Alkaline Simulated Seawater

catalysts	electrolyte/concentration (mol L^–1^)	substrate	(mA cm^–2^)	η (mV)	Tafel slope (mV dec ^–1^)	ref
PtCuNi	KOH/1	CRE^a^	10	71.3	50	^149^
Pt-CoNi	KOH/1	NF	50	116	75	^152^
H-PtNiCu-AAT	HClO_4_/0. 1M	GCE	10	32	33	^153^
Pt1Ni_2_@NC	HClO_4_/0. 1M	RRDE^b^	10	28	71.1	^150^
(Fe,Co,Ni)_9_S_8_	H_2_SO_4_/0.5		50	320–490	114	^154^
Fe_3_Co_3_Ni_3_S_8_/carbon	H_2_SO_4_/0.5	GCE	1	329	2	^155^
(Co,Fe,Ni)_9_S_8_–xSex	H_2_SO_4_/0.5	TM3Ch4	120	240	110	^156^
Ni–Co–W phosphoxide	KOH/1	PNCF^c^	53	53	40	^157^
Cu_2_CoSnS_4_	H_2_SO_4_/0.5, KOH/1, and PBS/1	paper	10	233, 310, and 261	110, 120, and 273	^158^
MoSe_2_–NiSe_2_–CoSe_2_	KOH/1	PNCF	10	38	38	^159^
N-HPCS@Co–Cu–Fe	KOH/1	GCE	10	114	73.72	^160^
Ni_3_S_2_/MoS_2_/Fe_3_S_4_	KOH/1	NF	10	54	86.5	^161^
CoNiMn–OH/	KOH/1	CC	10	202	212	^162^
AuAgCu	KOH/1	GCE	10	negligible overpotential	32.7	^163^
CuO/Ag/NiO	H_2_SO_4_/0.5	GCE	10	100	270	^164^
Pt-NiFe-LDH	KOH/1	NF	50	63	73	^165^
Co_3_O_4_@NiFe-LDH	KOH/1	NF	10	79	100.1	^166^

a Carbon rod electrode.

b Rotating ring disc electrode.

c NiCo foam (NCF).

PtNiCo ternary alloys were loaded in N-doped porous carbon (NC) with high specific surface area and abundant pore structure. A straightforward method is used to create porous carbon as a matrix, which is then alloyed with transition metals at high temperatures and doped with nitrogen to create highly distributed and robust PtNiCo ternary alloy catalysts (Figure 15A). According to SEM data, Pt_1_@C displays a polyhedral morphology and agglomerated huge lumps because of the lack of additives; for example, urea was not present during pyrolysis. Meanwhile, Ni_2_Co@NC displays uniform polyhedral structures with low agglomeration. On the other hand, Pt_1_@NC exhibits smaller, more evenly distributed structures, which are explained by nitrogen-induced strain during annealing. Pt_1_Ni_2_Co@NC has a uniform, evenly distributed polyhedral shape and is created by doping with transition metals and nitrogen (Figure 15B). In acidic electrolytes with higher reaction rates, Pt_1_Ni_2_Co@NC and Pt_1_Ni_2_@NC exhibited higher performance than Pt/C, according to the HER overpotentials at 10 mA cm^–2^ for Pt_1_Ni_2_Co@NC (26 mV), Pt_1_Ni_2_@NC (28 mV), Pt_1_@NC (52 mV), Pt_1_@C (38.84 mV), and commercial Pt/C (35.53 mV). Superior HER kinetics are indicated by their reduced Tafel slopes (30.14 mV dec^–1^ for Pt_1_Ni_2_Co@NC and 35.00 mV dec^–1^ for Pt_1_Ni_2_@NC) compared to Pt/C (35.53 mV dec^–1^), Pt_1_@NC (30.03 mV dec^–1^), and Pt_1_@C (38.84 mV dec^–1^). The high specific surface area, porous structure, and exposed active sites of PtNiCo@NC contribute to its exceptional performance by improving reaction kinetics and charge transfer. A stable active surface is guaranteed by the robust anchoring of Pt-based ternary alloy nanoparticles to the Ni/Co–N-doped nanoporous carbon substrate, which increases catalyst stability in the acidic environment. According to the results of the DFT theoretical analysis, the nearby Ni and Co atoms are crucial in fostering the contact between Pt atoms and adsorbed hydrogen (Figure 15C).^150,151^

**Figure 15:**
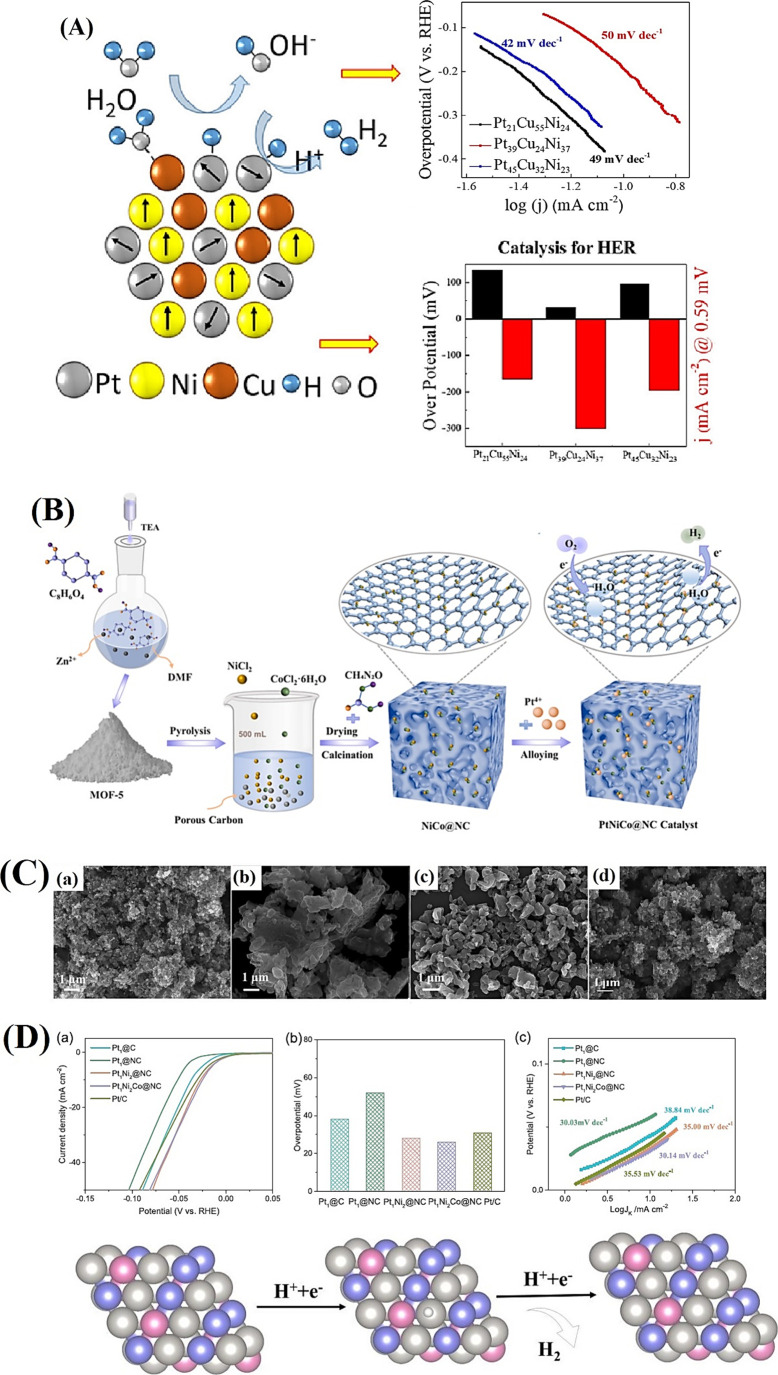
(A) The schematic of PtNiCu and Tafel slopes for the HER activity of Pt_21_Cu_55_Ni_24_, Pt_39_Cu_24_Ni_37_, and Pt_45_Cu_32_Ni_23_ catalysts at a potential of 93.3 mV (vs RHE) overpotential.(B) Schematic illustration for the synthesis procedure of the PtNiCo@xNC catalyst. (C) SEM images of (a) Ni_2_Co@NC, (b) Pt_1_@C, (c) Pt_1_@NC, and (d) Pt_1_Ni_2_Co@NC catalysts. (D) (a) HER polarization curves, (b) corresponding overpotentials (10 mA cm^–2^), and (c) Tafel plots of Pt_1_@C, Pt_1_@NC, Pt_1_Ni_2_@NC, Pt_1_Ni_2_Co@NC, and Pt/C in 0.5 mol L^–1^ H_2_SO_4_. HER adsorbed atomic structural model of Pt_15_Ni_10_Co_7_ (111). Atoms are Pt (gray), Ni (purple), Co (pink), O (yellow), and H (white).

Copyright 2024, Elsevier Inc.^149^

Copyright 2023, Elsevier Inc.^150^

Sulfides, nitrides, phosphides, selenides, and hydroxides are transition metal compounds whose tunable properties have caused outstanding hydrogen production and further sustainable energy.^167^ Thanks to their great surface areas, carbides, phosphides, sulfides, borides, and first-row transition metals’ heterostructures increase charge transfer and active site density; hence, they are appealing for electrocatalysis.^168^

Pentlandites with a structure of M_9_X_8_, where M denotes a transition metal and X is a chalcogenide, are good alternatives to platinum for electrocatalysis. These materials have great performance for HER and stability for inhibitors such as H_2_S and CO (Figure 16). In trimetallic nanostructures containing cobalt, iron, and nickel cations, the electronic structure is modified through the interplay of d- and p-orbitals, resulting in an increased density of states close to the Fermi level. This synergy strengthens the metallic character of the trimetallic catalyst, facilitates effective charge transfer, and increases the active surface area, significantly improving the efficiency of HER and making these materials ideal for advanced electrocatalytic water splitting.^169^ With the assistance of modification or saturation with other first-row 3d transition metals (cobalt, iron, and molybdenum), the transition metal disulfides can also exhibit considerable catalytic activity with 10 mA cm^–2^ delivered at moderately low overpotentials. Pentlandite’s high catalytic activity is thus attributed to intermetallic interactions, unique sulfur anions, and sulfur vacancies, which provide active sites that exhibit strong binding to hydrogen atoms, particularly at the 8c Wyckoff sites. Smialkowski et al. prepared trimetallic pentlandites (Fe, Co, Ni)_9_S_8_ with different metal ratios and efficiently tested them as acidic HER catalysts.^154^ Tetzlaff and colleagues introduced a mechanochemical method to produce pentlandite-carbon composites (Fe_3_Co_3_Ni_3_S_8_/C), achieving a remarkable current density of 1 A cm^–2^ at a low cell potential of 2.12 V in a zero-gap proton exchange membrane (PEM) electrolyzer.^155^ Pentlandite exhibits excellent HER performance, with a minimal overpotential of 240 mV at 120 mA cm^–2^ and a Tafel slope of 70 to 110 mV dec^–1^, along with robust stability in corrosive environments.^156^ Indeed, the cationic arrangement is amenable to adjustment in materials with high entropy architectures, and this has been leveraged in achieving dramatic changes in electronic structure and resulting electrocatalytic behavior via band centers and occupancy distribution of bonding or antibonding orbitals, as well as bond covalencies and orbital interactions.^170^ One such promising approach to enhance catalytic activity and pursue catalysis research is optimizing the compositions of multicomponent transition metal chalcogenides (such as the sulfides/selenides of Co, Fe, and Ni), especially by tuning the cation ratios and the sulfur/selenium ratios of pentlandite.

**Figure 16:**
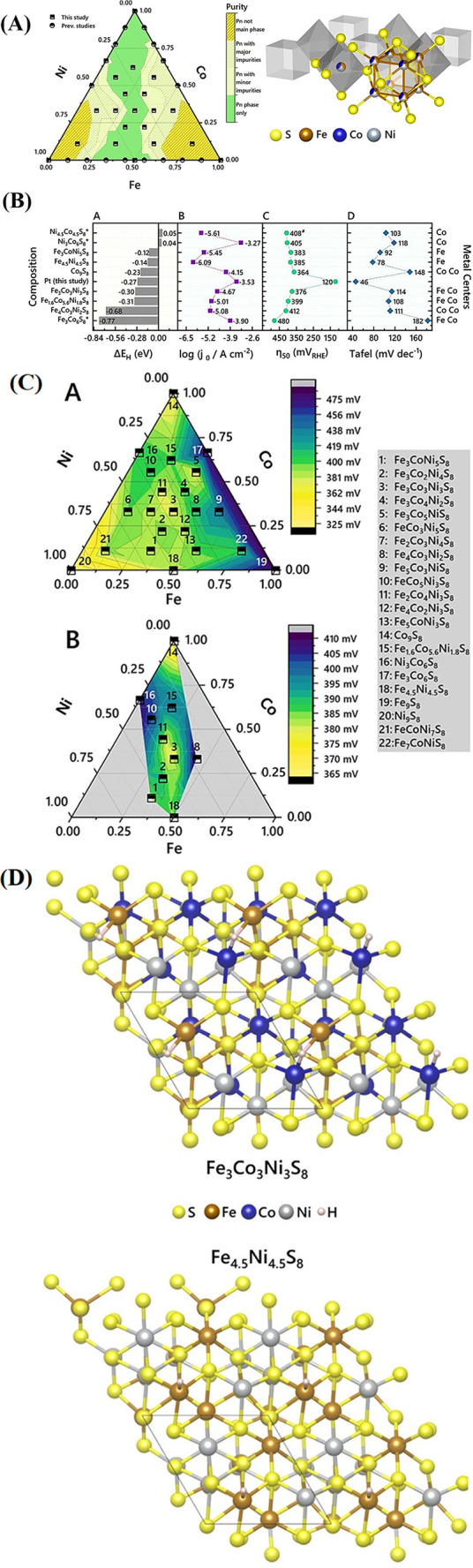
(A) Ternary plot shows the Fe–Co–Ni sulfide system including various M_9_S_8_-type materials from this and previous studies and gives relation to phase-purity via a colorized topology map. Crystal structure represents the Fe_3_Co_3_Ni_3_S_8_ pentlandite structure. (B) Graph showing hydrogen adsorption energy Δ*E*_H_, common logarithm of exchange current density log­(*j*_0_), overpotential η_50_ at 25 °C (# = value read at −47 mA cm^–2^), and Tafel slope of different compounds. (C) Ternary plots of the Fe–Co–Ni sulfide system. The topology maps give the initial η_50_ values from LSV at 25 °C. All materials and composites of the study and only a selection of almost phase-pure compounds. (D) 2 × 2 × 1 supercell of pentlandite surfaces with adsorbed hydrogen atoms. Fe_3_Co_3_Ni_3_S_8_ comprises μ2-H, while at the Fe_4.5_Ni_4_._5_S_8_ surface, the H is terminally bound. The rhombus marks the DFT-calculated unit cell.

Copyright 2022, ACS Inc.^154^

### 4.2 Advanced Catalyst Design for HER

TMPs are smart candidates for HER electrocatalysis due to hydrogen bonding, d electron configuration, corrosion-resistance, and conductivity properties. Their efficiencies in catalysis can capitalize on structural resemblances to hydrogenases in which the metals and phosphorus act as proton and hydride manipulators.^171^ Their HER efficiency has been observed to lag far behind noble-metal catalysts. However, heteroatom-doping with transition metals, especially tungsten, was explored for more efficient electron occupancy and the emergence of hybridized orbitals close to the Fermi level that enhance charge transfer dynamics and HER properties.^172^ Due to tungsten’s considerable electron-donating nature, the corresponding downward energy-level shifts in the e_g_ orbitals render hydrogen adsorption even tighter, which probably lowers free energy for adsorbed species. The electronic properties of TMPs can be optimized by means of heterostructure engineering and carefully doping to minimize the hydrogen adsorption free energy. There exists already clear synergy in nickel, cobalt, and tungsten phosphide interfaces to improve catalytic activity through the combined lowering of Gibbs energy for hydrogen ion adsorption. Zhang and co-workers then highlighted an optimal metal-phosphide combination sufficient to enhance H_2_ adsorption and desorption. They synthesized octahedron-shaped M_*x*_O@M_*x*_P hybrids starting from Ni–Co foam, initially plasma-treated with a dielectric barrier discharge to transform the alloy into compounds, thus providing defects on the PNCF surface to provide active sites for reactions (Figure 17A). They later applied a hydrothermal method with Ni, Co, and W on the PNCF, followed by phosphorization with elemental phosphorus. The resulting NiCoWO_4_/PNCF composite featured well-defined octahedral particles, averaging 4–5 μm, uniformly dispersed on the PNCF substrate (Figure 17B). The phosphide catalyst MxO@MxP/PNCF performs exceptionally well in HER with an overpotential of 53 mV at 10 mA cm^–2^. It surpasses other catalysts such as NiCo WO_4_/PNCF (77 mV), NiWO_4_/PNF (140 mV), PNCF (141 mV), and NCF (181 mV). Its Tafel slope of 40 mV dec^–1^ is notably lower than those of NiCoWO_4_/PNCF (86 mV dec^–1^), NiWO_4_/PNF (144 mV dec^–1^), PNCF (146 mV dec^–1^), and NCF (166 mV dec^–1^) and is comparable to that of Pt/C (37 mV dec^–1^), suggesting fast HER kinetics (Figure 17C).^157^ In alkaline solution, the reaction follows the Volmer–Heyrovsky mechanism, where the rate-limiting Volmer step consists of water dissociation and the Heyrovsky step promotes the electrochemical desorption of hydrogen. Ni-CoP and Ni-WP_2_ exhibit lower reactive Gibbs energies for HER, according to DFT calculations. By fostering a high density of H* intermediates for quick H_2_ bubble release and effective H^+^ adsorption on evenly distributed nanosized hole edges of its micrometer-scale octahedron surface, the MxO@MxP/PNCF electrocatalyst demonstrates remarkable activity and stability (Figure 17D).

**Figure 17:**
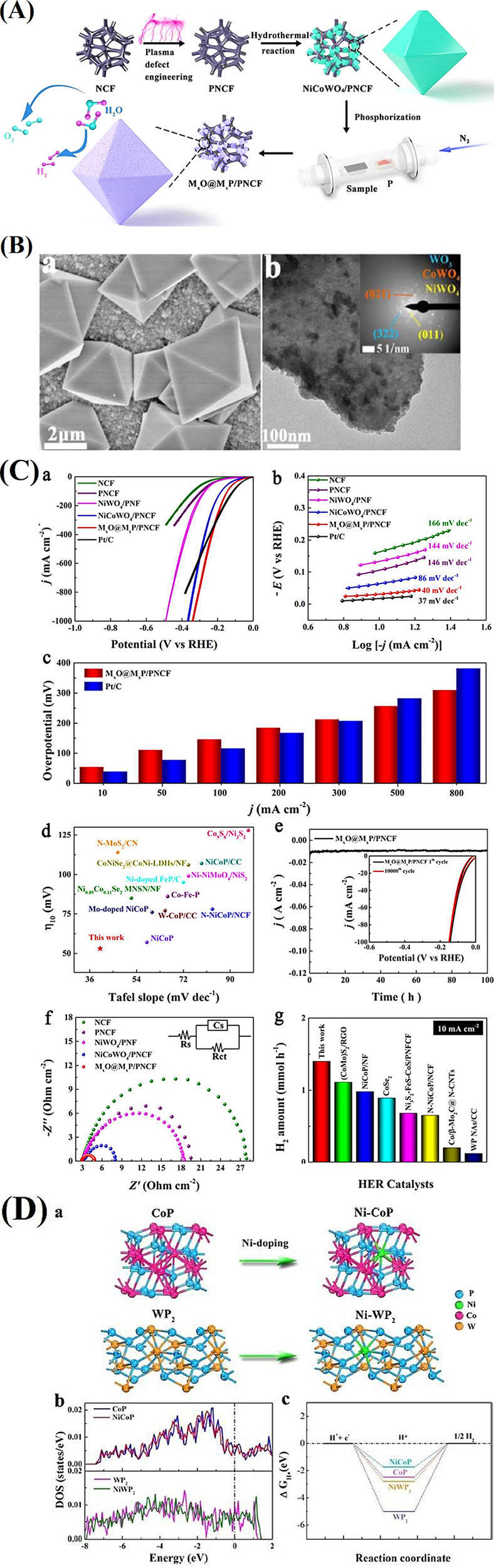
(A) Fabrication of a multiphase, trimetallic Ni–Co–W phosphoxide electrocatalyst: Regular octahedral multiphase MxO@MxP crystals are formed in situ on a plasma-defect-engineered Ni–Co support. (B) SEM analysis of the NiCoWO_4_/PNCF. (C) HER performance of the obtained electrocatalysts vs existing state of the art. (a) CV curves, (b) Tafel slopes, (c) outperforming the Pt/C benchmark at industry-relevant current densities above 300 mA cm^–2^, (d) overpotential comparison with Tafel slopes for delivering *j*_10_, (e) electrocatalytic stability of MxO@MxP/PNCF and the inset is the sustainability test, (f) Nyquist curves, and (g) generation amount of H_2_ for different catalysts at *j*_10_. (D) Mechanisms for HER enhancement supported by ab initio DFT simulations. (a) Crystal structures of the CoP, Ni-CoP, WP_2_, and Ni-WP_2_. (b) Calculated DOS of the resulting catalysts. (c) H* Gibbs free energy of different electrocatalysts.

Copyright 2021, ACS.^157^

Key for use in acidic, neutral, or basic electrolytes such as proton exchange membranes, microbial electrolysis, and industrial water electrolysis is the development of electrocatalysts designed to be active and stable across a wide pH range. Via the electrospinning method, Ozel and partners were able to effectively create trimetallic Cu_2_CoSnS_4_ nanofibers for boosting the electrocatalytic performance in hydrogen evolution reactions at interfaces having flexible properties.^173^ Joshi et al. explored Cu_2_CoSnS_4_-embedded paper substrates for upscaling HER applications, integrating state-of-the-art electrode materials within a paper-based platform.^158^ Cu_2_CoSnS_4_ is synthesized by a solvent-free solid-phase method and employed to create a paper-based working electrode for HER (Figure 18A). The electrode shows structural improvements: (i) Replacing Zn^2+^ with Co^2+^ in Cu_2_ZnSnS_4_ lowers antisite defects, hence enhancing HER activity. While tin supports hydrogen dissociation, copper provides erosion resistance, conductivity, and mechanical strength. (ii) Lowering electronegativity and d-band properties of cobalt improves hydrogen binding at active sites. Excessive binding can, however, saturate these sites, deactivate catalysts, and lower HER activity. Maximizing HER activity depends on managing adsorption strength at active sites. By changing binding energies, Cu, Sn, and S elements improve HER by acting on binding interactions: copper has lower binding energy as a result of its sp band features; sulfur’s higher electronegativity renders it perfect for adsorbing intermediates and assisting hydrogen desorption. Monometallic catalysts typically have difficulty in stabilizing intermediate molecules with ideal binding energies, which lowers HER performance. Maintaining reaction routes depends on having vital techniques to keep several intermediates stable. Paper can be used as a catalyst support and offers great benefits: its porous nature, hydroxyl/epoxy functional groups, and versatility allow strong adsorption, fast mass transfer, and pH-resilience. This gives paper-based electrodes excellent longevity and performance for HER, which makes them very effective.^174^ The SEM photographs of the obverse and reverse sides of the Cu_2_CoSnS_4_-decorated paper working electrode indicate dense agglomeration of intertwined nanoparticles that interfolds with complex interaction with the porous architecture of the paper substrate (Figure 18B). Composite architecture creates a labyrinthine system in which the porous structure of Cu_2_CoSnS_4_ hosts high amounts of catalytically active edges. The strong observability of the active edges contributes to augmenting the electron transfer kinetics for superior interoperation among the electrocatalyst and the electrolytic environment. Cu_2_CoSnS_4_ paper electrodes had low overpotentials of 233 mV (0.5 mol L^−1^ H_2_SO), 310 mV (1 mol L^−1^ KOH), and 261 mV (1 mol L^−1^ PBS) at 10 mA cm^–2^, with large double-layer capacitance and active surface area across pH values (Figure 18C).

**Figure 18:**
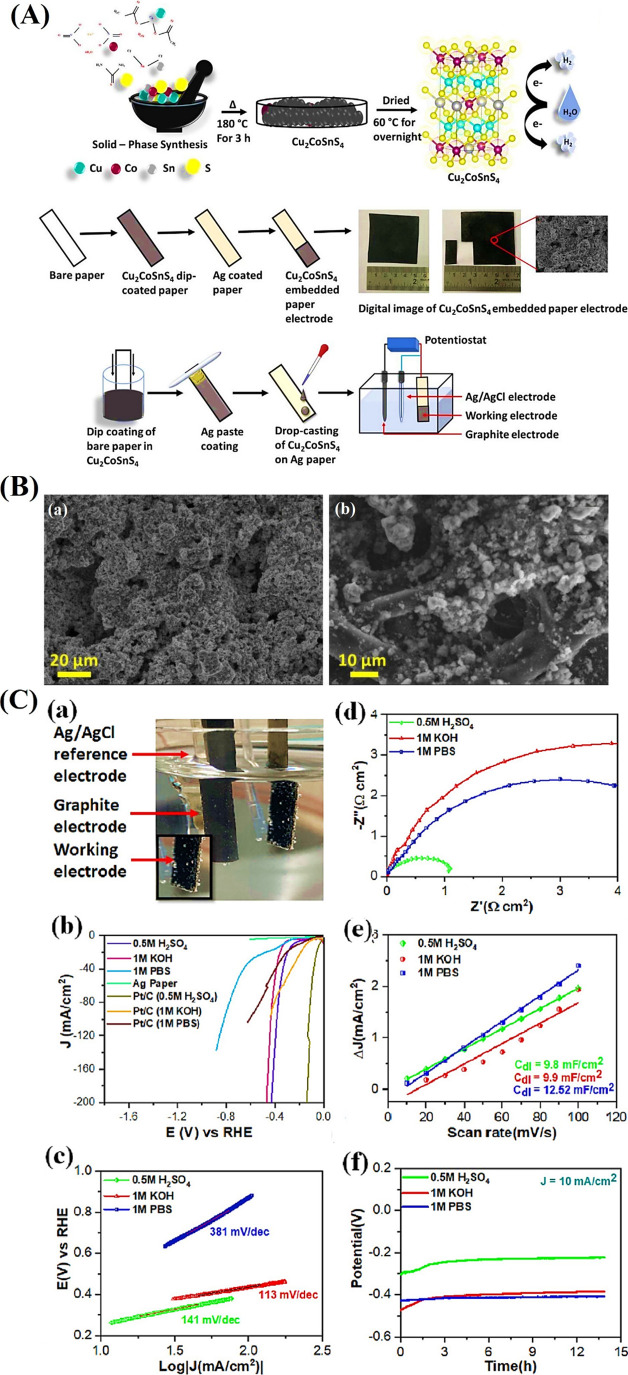
(A) Schematic of the solid phase synthesis of Cu_2_CoSnS_4_ and fabrication of the Cu_2_CoSnS_4_ embedded paper working electrode. (B) (a), (b) SEM micrographs of the Cu_2_CoSnS_4_ embedded paper electrode [front side and back side, respectively]. (C) Electrochemical measurements of the Cu_2_CoSnS_4_ embedded paper working electrode under acidic, alkaline, and neutral media: (a) digital image of the three electrode system, (b) linear sweep voltammetry curve at 1 mV s^–1^ scan rate, (c) Tafel slope, (d) Nyquist plot, and (e) relationship between capacitive current density and different scan rates. (f) Chronopotentiometry at 10 mA cm^–2^ for ∼14 h.

Copyright 2024, Elsevier.^158^

With versatile compositions and sizes of active sites, transition metal chalcogenides face challenges, such as poor electronic transport and restricted availability of sites. Monotransition metal sulfides are proven to enhance the HER performance in an array of electrolytes to counter the above-mentioned limitations.^175,176^ In addition to the Ni–Co foam substrate, Wang’s group demonstrated the extraordinary electrocatalytic performance of the newly developed Mo–Ni–Co selenide within a multiphase three-dimensional architecture (MoSe_2_–NiSe_2_–CoSe_2_/PNCF). This manner of employing dielectric barrier discharge (DBD) plasmas under atmospheric conditions for the HER in alkaline electrolytes makes the performance more pertinent, and a summarized illustration of the synthesis methodology is illustrated in Figure 19A. The regular MoSe_2_–NiSe_2_–CoSe_2_ nanorod arrays are formed on the plasma-treated NCF (PNCF) surface, which greatly increases the reactive area of the catalyst. First-principles DFT calculations (VASP) (see Figure 19B) have demonstrated that the CoSe_2_ core, combined with NiSe_2_–MoSe_2_ shell heterointerfaces, improves the intrinsic electronic properties of the MoSe_2_–NiSe_2_–CoSe_2_/PNCF structure. Low electrocatalytic resistance is consistent with the metallic nature, which is confirmed by the DOS close to the Fermi level. Additional electrons from the Ni 2p and Mo 3d states cause the NiSe_2_@CoSe_2_ and MoSe_2_@NiSe_2_ interfaces to move the Co d-band and main peak to lower energy, boosting the Fermi level. The computed Δ*G*_H_* values approach the optimal 0 eV, showing a lower energy barrier for H^+^ adsorption in HER, improving from −0.97 eV (CoSe_2_) to −0.58 eV (CoSe_2_@NiSe_2_@MoSe_2_). The optimized MoSe_2_–NiSe_2_–CoSe_2_ nanorods demonstrate excellent HER performance, exhibiting an overpotential of 38 mV at a current density of 10 mA cm^–2^. This performance exceeds that of single-phase MoSe_2_ catalysts, particularly in acidic electrolytes, due to the synergistic effects among the NiSe_2_, CoSe_2_, and MoSe_2_ phases. The Tafel slope for MoSe_2_–NiSe_2_–CoSe_2_/PNCF is measured as 38 mV dec^–1^, suggesting a Tafel–Heyrovsky mechanism with enhanced kinetics in alkaline media. In contrast, the Tafel slope for MoSe_2_–NiSe_2_–CoSe_2_/NCF is 114 mV dec^–1^, indicating a process dominated by the Heyrovsky mechanism (Figure 19C).^159^

**Figure 19:**
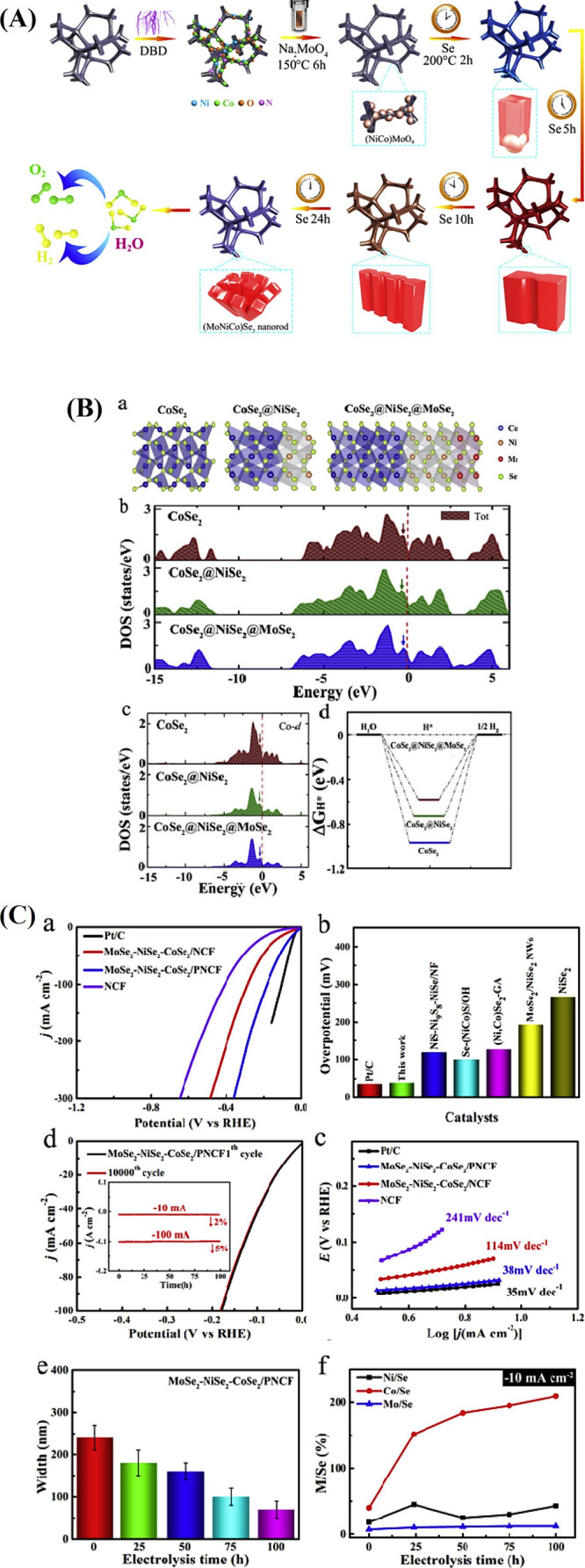
(A) The schematic fabricating processes of MoSe_2_–NiSe_2_–CoSe_2_ nanorods on the PNCF surface. (B) DFT calculations of CoSe_2_, CoSe_2_@NiSe_2_, and CoSe_2_@NiSe_2_@MoSe_2_ heterostructures. (a) Crystal structure of heterointerfaces, (b) calculated density of states (DOS), (c) *d*-band of Co atoms, and (d) HER free-energy evolution of different catalysts. (C) HER performances of the fabricated electrocatalysts in 1 M KOH. (a) LSV curves. (b) The overpotential comparison at a *j*_10_. (c) The effect of DBD plasma on the Tafel slopes of the fabricated catalysts. (d) Polarization curves of MoSe_2_–NiSe_2_–CoSe_2_/PNCF before and after 104 cycles, and the insets are the chronoamperometric curves of MoSe_2_–NiSe_2_–CoSe_2_/PNCF pushed with *j*_10_ and *j*_100_ for different times. (e) The width evolution of the nanorods with electrolysis time. (f) The M/Se ratio (M 14 Ni, Co, Mo) change with electrolysis time.

Copyright 2020, Elsevier.^159^

The catalytic performance of alloy electrocatalysts is highly influenced by their morphology. The bigger the surface area and smaller the diffusion length, the larger the improvement in mass and charge transport. N-doped hollow porous carbon spheres with CoCuFe have shown excellent efficiency and durability for HER.^160^ The ternary nanoparticle preparation is done through the template-assisted method using SiO_2_ as a hard template to synthesize hollow carbon spheres. P123 and polydopamine (PDA) are utilized in the synthesis process for the development of nanoshells to produce N-doped hollow porous carbon spheres with Co, Cu, and Fe alloy nanospheres (NHPCS@CoCuFe NSs). The process leads to well-defined and uniform structures. PDA deposits nitrogen and carbon, which fortify the carbon shell and facilitate core–shell nanostructure formation. P123 is a hole-swelling agent and hydrophobic surfactant that controls nanoparticle surface morphology. PDA facilitates Co^2^^+^ and Cu^2^^+^ adsorption via its catechol and amine groups (Figure 20A). The formation of the CoCuFe PBA composite via in situ conversion, wherein cobalt improves chemical dispersion and interactions of metals, and Cu2p facilitates nucleation while limiting overgrowth. Furthermore, due to the higher standard reduction potential of Cu to Co, the presence of Cu2p may impede the reduction of Co2p ions. The strong Lewis acidity of Fe3p lowers the activation energy of coordinated hydroxyl protons oxidation and heightens the defects density, further enhancing catalytic activity. The NHPCS@CoCuFe NSs obtained show high hardness, porous structure, and nitrogen doped, manifesting a Tafel slope of 73.72 mV dec^–1^ and overpotential of 158 mV (10 mA cm^–2^) (Figure 20B). The overpotentials for the N-HPCS@Co_1_Cu_2_ Fe NSs and N-HPCS@Co_2_Cu_1_Fe NSs achieve 166 mV and 109 mV, respectively, at a current density of 10 mA cm^–2^. The Tafel slopes of N-HPCS@Co_1_Cu_2_Fe NSs, N-HPCS@Co_2_Cu_1_Fe NSs, and Pt/C are 76.15, 98.42, and 65.77 mV dec^–1^, respectively.

**Figure 20:**
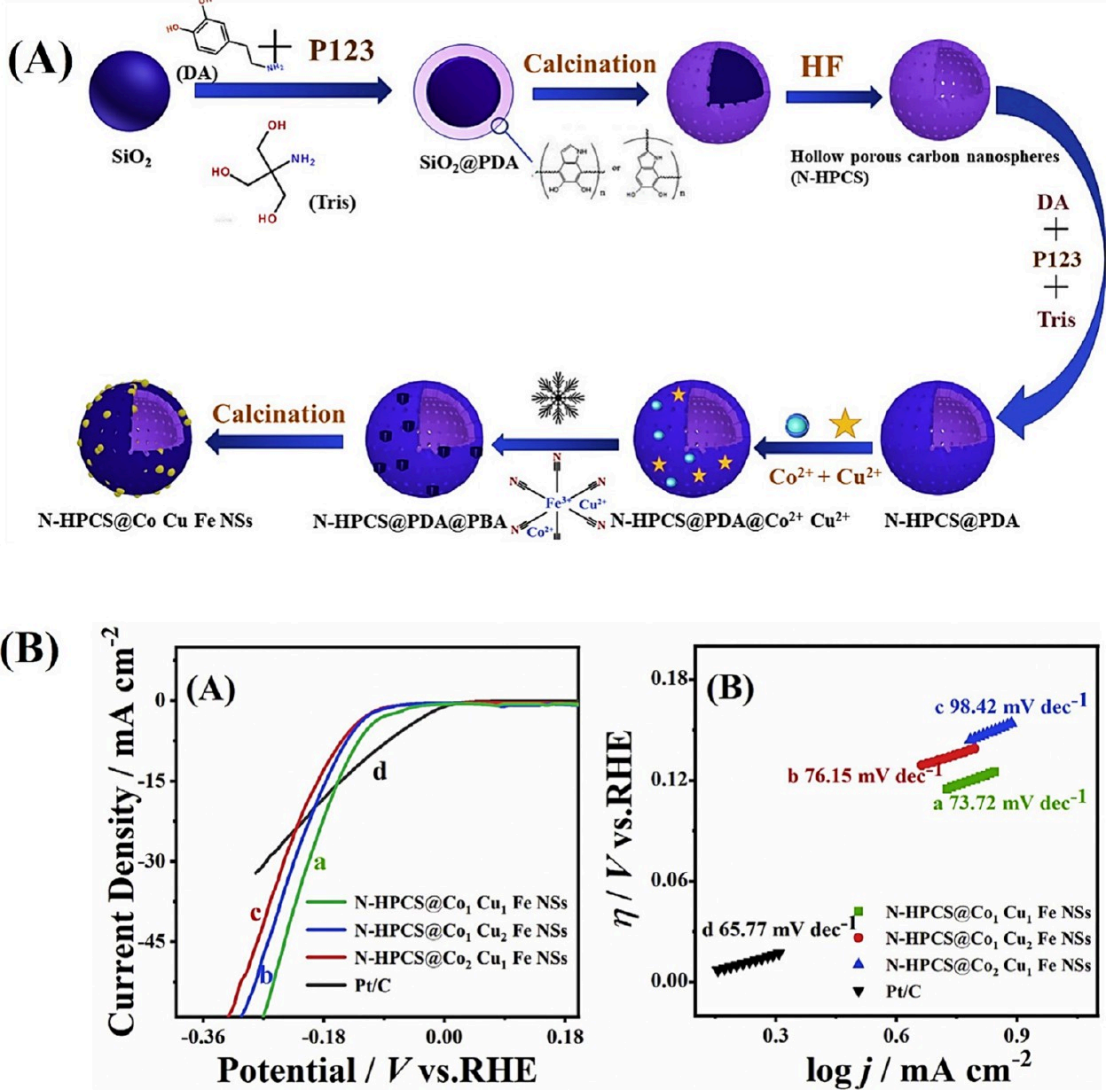
(A) The illustration of the preparation of trimetallic CoCuFe NSs loaded on N-doped hollow porous carbon nanospheres (N-HPCS@CoCuFe NSs). (B) (A) LSV curves of (a) N-HPCS@Co_1_Cu_1_Fe NSs, (b) N-HPCS@Co_1_Cu_2_Fe NSs, (c) N-HPCS@Co_2_Cu_1_Fe NSs, and (d) Pt/C for HER measured at a scan rate of 2 mV s^–1^ in 1 mol L^–1^ KOH. (B) Tafel plot.

Copyright 2022, Elsevier.^160^

MOFs are emerging as promising electrocatalysts owing to their complex pore structure and dispersed active sites, which favor the transport of electrons and ions for energy conversion. Their low conductivity and slow diffusion hindered their application in catalysis and energy storage in HER. Porous carbon materials are a solution to these concerns as they possess high electronic conductivity and offer synergistic core–shell effects to boost the mass transfer and surface polarity of the composite. The use of binary or ternary alloys as the catalytic material can produce more effective MOFs as the composition can modify the adsorption energies and lead to the production of redox active sites, which aids in optimizing the charge transfer and performance. Such a unique strategy not only contributes to strengthened charge transfer efficiency in an enriched fashion but also presents an advanced way for optimizing catalytic activity. Transition metal-based compounds from the first row (nickel, manganese, cobalt, etc.) stand out as an attractive avenue of material design for next-generation use. Nickel-centered electrode materials represent an advantageous option among multiple redox-active species owing to their variable chemical valence states, lower cost, and outstanding catalytic activity, making them a promising candidate for advanced electrochemical applications. Nickel-based compounds often have lower capacity for charge storage as semiconductors or insulators, which hampers their application in high-rate electrochemical reactions. On the other hand, cobalt-based electrode materials hold great potential for efficient charge storage in alkaline electrolyte and combine high specific capacitance with fast kinetics.

Construction of Ni-based MOF is achieved through a facile synthetic route, resulting in the fabrication of novel Ni_3_S_2_/MoS_2_/Fe_3_S_4_@NF heterostructure catalysts from MIL-53 (FeNi) nanosheets growing on nickel foam substrates, which exhibits excellent performance for HER. A high-level solvothermal approach has been utilized to design MIL-53 (FeNi) nanosheets on the nickel foam substrate via the synergistic coordination of organic ligand (TPA) with Fe^3+^ and Ni^2+^ metal centers. At the same time, the initial MIL-53 (FeNi) nanosheets were uniformly dispersed on a nickel foam substrate. Thioacetamide was used as a sulfur source during sulfidation under controlled hydrothermal conditions at 180 °C, yet this resulted in a partial conversion process giving birth to the development of rod-shaped structures comprising Ni_3_S_2_/MoS_2_/Fe_3_S_4_@NF-180. On the contrary, during this growth process, MoS_2_ tended to produce clustered blocks at the surface, which was undesired on the formation of independent ultrathin MoS_2_ nanosheets. As a post-treatment of Ni_3_S_2_/MoS_2_/Fe_3_S_4_@NF-220, the product evolved into a uniform flower shape with high surface area ultrathin MoS_2_ nanosheets. The surface morphology of the Ni_3_S_2_/MoS_2_/Fe_3_S_4_@NF-260 was significantly changed from the initial MIL-53 precursor at 260 °C, and irregularly assembled spheres of ultrathin MoS_2_ nanosheet were formed on the surface of the material, resulting in a great decrease of ultrathin MoS_2_ nanosheets over the material surface. These findings indicate that the hierarchical structure and morphology of the nanoassembly by various solvothermal and sulfidation processes can be successfully controlled by modifying the reaction temperature, with the product at 220 °C exhibiting highly hierarchical structures and the very best distribution of MoS_2_ sheets to mitigate the problems of aggregations. This structural optimization resulted in a notable increase in the electrochemical active surface area, enabling better access to active sites in the material (Figure 21A and B). Following sulfidation and MoS_2_ loading, the poor HER activity of MIL-53 (150 mV) significantly improved. The best performance (η_10_ = 54 mV) was demonstrated by Ni_3_S_2_/MoS_2_/Fe_3_S_4_@NF-220 because of the synergistic effect between MoS_2_ and Fe_3_S_4_, as well as its well-defined nanostructure, low aggregation, and large surface area. Its Tafel slope of 86.5 mV dec^–1^ is lower than those of Ni_3_S_2_/MoS_2_/Fe_3_S_4_@NF-260 (158.2 mV dec^–1^), Ni_3_S_2_/MoS_2_/Fe_3_S_4_@NF-180 (125.4 mV dec^–1^), and MIL-53 (129.5 mV dec^–1^), indicating enhanced HER kinetics and catalytic activity (Figure 21C).^161^

**Figure 21:**
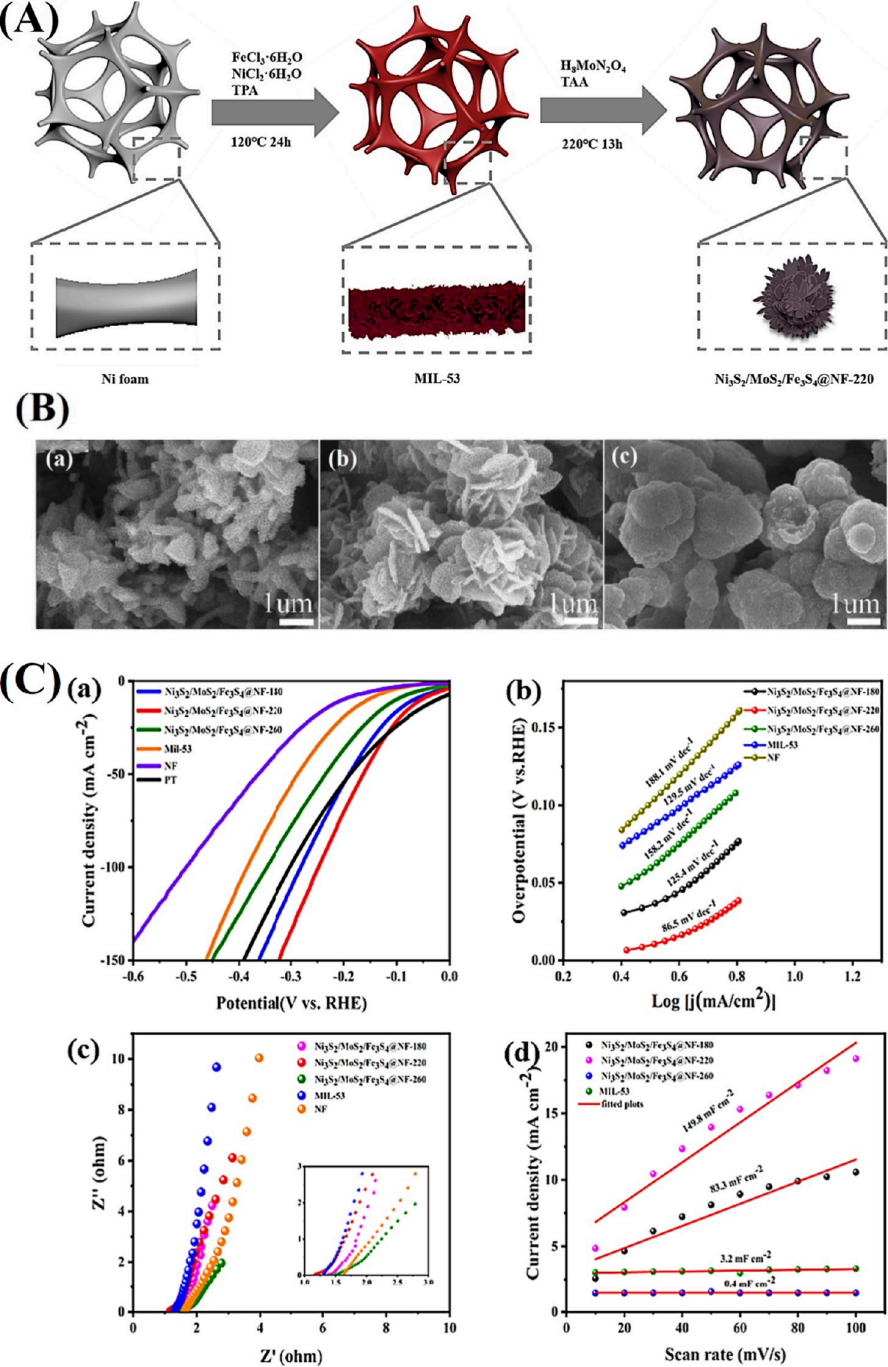
(A) Schematic illustration of the synthesis procedure of the Ni_3_S_2_/MoS_2_/Fe_3_S_4_@NF-220 electrode. (B) SEM images of (a) Ni_3_S_2_/MoS_2_/Fe_3_S_4_@NF-180, (b) Ni_3_S_2_/MoS_2_/Fe_3_S_4_@NF-220, and (c) Ni_3_S_2_/MoS_2_/Fe_3_S_4_@NF-260. (C) HER performance: (a) LSV curves, (b) Tafel plots, (c) EIS spectra, and (d) CV plots of current density as a function of scan rate.

Copyright 2023, Elsevier.^161^

By leveraging such synergistic effects, ternary metal sulfides show excellent HER performances, further improving charge transfer efficiency beyond that of single sulfides. Direct synthesis of MOF arrays on conductive substrates such as nickel foam, graphene, and carbon fiber paper does not rely on polymer binders to attach active materials to electrodes, thereby lowering internal resistance for enhanced charge transfer. The high conductivity and 3D porosity of nickel foam are well-suited for the growth of MOF arrays and for enhancing HER through electrocatalysis. For instance, Zhang et al. employed ZIF-67 as a precursor to prepare CoMxP/carbon cloths without binders, thus improving their HER activity by precisely doping with Fe, Mn, and Ni.^177^ In another approach, Liu et al. developed an original two-step solvent-thermal method to introduce the Fe dopant in situ and simultaneously create sulfur vacancies in bimetallic nickel–cobalt sulfide composites (NiCo-S) obtained from MOFs.^178^

The combination of Co, Ni, and Mn effectively contributes to generating active redox sites and increasing electrical conduction through the trinary CoNi_3_Mn_1_–OH in the structure of the ZIF-67 template, resulting in a highly synergistic interaction.^162^ A two-step approach was taken to construct this novel self-supported catalyst on carbon cloth (CC) with the assistance of ZIF-67 as a template. Initially, ZIF-67 was synthesized on CC, which played the role of a structural template and a cobalt precursor. Subsequently, the ZIF-67/CC was used not only as a template but also as a Co source for subsequent reactions under hydrothermal treatment at 90 °C, which facilitated the substitution of 2-methylimidazole with Ni^2+^ and Mn^2+^ ions, ultimately yielding the CoNi_3_Mn_1_–OH/CC composite (Figure 22A). The ZIF-67 nanosheets were carefully grown on carbon cloth with a leaf-like form distinguished by a smooth and uniform surface. The surface turned into wrinkled ultrathin nanosheets after reacting with Ni^2+^ and Mn^2+^, therefore boosting active sites and surface area. The obtained CoNi_3_Mn_1_–OH/CC hybrid exhibiting folded and woven oxide nanosheets on CC enables the formation of horizontal channels to facilitate electrochemical reactions. When the Ni/Mn ratio falls below 3:1, the material’s nanosheet growth becomes irregular and its leaf-like morphology is destroyed. This leads to a proportional decrease in ultrathin nanosheets in the overall morphology, which lowers the material’s active sites and compromises its electrochemical performance. Furthermore, CoMn–OH/CC does not exhibit volcano-like clusters covering the carbon rods’ surface in the absence of Ni, suggesting that Ni can preserve the ZIF-67 template’s shape. CoNi_3_Mn_1_–OH/CC demonstrates impressive HER activity in 1 mol L^–1^ KOH solution, achieving an overpotential of 202 mV at a current density of 10 mA cm^–2^. This performance surpasses that of Co–OH/CC (328 mV), CoNi–OH/CC (250 mV), and CoMn–OH/CC (279 mV), as illustrated in Figure 22B. The enhanced performance can be attributed to its heterogeneous interfacial nanostructure and the synergistic effects of the metal ions. This is further supported by the lowest Tafel slope of 212 mV dec^–1^, which indicates the fastest Volmer–Tafel kinetics when compared to CoNi–OH/CC (289 mV dec^–1^), CoMn–OH/CC (230 mV dec^–1^), and Co–OH/CC (413 mV dec^–1^), establishing it as the most effective catalyst among those tested. DFT calculations confirm M^2+^ oxidation in alkaline solution, with the doping of Mn and Ni resulting in a downward shift of the Co d states (Figure 22C), increasing OH adsorption. The OH molecule exhibits stronger binding to Ni and Mn sites, as reflected by a more negative *E*_ads_(OH) value compared to Co, though the *E*_ads_ difference (<0.07 eV) reveals comparable adsorption ability, contributing to a variety of reactive sites for redox reactions.

**Figure 22:**
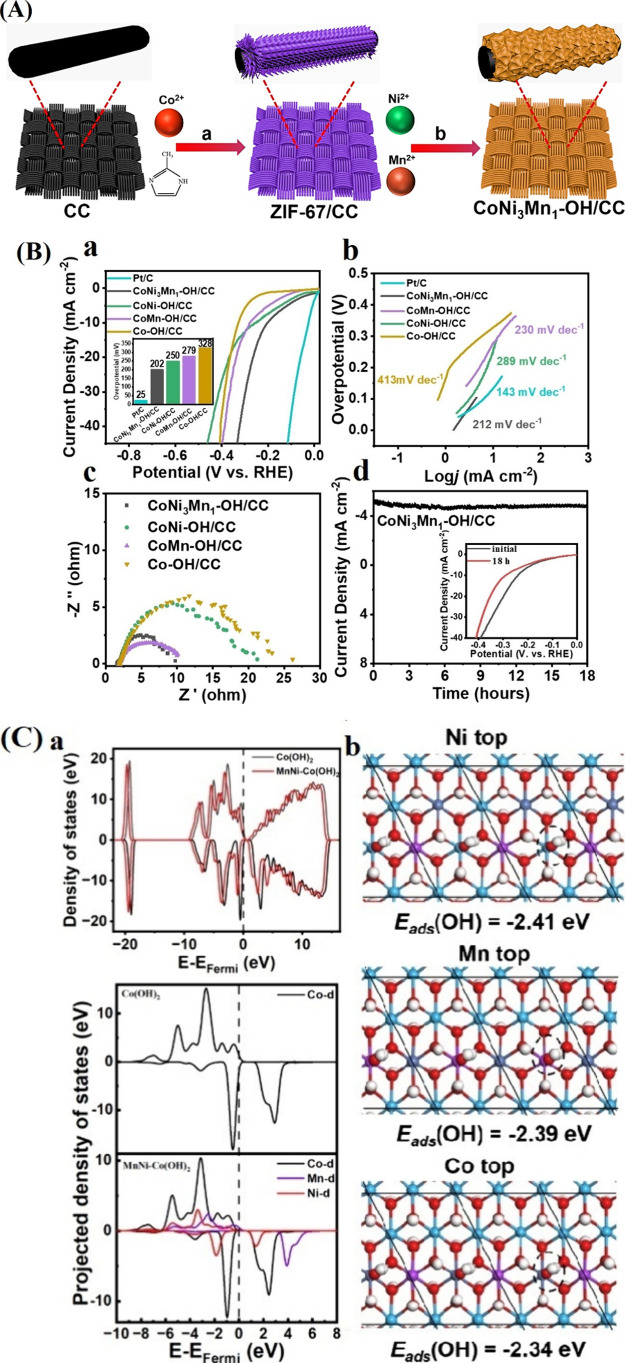
(A) Schematic diagrams for the synthesis of (a) ZIF-67/CC and (b) CoNi3Mn1-OH/CC. (B) (a) LSV curves and (b) corresponding Tafel plots of CoNi3Mn1-OH/CC, CoNi-OH/CC, CoMn–OH/CC, Co–OH/CC, and Pt/C for HER measured at a scan rate of 1 mV s^–1^ in 1 mol L^–1^ KOH. (c) Nyquist plots of CoNi_3_Mn_1_ OH/CC, CoNi–OH/CC, CoMn–OH/CC, Co–OH/CC, and Pt/C. (d) Chronoamperometric stability tests. Inset: LSV curves before and after the stability test. (C) (a) Total (up) and projected (down) density of states. (b) The most stable OH adsorption configurations and energies on MnNi-Co­(OH)_2_.

Copyright 2024, Elsevier.^162^

The investigation showcased that the incorporation of Ag and Cu with Au significantly enhances hydrogen evolution, performing comparably to Pt/C electrocatalysts. Key to energetic and catalytic pathways is the facile cleavage of reactants to yield reaction intermediates and the spontaneous accumulation of these species. The Ag edges facilitate the water splitting and the formation of hydrogen intermediates, which are effectively adsorbed near the Au surface for recombination into diatomic hydrogen.^163^ Significantly, including Cu lowers the energy barrier for hydrogen intermediate formation and desorption, therefore enhancing electron transfer kinetics. The electronic configuration of the Au-enriched surface inside the trimetallic AuAgCu alloy serves a dual role in slowing down transition metal dissolution as well as promoting additional surface properties. This special feature possibly explains the improved HER activity noted in the material. Given the much strengthened electron coupling interactions between Au, Ag, and Cu, the lower Au 4f binding energies can be blamed on poor oxygen binding affinity, which emphasizes the case. The complex dynamics inside the inner boundaries of the trimetallic alloy enhances the recovery rate of Au sites, therefore possibly increasing the electrochemical performance of the material for hydrogen evolution reaction.

First-row transition metal oxides offer great potential for environmentally friendly hydrogen generation. Outperforming the bimetallic CuO/Ag/SiNPs (BMSiNPs), the trimetallic CuO/Ag/NiO nanoporous material supported by silica nanoparticles (TMSiNPs) exhibits excellent HER catalytic properties. The silver layer is a key mediator to achieve a fast charge transfer between the CuO and NiO layers in the overall electrocatalytic system. Most prominently, NiO serves as the initial catalytically active layer directly interacting with hydrogen atoms to drive the hydrogen evolution reaction (HER), underscoring its critical role in the electrocatalytic mechanism of the trimetallic system. In the first sweep, Cu­(OH)_2_ and Ni­(OH)_2_ are formed and deposited on the surface of the CuO/Ag/NiO–GCE and is subsequently oxidized into CuO­(OH) and NiO­(OH) in alkaline media. The enhanced efficiency can be mainly attributed to the effective separation of charge carriers and the increase in active surface area. In the sol–gel procedure for preparing trimetallic CuO/Ag/NiONPs, the surfactant Brij S 20 presents a hydrophobic hydrocarbon tail as well as a hydrophilic polyethylene glycol head, which stabilizes the NPs prevents their aggregation and facilitates their arrangement into a nanoporous structure (Figure 23A). The inclusion of this NiO into surfactant doped TMSiNPs resulted in a significantly increased porosity of the material. Furthermore, acting as a pore-forming agent upon calcination, the addition of Brij S 20 synergistically interacts with NiO to greatly enhance the pore-forming capability of the material (Figure 23B and C). The presence of NiO nanoparticles is a crucial control of the porosity of the end product during sol–gel synthesis. Rising temperature breaks down the surfactant molecules into carbon compounds. Later, at high temperatures, these remnants combust and thereby produce holes inside in the nanoscale. The TMSiNPs-GCE had a lower overpotential of −1 V at a current density of 10 mA cm^–2^ than did BMSiNPs-GCE, given their Tafel slopes of 270 mV dec^–1^ and 294 mV dec^–1^, respectively (Figure 23D).^164^ The enhanced electrocatalytic activity of the CuO/Ag/NiO–GCE electrode manifests its high surface area and concentration of active sites that can facilitate rapid electron transfer and efficient electrocatalytic processes, which will be promising for sustainable hydrogen generation.

**Figure 23:**
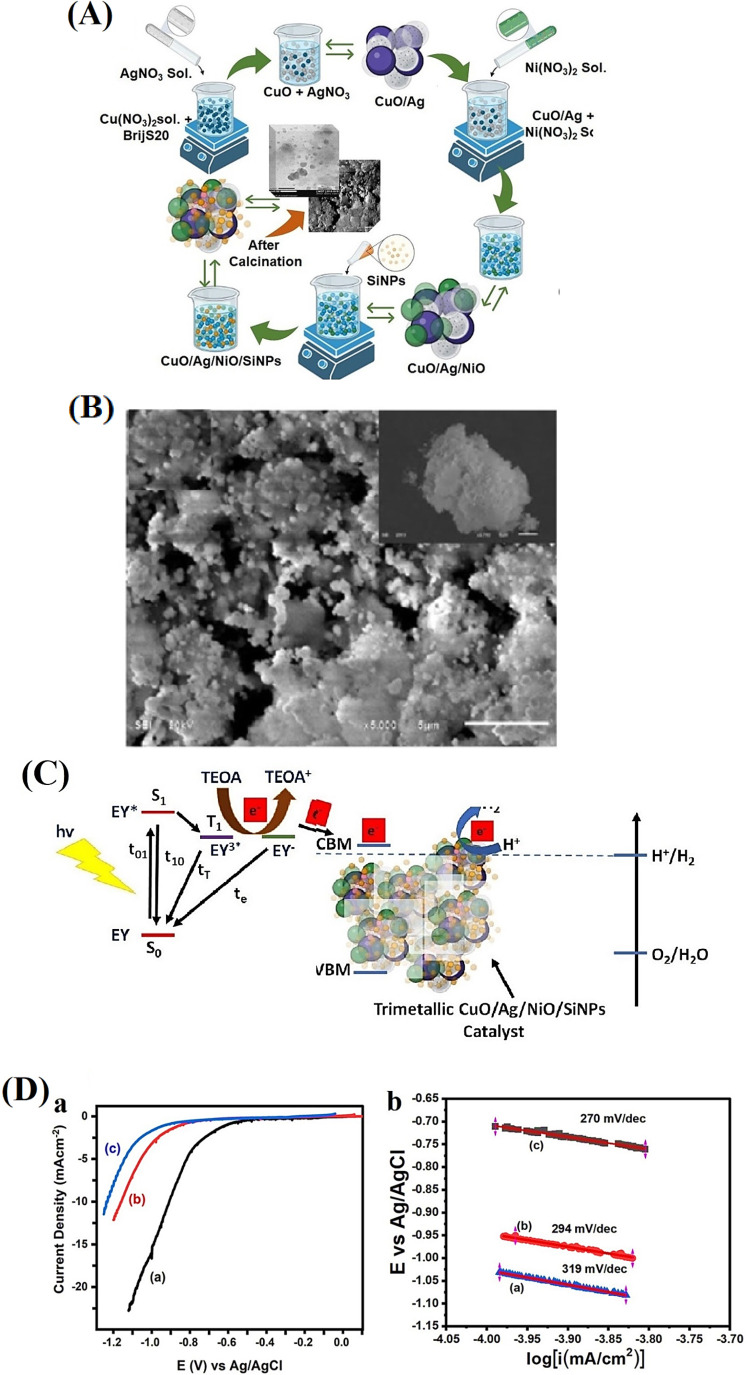
(A) Synthesis representation of CuO/Ag/NiO. (B) SEM image of CuO/Ag/NiO. (C) Evolution reactivity schematic representation using a CuO/Ag/NiO photocatalyst. (D) (a) LSV comparison of (a) CuO/Ag/NiO-GCE, (b) CuO/Ag-GCE, and (c) bare-GCE. (b) Tafel plots were constructed based on the polarization curves obtained in (a) bare-GCE, (b) CuO/Ag, and (c) CuO/Ag/NiO.

Copyright 2023. Nature.^164^

Silva et al. synthesized CoNiMn oxide via electrodeposition on FTO, followed by electrochemical activation, to enhance OER performance. The trimetallic CoNiMn film outperforms monometallic and bimetallic oxides, achieving overpotentials of 100 mV at 10 mA cm^–2^ and 430 mV at 25 mA cm^–2^, with a Tafel slope of 58 mV dec^–1^.^179^ Synergy between hydrogenation-active metal sites (Ni, Co) and oxophilic sites (Fe, Co, Mn, Sn) achieves maximum adsorption and catalytic activities. Particularly, nickel and cobalt exhibit high activity toward hydrogen adsorption on isopropanol to form active hydrogen species. However, nonprecious metal catalyst systems often suffer from drawbacks such as long reaction times and high temperatures, and they require molecular hydrogen as a reactant and, hence, require cost-effective catalysts with mild-operating conditions. In addition, the strong interaction of hydroxyl oxygen of the hydrogen donor with the strong acidic sites is beneficial to this process, indicating that the strong interaction can enhance the catalytic ability of promoting hydrogen extraction. Introducing zirconium in NiZr_1_/CoO_*x*_-400 suppresses parallel adsorption of the fluorine functionalities, hinders the evolution of crystals, and promotes defect generation, as well as enhancing the specific area of the catalyst. Ni^0^ breaks the hydrogen bonds of isopropanol, while the oxide vacancy of NiZr/CoO_*x*_ favors fluoride adsorption on the cobalt site. Oxygen vacancies and floor fee versions enhance aldehyde/ketone adsorption, reducing hydrogenation barriers. Moreover, through modulation of the Zr ratio in NiZr/CoO_*x*_ catalysts and adjustment of the temperature, the oxygen floor traits of the catalyst may be finely tuned, providing a clean method to the planned layout of selective hydrogenation catalysts.

To enhance hydrogen evolution efficiency in NiFe-based catalysts for near-complete water splitting, recent advancements focus on multicomponent nanoarray catalysts that increase active sites, conductivity, and charge transfer.^180^ Multicomponent, multi-interface nanoarray catalysts like NiCo_2_O_4_@NiFe-LDH^181^ and Pt-loaded NiFe-LDH frameworks^165^ make use of 2D materials with rich positive charges and active sites to achieve efficient *OH adsorption for driving reaction forward. Transition metal oxides are particularly favorable for facilitating H–OH bond dissociation because hydroxyl radicals (*OH) adsorb preferably on the surfaces of metal oxides. This is attributed to the powerful electrostatic attraction between *OH and positively charged metal species at the surface of the oxides. According to the above knowledge, strategic coupling of NiFe-LDH with transition metal oxides is a potential direction toward enhancing the efficiency of water electrolysis in alkaline conditions. This synergistic approach capitalizes on the catalytic prowess of both materials, harnessing their unique surface properties to accelerate the electrochemical splitting of water into hydrogen and oxygen. For example, the interaction between Co_3_O_4_ nanowires and NiFe-LDH nanosheets improves ionic and electronic transport, enhancing electrolyte ion access to active sites. A flower-shaped Co_3_O_4_@NiFe-LDH composite catalyst was created on a nickel foam substrate through a three-step synthesis process, as presented schematically in Figure 24A. The Co_3_O_4_ precipitation was triggered through the reaction of cobalt nitrate, urea, and NH_4_F, yielding cobaltate hydroxide precursor ions. Due to its inherent hydrophilicity, Co_3_O_4_ serves as the seeding substrate for immobilization of Fe^3+^ and Ni^2+^ ions. The procedure enhances the even distribution of such ions on the Co_3_O_4_ surface for subsequent hydrolysis to obtain well-defined NiFe-LDH nanosheets. The nanostructured fibrous substrate-supported Co_3_O_4_@NiFe-LDH composite forms a hierarchical 3D structure with built-in conductivity without binders and enhancing water electrolysis efficiency. The structure enhances gas bubble evolution, optimizing water electrolysis. The Co_3_O_4_@NiFe-LDH composite outperforms individual materials in electrocatalytic activity because of its high-speed electron transfer channel and new 3D core–shell structure. The HER activity of Co_3_O_4_@NiFe-LDH-120/NF (η_10_ = 213 mV) is not lower than that of Pt/C/NF (η_10_ = 79 mV), yet it surpasses the performance of Co_3_O_4_/NF (226 mV) and NiFe-LDH (254 mV). The optimal performance is observed at a treatment temperature of 120 °C, with Co_3_O_4_@NiFe-LDH-100/NF showing lower overpotentials of 140 mV and Co_3_O_4_@NiFe-LDH-140/NF exhibiting even lower overpotentials of 99 mV (Figure 24B). The nanoheterostructure formed between Co_3_O_4_ and NiFe-LDH demonstrates enhanced activity, exhibiting a Tafel slope of 100.1 mV dec^–1^ for Co_3_O_4_@NiFe-LDH-120/NF, in contrast to 162.8 mV dec^–1^ for Co_3_O_4_/NF and 129.8 mV dec^–1^ for NiFe-LDH/NF. This shift indicates a transition of the rate-limiting step from the Volmer to the Heyrovsky mechanism under alkaline conditions. This enhancement, attributed to the electron transport channels of Co_3_O_4_ and the formation of H_ads_ by NiFe-LDH, underscores the synergistic effects and the importance of interface engineering in improving HER kinetics.^166^

**Figure 24:**
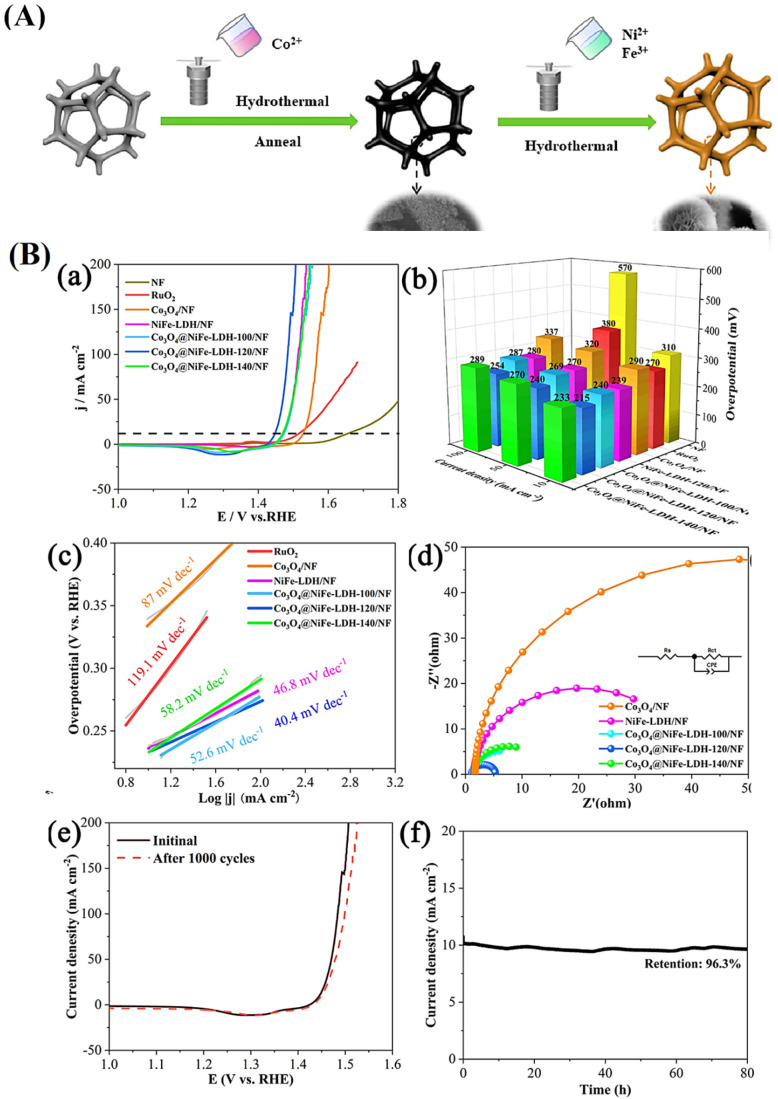
(A) Schematic illustration of the synthesis route for Co_3_O_4_@NiFe-LDH/NF. (B) (a) HER polarization curves. (b) The overpotential values at different current densities, (c) Tafel slopes, and (d) EIS Nyquist plots at an open circuit potential for different electrocatalysts. (e) The polarization curves before and after 1000 cycles and (f) chronoamperometry (*i*–*t*) curve of Co_3_O_4_@NiFe-LDH-120/NF under a constant voltage of −1.302 V for HER.

Copyright 2022, Elsevier.^166^

## 5 Recent Progress of Nanostructured Trimetallic Electrocatalysts toward Water Splitting

### 5.1 Multifunctional Catalysts

Many existing catalysts are optimized for only one of the two half-reactions involved in water splitting, highlighting the urgent need to investigate multifunctional catalysts exhibiting noble metal-like activity, cost-effectiveness, and exceptional durability to facilitate the overall water-splitting process. Consequently, the quest for environmentally benign and relatively abundant transition metal electrocatalysts is crucial to ensure prolonged stability and intrinsic electrocatalytic efficiency.

In developing water electrolyzers, the great challenge that remains is the application of different electrocatalysts in an integrated electrolyzer, posing difficulties because of different electrolyte pHs and high costs of production. Consequently, the search for nonprecious, highly efficient multifunctional electrocatalysts remains a key focus in scientific research. Table 3 summarizes the performance toward HER and OER for trimetallic electrocatalysts.

**Table 3:** OER/HER Performance of Trimetallic Electrocatalysts in Alkaline Media (1 mol L^–1^)

catalysts	substrate	(mA cm^–2^)	η_OER_ and η_HER_ (mV)	Tafel slope (mV dec ^–1^)	ref
Fe_2_O_3_/ZnCo_2_O_4_	GC^a^	10	44.8 HER	38.9	^182^
			212 OER		
(FeMnCe)-*co*-doped MOF-74	NF^b^	10 HER	186 HER	98.7 HER	^83^
		100 OER	281 OER	41.9 OER	
SC-FeNiCeP	NF	10	107 HER	24.7 OER	^183^
			208 OER	41.54 HER	
Fe_2_V-MOF	NF	10	198 HER	58 OER	^184^
			314 OER	174 HER	
Ni–Fe–Mn–P/NC	NF	10 HER	72 HER	79.8 HER	^185^
		30 OER	274 OER	56.8 OER	
CoFeNi-MOG	NF	10	147 HER	77 HER	^134^
			267 OER	23.6 OER	
CuCo_2_S_4_@CoFe-LDH	NF	10 HER	63 HER	59 HER	^186^
		50 OER	293 OER	73 OER	
CuNiCoS4/1TMoS_2_	NF	10 HER	160 HER	92 HER	^187^
		50 OER	163 OER	53.5 OER	
Ni–Co–Fe-Se@NiCo-LDH	NF	10 HER	113 HER	44.87 HER	^188^
		100 OER	286 OER	78.9 OER	
CoNiCu-LDH@CuO	CF	100	262 HER	88.4 HER	^189^
			286 OER	70.6 OER	
NiCoFeLDH/NiCoFeS	NF	100	191 HER	90.54 HER	^143^
			241 OER	31.51 OER	
NiFeVSx@NF	NF	10	127 HER	121	^190^
			259 OER		

a Glassy carbon electrode.

b Nickel foam.

To elevate the catalytic efficacy of monometallic transition metal (TM) catalysts, two fundamental strategies are typically implemented. First, regulating morphology and nanostructural features optimizes the exposure of catalytically active sites.^191^ Defects introduced by ion substitution or doping can tune the electronic structure to enhance catalytic activity.^192^ Engineered nanomaterials undergo various structural manipulations, such as dopant introduction, integration with supporting substrates, alloy formation, and application of advanced methodologies to improve their functional performance.^193,194^

Substantive efforts have been devoted to utilizing oxides, hydroxides, phosphides, nitrides, chalcogenides, alloys, and composites to develop non-noble metal electrocatalysts for water splitting. Hydroxides, oxides,^195,196^ sulfides,^197,198^ phosphides,^199^ and selenides^200^ of first-row transition metals (Cu, Cr, Zn, Fe, V, Co, Ni, Mn, Ti) have been extensively modified to exhibit high activity as both HER and OER catalysts. Unlike in noble metal-based counterparts, these materials are preferred because they are accessible, inexpensive, easy to produce, and in abundance. Cobalt, iron, and nickel have been especially notable candidates in such a field.^201^

Bimetallic catalysts have been widely studied because they exhibit high intrinsic reactivity, selectivity, and stability and have provided important guidance for future directions in research. Dual-functionalization in single-component catalysts, however, is difficult to accomplish due to the difference in intrinsic activity between HER and OER. An efficient electrocatalyst for HER does not necessarily exhibit comparable activity for OER, and vice versa. To overcome such a limitation, composite catalysts have been developed as a suitable alternative, capitalizing on advantageous interfaces to promote active species adsorption and increase charge transfer efficiency between different components. Such advances underscore composite materials’ ability to overcome limitations of single-component catalysts.

MOF-derived electrocatalysts based on transition metal precursors and organic linkers are exceptionally porous, amenable to structural adaptation, and allow for anisotropic composition, improving the availability of active sites and their cost-effective electrochemical behavior. The monometallic layered double hydroxides inherited catalytic activity in water splitting but had their performance insufficient due to structural instability and low electronic conductivity. Hybrid LDH-based nanostructures use three-dimensional architectures to overcome these shortcomings, which leads to the improvement of surface area, porosity, and availability of electroactive sites, thus substantially boosting electrocatalytic performance.

### 5.2 Engineering Trimetallic MOFs for Superior Electrocatalytic Performance

To achieve high electrocatalytic activity, MOF materials with high surface areas, accessible active sites, tunable porosity, and hybrid composition have been developed. MOFs, made from inorganic metal centers coordinated with organic ligands, exhibit different topologies, tunable porosity, and electrochemical properties with high versatility. These result from interactions between metal ions (Lewis acids) and organic ligands (Lewis bases) with oxygen or nitrogen donor groups. While being favorable in many aspects, pristine MOF-derived nanomaterials suffer from limitations in terms of aggregation-induced porosity loss and low conductivity. Addressing these limitations is vital for enhancing electrocatalytic efficiency. However, achieving targeted improvements without compromising other properties remains a significant challenge.

The incorporation of multiple metals into the catalyst improves the electronic configuration and coordination environment while boosting the adsorption energies, therewith making a solution to the conventional drawbacks. In addition, the coexistence of multiple metals with unequally sized ionic radii leads to a disturbance and defect formation in the crystal lattice, which can eventually impact the electrocatalytic activities. This combination within MOFs helps form interconnected networks, thus increasing their specific surface areas, porosities, and functional tunability beyond standard transition metal-based oxides, chalcogenides, or phosphides.

Significantly, sustainable hydrogen production is possible with seawater electrocatalysis. OER catalysts with overpotentials below 480 mV and strong corrosion resistance to chloride ions are necessary because the OER and the chlorine evolution process (CER) compete in seawater. The porous architecture and exposed active sites of MOFs and MOF-derived LDHs make them efficient electrocatalysts. The stability and activity of LDHs are improved by techniques such as anion intercalation and heterogeneous metal doping, which minimize Cl^–^ adsorption while maximizing charge transfer and intermediate adsorption/desorption. Because MOF/LDH hybrids, such as those made from ZIF, increase active sites and speed up electron transfer, they further enhance the electrocatalytic performance for effective saltwater hydrogen production. In order to improve stability and active site exposure, Zhao et al. created the NiCoFe LDH@ZIF-67 sheet-array (NiCoFe ZSA) on copper foam using SO_4_^2–^ interlayer ions. While synergistic Ni, Co, and Fe interactions as well as 2-methylimidazole enhance charge transfer and conductivity, Fe doping optimizes Ni and Co electronic structures, improving OER and HER activity. With Cl^–^ repulsion for stability and Fe and SO_4_^2–^ boosting corrosion resistance, the hydrophilic NiCoFe ZSA-30 guarantees effective reactant/product transport, attaining low overpotentials of 326 mV (OER) and 321 mV (HER) at 500 mA cm^–2^ in alkaline simulated seawater. Kinetics of surface and bulk diffusion are controlled, providing a reliable method for producing hydrogen from saltwater.^202^

Trimetallic MOFs (FeCoZn) were used to create multimetal oxide electrocatalysts for water splitting (see Figure 25A). ZIFs, a subclass of MOFs, serve as ideal precursors for creating polyhedral-shaped Fe_2_O_3_/ZnCo_2_O_4_ heterointerface catalysts, which exhibit a synergistic effect that enhances catalytic performance through increased accessible active sites, improved electrical conductivity, facilitated charge transfer, and increased adsorption free energy. Fe^2^^+^, Zn^2^^+^, and Co^2^^+^ precursors react with 2-methylimidazole to form FeZnCo-ZIFs via coprecipitation. These are then annealed in a tube furnace, transforming into Fe_2_O_3_/ZnCo_2_O_4_ porous polyhedron nanocomposites.^203^ Bimetallic spinel oxides, particularly ZnCo_2_O_4_, show superior water oxidation properties compared to individual metal oxides and offer benefits like chemical stability, abundant active sites, redox capabilities, and enhanced conductivity when combined with dual transition metal oxides. The poor conductivity of Fe_2_O_3_ may be lessened by the well-designed Fe_2_O_3_/ZnCo_2_O_4_ nanocomposite, which also provides a wide electrode/electrolyte contact surface. The Fe_2_O_3_/ZnCo_2_O_4_ nanocomposite, created via coprecipitation and annealing, exhibits a porous polyhedral morphology and heterointerfaces that support efficient HER and OER in alkaline environments, with notably low overpotentials of 31 mV and 212 mV at 10 mA cm^–2^, respectively. The HER activity for the Fe_2_O_3_/ZnCo_2_O_4_ nanocomposite with an ultralow overpotential of 31 mV is much lower than those of commercial Pt/C (η_10_ = 76 mV), ZnCo_2_O_4_ (η_10_ = 136 mV), and Fe_2_O_3_ (η_10_ = 253 mV). The synergistic interface between Fe_2_O_3_ and ZnCo_2_O_4_ results in a lower overpotential for the Fe_2_O_3_/ZnCo_2_O_4_ catalyst, which increases HER activity. The Volmer step (H* adsorption from H_2_O dissociation) and the Tafel or Heyrovsky stages for H_2_ production are the first of several phases in HER that depend on the electrical and chemical characteristics of the electrode. The polyhedron interface Fe_2_O_3_/ZnCo_2_O_4_ nanocomposite shows a lower Tafel slope value of approximately 38.9 mV dec^–1^. This is much lower than ZnCo_2_O_4_ (65.4 mV dec^–1^) and Fe_2_O_3_ (82.7 mV dec^–1^) but marginally lower than commercial Pt/C (41.2 mV dec^–1^) (Figure 25B). Regarding OER activity, the Fe_2_O_3_/ZnCo_2_O_4_ catalyst shows an overpotential of 212 mV at 10 mA cm^–2^, lower than RuO_2_ (245 mV), ZnCo_2_O_4_ (284 mV), and Fe_2_O_3_ (321 mV), with enhanced OER activity attributed to high-valent Fe species at the heterointerface. In comparison to RuO_2_ (56.2 mV dec^–1^), ZnCo_2_O_4_ (74.7 mV dec^–1^), and Fe_2_O_3_ (93.5 mV dec^–1^), its Tafel slope of 40.5 mV dec^–1^ validates improved OER kinetics, most likely as a result of multicomponent synergism improving adsorption-free energy (Figure 25C). At a low cell voltage of 1.44 V, the two-electrode water electrolyzer reaches a current density of 10 mA cm^–2^.^182^

**Figure 25:**
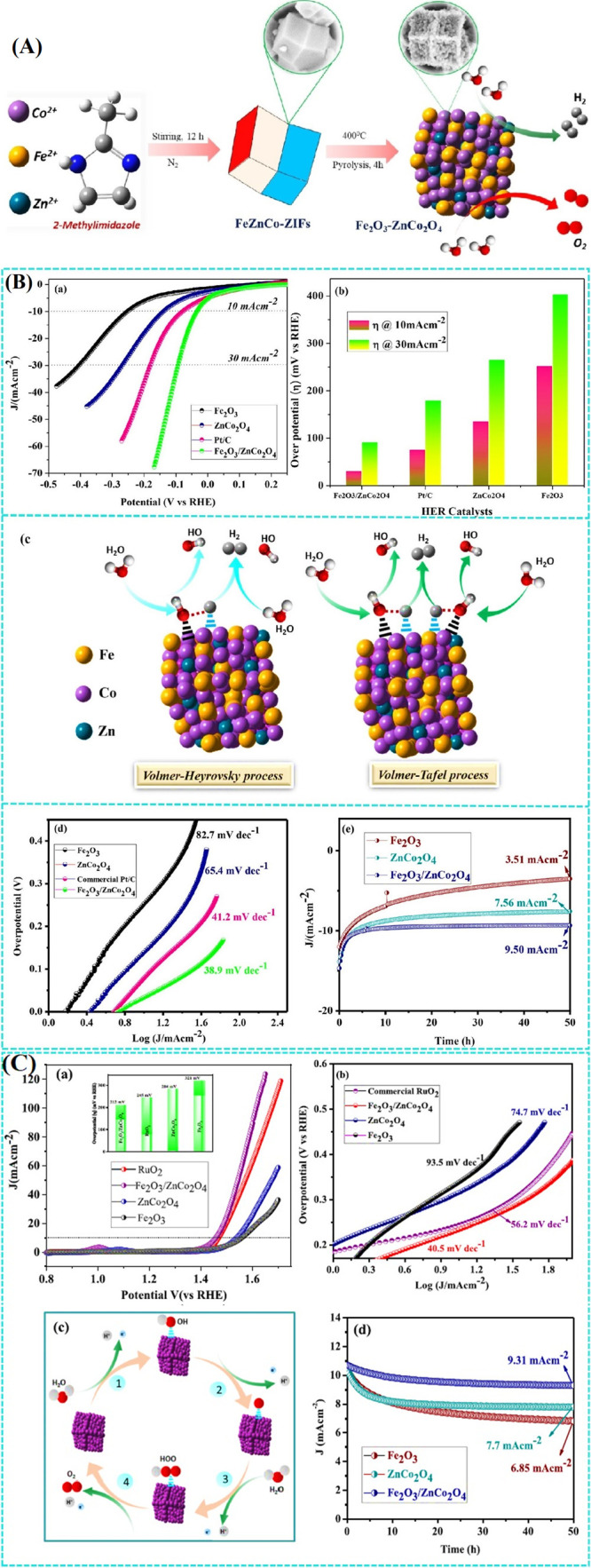
(A) Illustration of the synthesized interfacing Fe_2_O_3_–ZnCo_2_O_4_ polyhedron nanocomposite. (B) (a) HER LSV-polarization curves at 1 mol L^–1^ KOH electrolyte solution with a scan rate of 10 mV s^–1^. (b) Comparison of the overpotential (η) of the as prepared electrocatalysts and commercial Pt/C. (c) Possible HER mechanism over the Fe_2_O_3_/ZnCo_2_O_4_ interfacing nanocomposite. (d) Tafel plots of obtained samples and (e) HER current–time analysis by using a chronoamperometric (CA) to estimate the durability of the catalysts. (C) OER LSV-polarization curves at 1 mol L^–1^ KOH electrolyte solution with a scan rate of 10 mV s^–1^ (inset: overpotential (η) values of the as-prepared electrocatalysts and commercial RuO_2_). (b) Corresponding Tafel plots and (c) possible OER mechanism over the Fe_2_O_3_/ZnCo_2_O_4_ interfacing nanocomposite. (d) OER current–time analysis by using a chronoamperometric (CA) to estimate the durability of catalysts.

Copyright 2023, Elsevier,^182^

The MOF-74-type frameworks are distinguished in electrocatalysis due to their high density of accessible metal sites, hexagonal channels aligned along the *c*-axis, and tunable porosity. Various modification techniques, including ligand extension, have been employed to optimize their properties, resulting in enhanced surface area and catalytic activity.^204^ Bimetallic or polymetallic MOFs usually surpass monometallic catalysts due to synergistic interactions between different metal species, which enhances electron conductivity, diminishes charge transfer resistance, and improves intermediate adsorption. These interactions align with the hard–soft-acid–base (HSAB) theory, significantly boost electrochemical performance, and broaden the applicability of these materials. A typical example includes a trimetallic MOF-74, a 3d-4f FeMnCe-MOF-74, wherein Ce introduction enhances electronic conductivity and stability.^83^ (1) The distinctive 4f orbitals in rare earth elements such as Ce enable orbitals to overlap and hybridize with neighboring orbitals, and thus, electronic structure modulation becomes easy.^191^ (2) Interplay between d and f orbitals enables electronic ladders to be constructed, and thus, charge transfer mechanisms become easy and effective. (3) Materials with a combination of a 3d transition metal and a 4f rare earth metal have an oxygen vacancy-rich structure due to differences in radii and coordination and thus lead to lattice distortions. So, a 3d–4f mixed metal approach works well to boost the electrocatalytic performance of MOF based catalysts. The introduction of rare earth elements, especially Ce, in 3d transition metal-based MOF-74 structure improves the catalytic activity by modulating electronic structure, charge transfer, and oxygen vacancies. In this study, an elaborate iron, manganese, and cerium-based MOF-74 catalyst was prepared in situ by the hydrothermal method using nickel foam (NF) as a conductive substrate. The unusual properties of the 4f orbitals of Ce make this metal able to mix with adjacent orbitals, which in turn brings improvement to the electronic conductivity and the redox ability through the Ce^3+^/Ce^4+^ pair. Single metal-MOF-74 samples exhibit nanospherical morphology with agglomeration, according to SEM results. Bimetallic FeCe-MOF-74/NF, which is nanospherical, MnCe-MOF-74/NF, which is needle-shaped, and FeMn_6_-MOF-74/NF, which is nanoflowered, are produced when Ce is added to the synthesis (Figure 26A). Such a structural adjustment increases the accessibility of the active site and the electrolyte-electrode contact interface, thus greatly improving HER and OER activities. Furthermore, trimetallic FeMn_6_Ce_0.5_-MOF-74/NF shows the lowest overpotential of 281 mV at 100 mA cm^–2^, in comparison to the bimetallics catalysts FeCe_0.5_-MOF-74/NF (286 mV) and MnCe_0.5_-MOF-74/NF (355 mV) for OER. Tafel slopes demonstrate the efficacy of the 3d–4f mixed metal strategy through synergistic multimetal effects, confirming the fastest kinetics of FeMn_6_Ce_0.5_-MOF-74/NF (41.9 mV dec^–1^) in comparison to single (Fe-MOF-74/NF: 125.4 mV dec^–1^), bimetallic (FeCe_0.5_-MOF-74/NF: 79.9 mV dec^–1^), and other trimetallic samples. With an overpotential of 232 mV at 10 mA cm^–2^, Fe-MOF-74/NF exhibits the highest HER activity among the single metals, surpassing Mn-MOF-74/NF (257 mV) and Ce-MOF-74/NF (237 mV). While trimetallic FeMn_6_Ce_0.5_-MOF-74/NF performs better with 186 mV, surpassing FeMn_6_-MOF-74/NF (315 mV) and outperforming Pt/C (245 mV) at 50 mA cm^–2^ (228 mV), the bimetallics FeCe_0.5_-MOF-74/NF (163 mV) and Mn_6_Ce_0.5_-MOF-74/NF (231 mV) are improved upon single metals. The efficacy of the 3d–4f mixed metal strategy is demonstrated by the lowest Tafel slope of FeMn_6_Ce_0.5_-MOF-74/NF (98.7 mV dec^–1^), which validates the fastest HER kinetics (Figure 26B and C). As a bifunctional anode and cathode, FeMn_6_Ce_0.5_-MOF-74/NF needs just 1.65 V to reach a current density of 10 mA cm^–2^ for overall water splitting (OWS).

**Figure 26:**
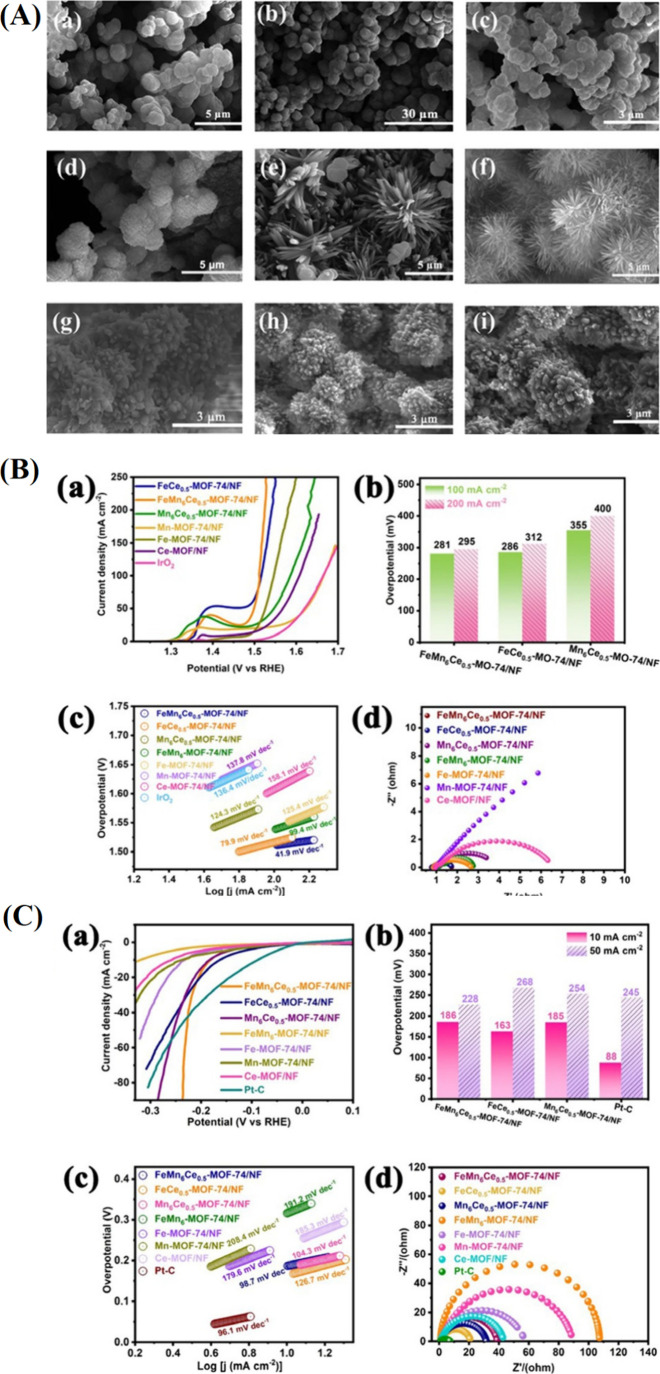
(A) SEM images of (a) Fe-MOF-74/NF, (b) Mn-MOF-74/NF, (c) Ce-MOF/NF, (d) FeCe_0.5_ MOF-74/NF, (e) Mn_6_Ce_0.5_-MOF 74/NF, (f) FeMn_6_-MOF-74/NF, (g) FeMn_6_Ce_0.5_-MOF-74/NF, (h) FeMn_6_Ce_1_-MOF-74/NF, and (i) FeMn_6_Ce_2_-MOF-74/NF. (B) OER and (C) HER: (a) LSV curves, (b) overpotentials, (c) Tafel plots, and (d) EIS spectra.

Copyright 2023, RSC.^83^

Transition metal phosphides (TMPs) exhibit tunable oxidation states akin to noble metals, facilitating charge transfer and intermediate adsorption through electron rearrangement at metal active centers. Redistribution of electrons by phosphorus at metal active centers also improves electrochemical activity. The electronegativity of phosphorus in TMPs promotes electron withdrawal from metal atoms, enabling M–P bond formation and optimization of the adsorption energy to balance hydrogen binding in the Volmer step, thereby mitigating catalyst poisoning. In addition, negatively charged P could also act as a proton acceptor in HER and optimum activity was found with adjusting P content. However, excessive P content can hinder electron delocalization, reducing metallic properties and overall conductivity. Despite their potential in water splitting, TMPs suffer from sluggish kinetics due to limited conductivity, insufficient active sites, and structural instability.^199^ Nevertheless, achieving the requisite scale for hydrogen production poses a significant challenge, chiefly concerning the limited accessibility of active sites and the inadequate electrical conductivity. In view of these challenges, MOF-based TMPs are adopted as they can utilize the intrinsic porosity and periodic nature of MOFs to prevent the agglomeration of active sites and improve electron–proton transport at the same time by introducing high-valency metals (e.g., Mo, Ce, Mn, V).^205^ High-valence metal ions (e.g., W^6^^+^, V^5^^+^, Mo^5^^+^, Ce^4^^+^) facilitate electron occupancy in vacant d orbitals, tuning bond length and energy to enhance catalytic and energy storage performance.^206–208^ Additionally, the carbon substrates derived from MOFs inhibit the aggregation of metal phosphides and speed up kinetics of the electron transfer process. Also, dopants (e.g., N, S, B) could be incorporated into the carbon matrix to enhance conductivity, surface wettability, or defect engineering to promote catalytic activity.^185,209^ To exploit these advantages, Wang’s team synthesized sulfur- and carbon-*co*-doped FeNiCeP nanosphere arrays (SC-FeNiCeP/NF) via a two-step hydrothermal and chemical vapor deposition process.^183^ Initially, FeNiCe-TDC nanoflowers were grown on nickel foam using Fe, Ni, and Ce metal ions with 2,5-thiophene dicarboxylic acid (H_2_TDC) as the ligand. The nanoflowers were later phosphated by sodium hypophosphite (NaH_2_PO_2_) to get SC-FeNiCeP/NF (illustrated in the scheme in Figure 27A). This enhancement stems from two key factors: first, the Fe–Ni–Ce interface effect, which promotes electron coupling and redox-driven pseudocapacitance, and second, the sulfur-doped carbon framework makes metal phosphide sites more accessible and changes electronic properties. The different extent of sulfur doping changes the electron spin density within the carbon matrix, specifically when sulfur being larger in atomic size enters the carbon framework to help transfer charges and to diffuse ions, as illustrated in the schematic on the right-hand side of Figure 27A. The incorporation of cerium, with its d^0^ electron configuration, further fine-tunes electron distribution at active site interfaces, while the codoping strategy enhances carbon atom polarization, improving electron and ion diffusion. Bimetallic MOFs on NF have spherical (FeCe-TDC) and flower cluster (FeNi-TDC, NiCe-TDC) geometries, whereas monometallic MOFs have rectangular bulk (Fe-TDC), sheet (Ni-TDC), and grass ball (Ce-TDC) morphologies. Monometallic and bimetallic phosphides exhibit bulk and irregular nanoparticles, whereas the trimetallic FeNiCe-TDC forms flower clusters and its derived S, C codoped phosphide (SC-FeNiCeP/NF) manifests as nanospheres (Figure 27B). Among all monometallic and bimetallic electrocatalysts, only the trimetallic SC-FeNiCeP/NF electrocatalyst present low overpotential with 208 mV and 107 mV at 10 mA cm^–2^ in OER and HER, respectively (Figure 27C). Remarkably, at a current density of 10 mA cm^–2^, the OWS device assembled by SC-FeNiCeP/NF||SCFeNiCeP/NF needs an applied potential of 1.53 V. Adding high-valence Ce to Ni_2_P and Fe_2_P raises electron density at the Fermi level, improving electron transfer in SC-FeNiCeP/NF, according to DFT simulations using Vienna Ab initio Simulation Package (VASP) with Generalized Gradient Approximation (GGA) and Perdew–Burke–Ernzerhof (PBE). Higher state densities in Ni2P–Ce and Fe2P–Ce, including Fe2P@Ce, indicate that Ce doping enhances conductivity. The unstable surface of Fe_2_P causes structural alterations, proving that high-valence metal engineering is a successful tactic for multifunctional catalysts (Figure 27D).

**Figure 27:**
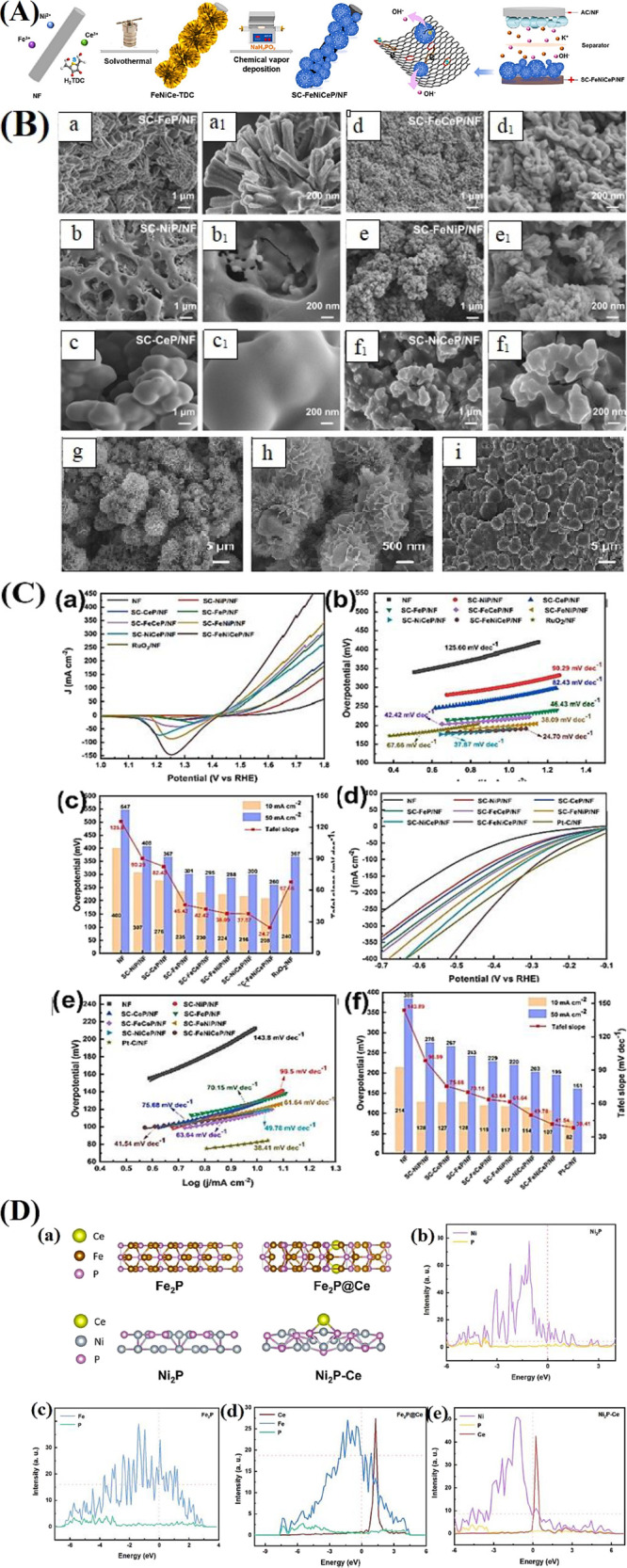
(A) Schematic illustration of SC-FeNiCeP/NF nanoarray synthesis and SC-FeNiCeP/NF||AC/NF properties. (B) SEM images of SC-FeP/NF (a, a_1_), SC-NiP/NF (b, b_1_), SC-CeP/NF (c, c_1_), SC-FeCeP/NF (d, d_1_), SC-FeNiP/NF (e, e_1_), and SC-NiCeP/NF (f, f_1_), FeNiCe-TDC (g)­(h), SC-FeNiCeP/NF (i). (C) Remarkable OER and HER performance of the as-obtained catalysts: (a) and (d) LSV curves; (b) and (e) Tafel plots; (c) and (f) data summary. (D) (a) Structure models for Fe_2_P, Fe_2_P@Ce, Ni_2_P, and Ni_2_P–Ce. Calculated partial density of states of Ni_2_P (b), Fe_2_P (c), Fe_2_P@Ce (d), and Ni_2_P–Ce (e).

Copyright 2024, Elsevier.^183^

The pore-space-partition (PSP) is an alternative mechanism available to stabilize MOFs that introduces partitioning agents for MOF spatial division. However, organic partitioning agents might adversely affect conductivity, thus limiting their application in electrocatalysis.^210^ To address this limitation, vanadium-based multimetallic MOFs offer a viable solution due to vanadium’s diverse oxidation states, high abundance, and exceptional electrochemical properties. Vanadium further enhances conductivity and drastically lowers overpotentials for rate-determining steps, allowing tuning of intermediate binding energies and promotion of metal center synergies.^211^ Using terephthalic acid (BDC) as the organic linker and 2,4,6-tri­(4-pyridyl)-1,3,5-triazine (TPT) as a pore-partitioning agent, a series of trimetallic vanadium-based MOFs, including Fe_2_V-MOF, Co_2_V-MOF, Mg_2_V-MOF, Ni_2_V-MOF, and Zn_2_V-MOF, were prepared in a solvothermal manner (Figure 28A).^184^ As shown in the SEM image in Figure 28B, the five M_2_V-MOFs show unique morphologies of spindle-like morphology for Fe_2_V-MOF, Co_2_V-MOF, and Ni2 V-MOF; and hexagonal, tower-like, and dendritic features for Mg_2_V-MOF and Zn_2_V-MOF. These materials exhibited exceptional electrocatalytic performance for both OER and HER, with Fe_2_V-MOF achieving ultralow overpotentials of 314 mV for OER and 198 mV for HER at 10 mA cm^–2^ (refer to Figure 28C and D). Furthermore, the Fe_2_V-MOF/NF||Fe_2_V-MOF/NF couple only needs 1.74 and 1.94 V to deliver current densities of 10 and 50 mA cm^–2^, respectively. But in addition to structural engineering, heteroatom doping and controlled pyrolysis yield defect-rich frameworks with unsaturated active sites that improve catalytic performance. For example, the nitrogen and carbon codoped MOF-derived TMPs exhibited excellent application in water electrolysis. Phosphorus and nitrogen species are electron-rich, and they adsorb protons to generate hydrogen, and metal cations adsorb hydroxyl ions to generate oxygen. Carbon doping inhibits agglomeration and improves charge transfer, catalytic stability, and conductivity. The pyrolysis of MOFs, in the synthesis of nanostructured materials, that retains their complicated structures brings about highly functional material. Among the earliest classes, metal–organic frameworks, synthesized via coordination chemistry, Ni–Fe–Mn-MOFs employ 2-aminoterephthalic acid as a coordinating ligand to attain very high porosity and specific surface area suitable for efficient mass transport. The closely matched atomic radii of Ni, Fe, and Mn further contribute to the formation of robust interconnected MOFs in conjunction with 2-aminoterephthalic acid as the coordinating agent. The pyrolysis of these MOFs produced nitrogen and carbon codoped Ni–Fe–Mn–P/NC composites whose carbon matrix acts to prevent aggregation and maintain active sites. Ni^2+^, Fe^3+^, and Mn^2+^ are present with the nitrogen in addition to phosphorus of the MOF-derived phosphide Ni–Fe–Mn–P/NC@NF to affect control over the threshold for hydrogen and oxygen gases to evolve. The resultant Ni–Fe–Mn–P/NC@NF architecture, comprising mesoporous nanosheets, significantly enhances electrode–electrolyte interactions, establishing it as a high-performance electrocatalyst for HER and OER in energy conversion applications.^185^

**Figure 28:**
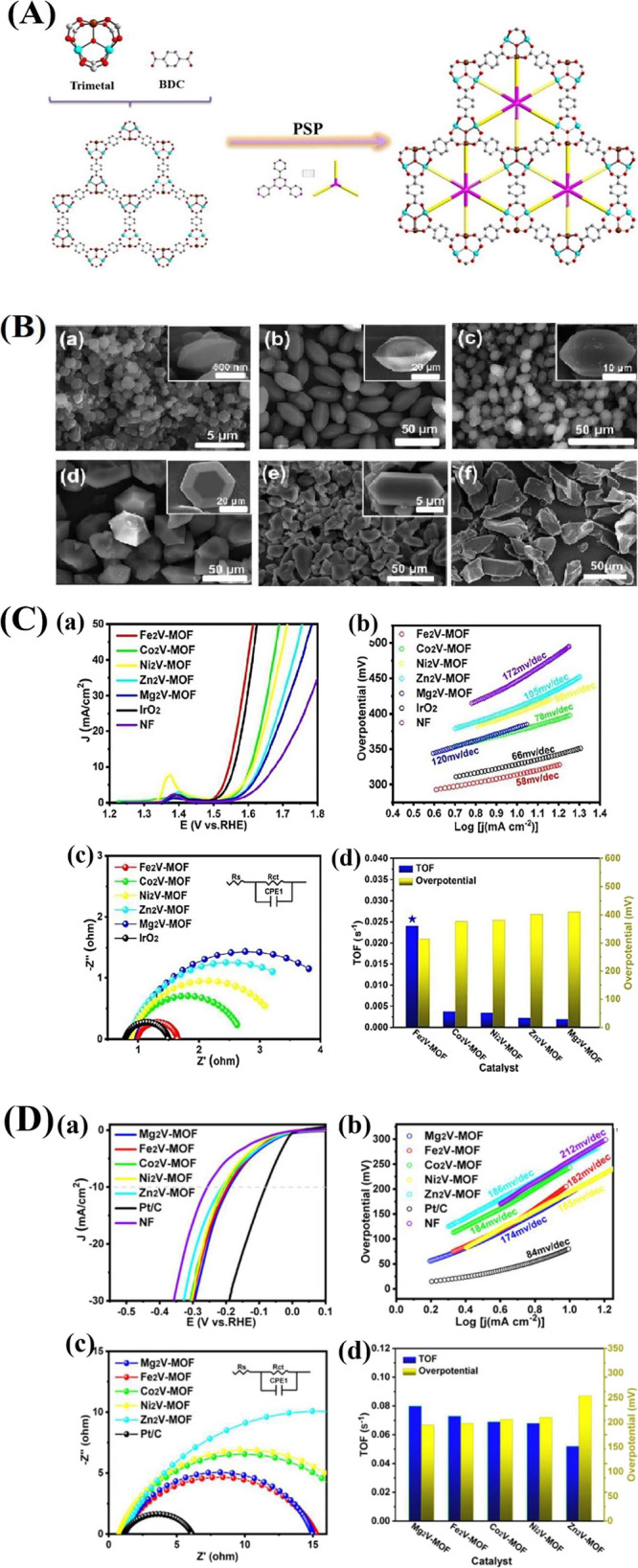
(A) Synthesis process of M_2_V-MOFs based on the PSP strategy. (B) The SEM images of (a) Fe_2_V-MOF, (b) Co_2_V-MOF, (c) Ni_2_V-MOF, (d) Zn_2_V-MOF, (e) Mg_2_V-MOF, and (f) Mg_2_V-MIL-88. (C) OER results and (D) HER of M_2_V MOFs and NF: (a) polarization curves, (b) Tafel plots, (c) EIS spectra, (d) TOF, and overpotentials.

Copyright 2022, ACS.^184^

Metal–organic gels (MOGs) are a special class of materials combining properties of MOFs and coordination polymers, while they are also different from supramolecular gels that arise from noncovalent interactions.^212^ They are formed by coordinating metal ions together with some organic ligands to yield fibers, layered, or granular structures.^213^ Weak intermolecular forces hold these assemblies together, forming a 3D mesh with a porous framework, multifunctional ligands, and active metal sites.^214,215^ MOGs also show good electrical conductivity and can be synthesized simply in bulk and used as catalytic centers in electrochemical applications.^216^ For enhanced catalytic performance, a trimetallic organic gel, CoFeNi-MOG, is introduced as an optimized pre-electrocatalyst.^134^

### 5.3 Engineering Trimetallic LDH for Electrocatalytic Performance

LDHs that use transition metals are becoming promising electrocatalysts for splitting water in alkaline environments.^217^ But restrictions such as uncontrollable stacking 2D nanosheets and poor conductivity lower their electrocatalytic activity, decreasing active surface areas and blocking electron transfer. To counter these, researchers have attempted to enhance LDHs by doping with transition metals or heteroatoms, which increases electronic structure, avoids local dissolution, and enables electrolyte permeation.^218,219^ Recent investigations have demonstrated that cobalt-based LDHs exhibit enhanced mass transfer for OER due to synergistic interactions between Co and Fe species, nanoscale ion transport, and expanded interlayer spacing. Despite these advantages, their conductivity limitations and strong hydrogen adsorption energy restrict their overall efficacy.^217^ By adjusting the 3d energy levels of LDHs, cationic regulation with high-valence dopants such as Mo^6+^, V^5+^, Cr^3+/6+^, and Zr^4+^ improves electron interactions and optimizes intermediate adsorption energies. Because Zr^4^^+^ doping enhances activity in OER, the CoFeZr/NF electrocatalyst outperforms CoFe/NF and RuO_2_ with an overpotential of 233 mV at 10 mA cm^–2^ and a Tafel slope of 40.9 mV dec^–1^. Regarding HER, with a lower Tafel slope of 132.7 mV dec^–1^ in relation to NF (140.7 mV dec^–1^) and CoFe/NF (139.4 mV dec^–1^), CoFeZr/NF also exhibits a lower overpotential of 159 mV at 10 mA cm^–2^ than NF (245 mV) and CoFe/NF (193 mV). This suggests faster HER kinetics with Zr doping. CoFeZr/NF maintains high activity and stability for more than 20 h in a seawater-splitting device working as both anode and cathode, and it displays strong bifunctional activity with little decay in alkaline simulated seawater.^220^ To further improve the water splitting efficiency, the incorporation of LDHs with metal sulfides or selenides has been proposed to enhance conductivity and stability.

Although CoFe LDHs as standalone water splitting catalysts have been proven ineffective, interface-engineered hybrid nanomaterials based on synergistic effects from the integration of LDHs with other nanocomponents have been created by researchers. For example, Zhang et al. fabricated a CoFe-LDH-CuCo_2_S_4_ electrocatalyst on nickel foam (NF) by hydrothermal and electrodeposition methods.^186^ Figure 29A shows the schematic sketch of synthetic steps where the hierarchical CoCu-LDH nanowires were directly grown on the NF substrate and interwove each other to generate a 3D interconnected architecture. The porosity of the nanowires provides rich active sites and facilitates fast electrolyte and reactant diffusion and thus increases mass transfer. Immersion of the CoCu-LDH/NF precursor in Na_2_S solution results in ion exchange, producing CuCo_2_S_4_/NF. Following vulcanization, the nanowire structure remains intact, with an increase in surface roughness. Layers of CoFe-LDH were successfully synthesized and coated onto CuCo_2_S_4_ nanowires to assemble into a flower-like microspheric 3D hierarchical architecture of highly ordered nanosheets. At an overpotential of 63 mV, CuCo_2_S_4_@CoFe-LDH/NF attains a current density of 10 mA cm^–2^ for HER, which is lower than those of CoFe-LDH/NF (198 mV), CuCo_2_S_4_/NF (107 mV), CoCu-LDH/NF (77 mV), and NF (260 mV). Its Tafel slope of 59 mV dec^–1^ suggests faster electron transfer and reaction kinetics than those of 138 mV dec^–1^ (CoFe-LDH/NF), 135 mV dec^–1^ (CuCo_2_S_4_/NF), 96 mV dec^–1^ (CoCu-LDH/NF), and 167 mV dec^–1^ (NF). The HER may adhere to the Volmer–Heyrovsky or Volmer–Tafel mechanism, according to the Tafel slope (Figure 29B). With a clear Co^2+^ to Co^3+^ oxidation peak at 1.42 V, indicating substantial Co site activity, the LSV curves for OER (Figure 29C) shows that CuCo_2_S_4_@CoFe-LDH/NF reached an overpotential of 293 mV at 50 mA cm^–2^, lower than CoFe-LDH/NF (313 mV), CuCo_2_S_4_/NF (363 mV), CoCu-LDH/NF (385 mV), and NF (563 mV). Compared to CoFe-LDH/NF (103 mV dec^–1^), CuCo_2_S_4_/NF (123 mV dec^–1^), CoCu-LDH/NF (153 mV dec^–1^), and NF (230 mV dec^–1^), its Tafel slope of 73 mV dec^–1^ is lower, indicating improved kinetics. Compared to the CoFe-LDH/NF || CoFe-LDH/NF (1.76 V), CuCo_2_S_4_/NF || CuCo_2_S_4_/NF (1.68 V), CoCu-LDH/NF || CoCu-LDH/NF (1.63 V), and NF || NF (1.89 V) electrolyzers, the CuCo_2_S_4_@CoFe-LDH/NF || CuCo_2_S_4_@CoFe-LDH/NF water electrolyzer needs just 1.54 V at 10 mA cm^–2^.

**Figure 29:**
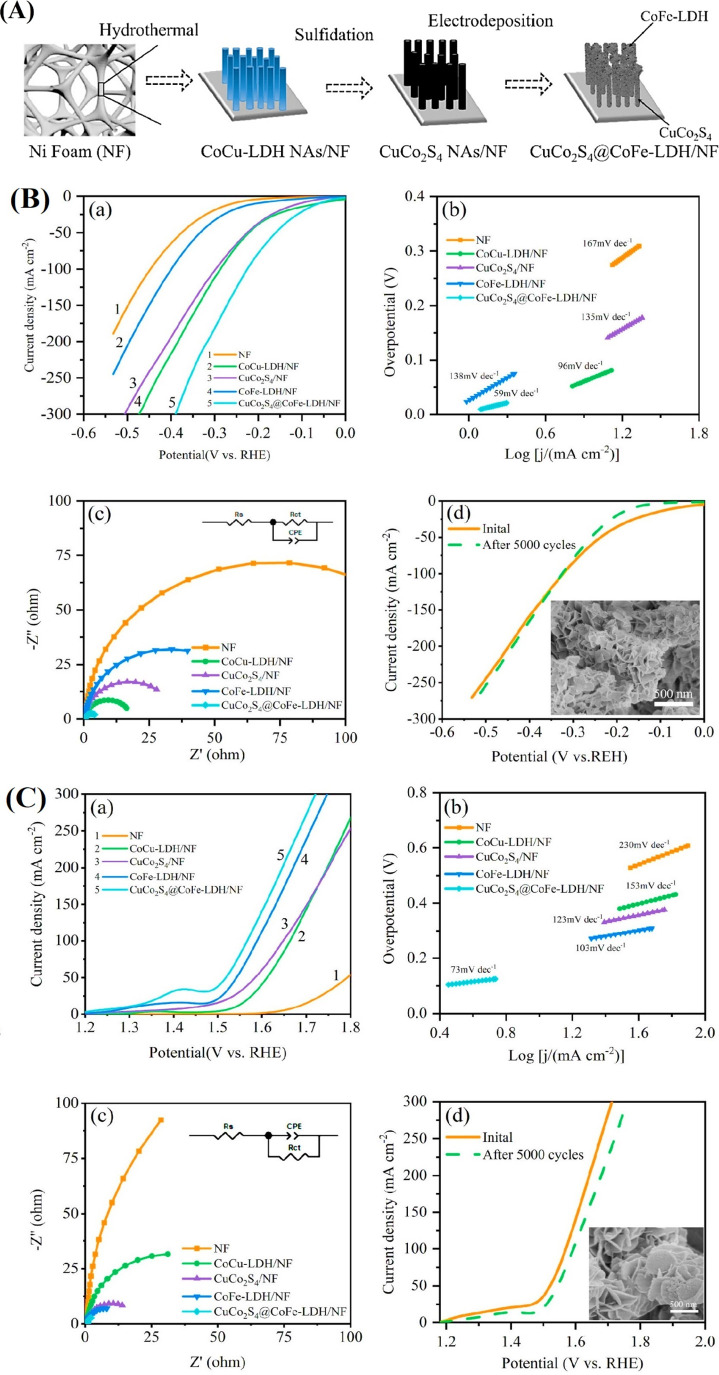
(A) Synthetic schematic of CuCo_2_S_4_@CoFe-LDH/NF. (B) SEM images of NF (a), CoCu-LDH/NF (b–c), and CuCo_2_S_4_/NF (d). SEM images of the CuCo_2_S_4_@CoFe-LDH/NF (e–h). (C) HER and (D) OER results: (a) LSV curves, (b) Tafel slope plots, (c) EIS, and (d) LSV curves for CuCo_2_S_4_@CoFe-LDH/NF before and after 5000 CV cycles and SEM image after 5000 CV cycles.

Copyright 2024, Elsevier.^186^

Materials of the spinel types with certain chemical compositions (i.e., AB_2_O_4_ or AB_2_S_4_) show great promise for many electrochemical applications. For instance, CuCo_2_S_4_, NiCo_2_S_4_, MnCo_2_S_4_, and FeCo_2_S_4_ are promising for electrochemical applications due to their superior electronic conductivity, redox properties, abundant availability, cost-effectiveness, and nontoxic properties. In CuCo_2_S_4_, Cu^2+^ and Co^3+^ ions occupy tetrahedral and octahedral sites, respectively, enhancing catalytic activity. The synergy between CoFe-LDHs and CuCo_2_S_4_ further improves water-splitting performance. Transition metal doped trimetallic sulfides possess enhanced conductivity and stability that increrase structural resilience and performance of electrocatalysts due to nickel and cobalt sharing similar atomic radii. Molybdenum disulfide (MoS_2_), especially in the 1T phase, is a natural layered material with high conductivity and plenty of active sites for HER, which has a great potential in energy storage and conversion.^221,222^ A bifunctional heterostructure electrocatalyst synthesized in situ, consisting of transition metal-doped MNiCoS_4_ (M = Cu, Fe, Zn, Mn) and 1T-MoS_2_, was developed on a nickel foam (NF) substrate, which delivered an increased density of active sites and compared to distinctive advantages of current materials (Figure 30A).^187^ The incorporation of a third metal, coupled with the spatial confinement of intercalated Mo_7_O_24_^6–^ within trimetallic LDH layers and the structural constraints of nickel foam, induces lattice distortions and vacancies in the spinel sulfides and MoS_2_. These adjustments improve intermediate adsorption energies and thus catalytic activity.^223^ At an overpotential of 163 mV, CuNiCoS_4_/1T-MoS_2_ attains a current density of 50 mA cm^–2^ for OER, which is lower than those of RuO_2_ (299 mV), NiCo_2_S_4_ (359 mV), CuNiCoS_4_ (303 mV), and NiCo_2_S_4_/1T-MoS_2_ (196 mV). When compared to NiCo_2_S_4_ (126.4 mV dec^–1^), CuNiCoS_4_ (101.1 mV dec^–1^), and NiCo_2_S_4_1T-MoS_2_ (84.8 mV dec^–1^), its Tafel slope of 53.5 mV dec^–1^ (Figure 4b) is the smallest, indicating the fastest reaction kinetics. With an HER overpotential of 160 mV at 10 mA cm^–2^, CuNiCoS_4_/1T-MoS_2_ outperforms NiCo_2_S_4_ (228 mV), CuNiCoS_4_ (230 mV), and NiCo_2_S_4_/1T-MoS_2_ (172 mV). In addition, in a two-electrode configuration, this catalyst achieves a current density of 10 mA cm^–2^ at a remarkably low voltage of 1.52 V, underscoring its favorable HER activity (Figure 30B).^224^

**Figure 30:**
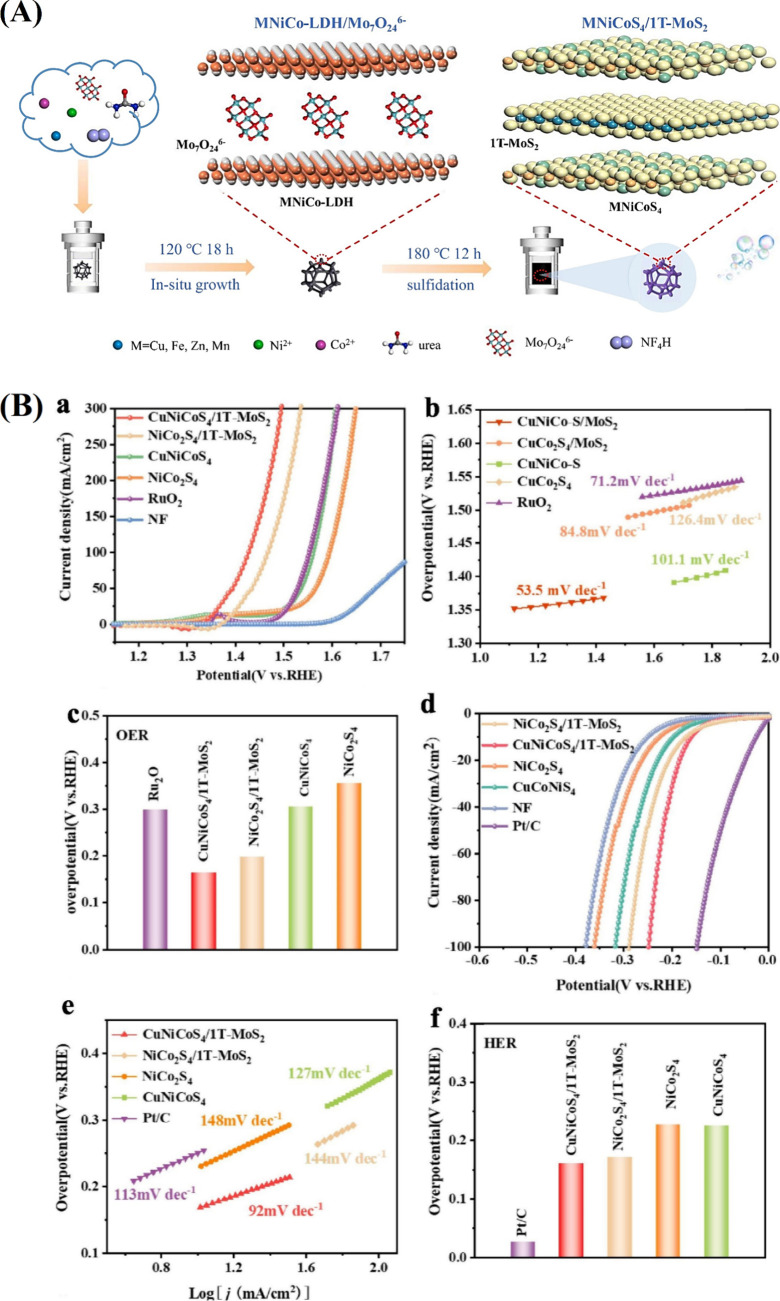
(A) Schematic illustration of the synthetic strategy for MNiCoS_4_ (M = Cu, Fe, Zn, Mn)/1T-MoS_2_. (B) (a) OER LSV curves and (b) corresponding Tafel slopes in 1 mol L^–1^ KOH solution. (c) Overpotential at 50 mA cm^–2^. (d) HER LSV curves and (e) corresponding Tafel slopes in 1 mol L^–1^ KOH solution. (f) Overpotential at 10 mA cm^–2^.

Copyright 2024, Elsevier.^187^

Combining materials that conduct electricity well with LDHs helps electrons to move more efficiently at the interface of the materials. Transition metal selenides, exhibiting superior conductivity due to strong M-Se bonds and vacant 3d orbitals, improve charge transport in water splitting. The Ni–Co–Fe-Se@NiCo-LDH heterostructure, fabricated on nickel foam, demonstrates increased active sites and conductivity compared to single-metal selenides. During the hydrothermal reaction, urea gradually releases OH^–^ and CO_3_^2–^ ions, which combine with metal ions to precipitate. The variation in precipitator and ammonium fluoride may be the reason for the morphological variance (Figure 31A).^188^ Prior to the addition of a third metal, the surface morphology of CoFe-LDH@NiCo-LDH/NF and NiCo-LDH/NF is composed of laminated nanosheets. Following selenization, NiCo-LDH/NF changes into a structure resembling a nanopore with surface particles, generating nanospheres on the nanosheets. These nanospheres enhance specific surface area and electrolyte contact in Ni–Co–Se@NiCo-LDH/NF, forming mesoporous channels for the release of hydrogen and oxygen bubbles. As illustrated in Figure 31B for OER electrocatalysis, in comparison to Ni–Co–Se@NiCo-LDH/NF (222 mV), CoFe-LDH@NiCo-LDH/NF (246 mV), and NiCo-LDH/NF (357 mV), the Ni–Co–Fe-Se@NiCo-LDH/NF exhibits overpotentials of 178 mV at a current density of 10 mA cm^–2^. The following four steps comprise the OER process: MO + OH^–^ → MOOH + e^–^; MOOH + OH^–^ → M + O_2_ + H_2_O + e^–^; M + OH^–^ → MOH + e^–^, and MOH^+^ + OH^–^ → MO + H_2_O + e^–^. Furthermore, the bare NF is 150.5 mV dec^–1^, the Tafel slope of Ni–Co–Fe-Se@NiCo-LDH/NF is 78.9 mV dec^–1^, that of CoFe-LDH@NiCo-LDH/NF is 107.42 mV dec^–1^, that of Ni–Co–Se@NiCo-LDH/NF is 94.6 mV dec^–1^, and that of NiCo-LDH/NF is 123.3 mV dec^–1^. It is evident that Ni–Co–Fe-Se@NiCo-LDH/NF has the best reaction kinetics because it has the lowest Tafel slope. Regarding HER, Figure 31D demonstrates that only overpotentials of 113 mV are needed for Ni–Co–Fe-Se@ NiCo-LDH/NF, 189 mV for Ni–Co–Se@NiCo-LDH/NF, 212 mV for CoFe-LDH@NiCo-LDH/NF, and 246 mV for NiCo-LDH/NF, when a current density of 10 mA cm^–2^ needs to be driven. Ni–Co–Fe-Se@NiCo-LDH/NF clearly exhibited the highest HER activity. Compared to Ni–Co–Se@NiCo-LDH/NF (124.09 mV dec^–1^), CoFe-LDH@NiCo-LDH/NF (119.11 mV dec^–1^), NiCo-LDH/NF (152.02 mV dec^–1^), and bare NF (246.6 mV dec^–1^), Ni–Co–Fe-Se@NiCo-LDH/NF has a lower Tafel slope of 44.87 mV dec^–1^ (Figure 31 C). A current density of 10 mA cm^–2^ could be reached with a cell voltage of just 1.55 V when the material was employed as a bifunctional catalyst in a dual motor system. According to the DFT calculation, the presence of Co-NiSe_2_ material speeds up the kinetics of hydrogen production, whereas Fe_7_Se_8_ material improves the material’s conductivity.

**Figure 31:**
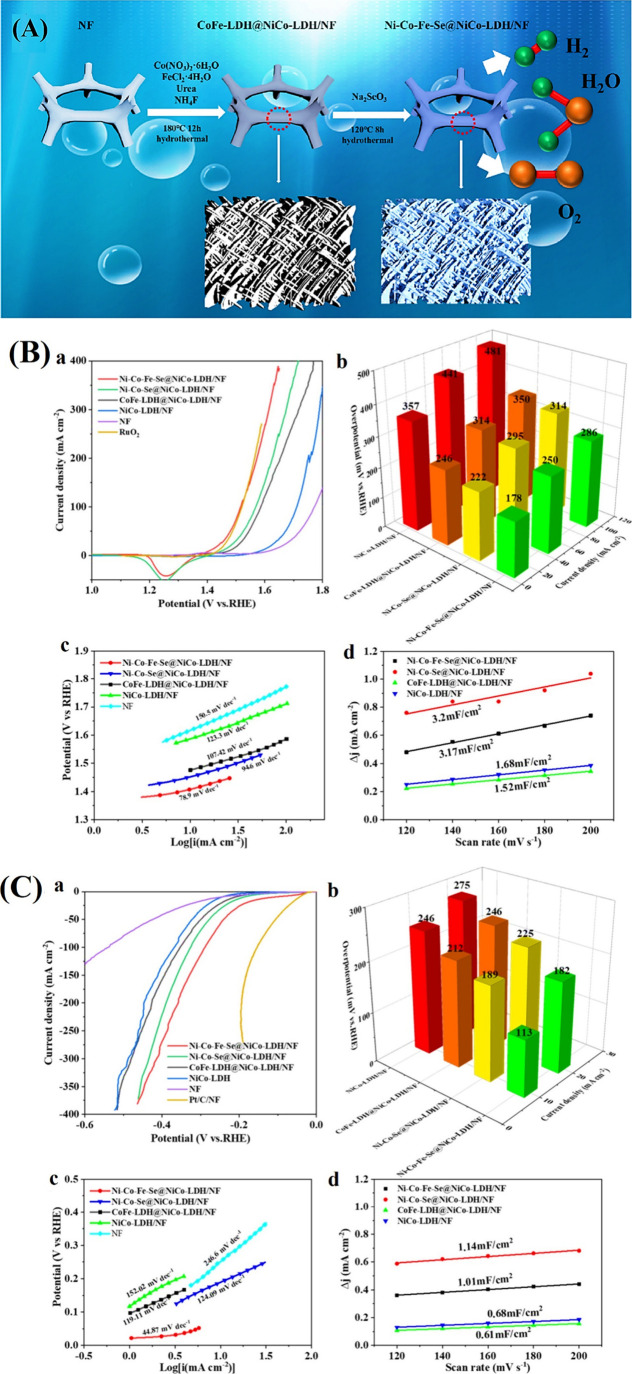
(A) Scheme of the design process of Ni–Co–Fe-Se@NiCo-LDH/NF. (B) OER and (C) HER performance tests of Ni–Co–Fe-Se@NiCo-LDH/NF, CoFe-LDH@NiCo-LDH/NF, Ni–Co–Se@NiCo-LDH/NF, NiCo-LDH/NF, bare NF, RuO_2_, and Pt/C/NF: (a) LSV curves (*iR* compensation of 70%), (b) corresponding overpotentials, (c) Tafel slopes, and (d) *C*_dl_.

Copyright 2023, Elsevier.^188^

Synergistic interaction of Co^2^^+^, Ni^2^^+^, and Fe^3^^+^ ions in the catalytic structure increases activity and stability even more. Furthermore, using high-conductivity nickel foam enhances conductivity and electrochemically active surface area, especially with 2D hydroxide nanosheets integrated into a 3D architecture.

The CF-anchored 3D CoNiCu-LDH microflowers demonstrated excellent OER and HER activity in 1 mol L^–1^ KOH, which results from the strong interaction between the LDH and substrate, intrinsic porosity, and improved conductivity.^189^ Integrating heterostructures in composite catalysts optimizes surface properties, increases active sites, and improves adsorption energy, accelerating electrocatalytic dynamics.^219^ To improve their performance and widen their implementation as specific OER catalysts, it is very significant to tackle the drawback of LDHs, especially with regard to the shortage of HER-active sites. In overcoming the disadvantage of LDHs, particularly the restricted HER-active sites, incorporating a tertiary TM into binary TM-LDHs has been tested and brought about optimized morphology and electronic structure. TM sulfides, owing to their unique d-electron configurations and superior conductivity, have emerged as promising catalytic enhancers.^219^ A ternary NiCoFe-LDH/NiCoFeS composite catalyst was built upon Ni foam through a hydrothermal and sulfide process, assuring morphology retention and improved interfacial properties.^143^ A following secondary hydrothermal treatment in an Fe^3^^+^ environment facilitated in situ ternary NiCoFe-LDH and NiCoFe sulfide growth to enhance electronic structure and interfacial interaction (Figure 32A). In this composite, Fe^3+^ ions actively participate in the dissolution-recrystallization process, incorporating into the lattice and modifying the electronic structure to enhance endogenous activity. The hydrothermal treatment optimizes Fe integration, enhancing interfacial regions, electronic correlations, and adsorption energies. The sulfide increases the conductivity and catalytic activity, while the nanofloral appearance increases the surface area, benefiting the activity. The heterostructure interface enhances charge transfer and optimizes electronic properties, significantly improving the overall electrocatalytic efficiency of the system. The clustered nanoflower-like structures of the NiCoFeLDH/NiCoFeS–NF catalyst improve performance by facilitating the production and distribution of bubbles, increasing the electrolyte-catalyst contact area, and boosting mass transport for increased catalytic efficiency. With sulfide incorporation improving OER performance, the NiCoFeLDH/NiCoFeS–NF catalyst needs only 241 mV to reach 100 mA cm^–2^, which is less than S–NiCo­(OH)_2_–NF (300 mV), NiCo­(OH)_2_–NF (360 mV), and unsulfurized NiCoFeLDH-NF (270 mV). Fe^3^^+^ addition during subsequent hydrothermal treatment enhances active sites and permits synergistic electron and charge transfer in the NiCoFeS–NF sample, which achieves a 260 mV overpotential. The NiCoFeLDH/NiCoFeS–NF improved kinetics are indicated by its lower Tafel slope of 31.51 mV dec^–1^ compared to NiCo­(OH)_2_–NF (91.45 mV dec^–1^), S–NiCo­(OH)_2_–NF (88.90 mV dec^–1^), NiCoFeLDH-NF (45.22 mV dec^–1^), and NiCoFeS–NF (39.39 mV dec^–1^) (Figure 32 B). The catalyst outperformed NiCo­(OH)_2_–NF (231 mV), NiCoFeS–NF (207 mV), and NiCoFeLDH-NF (348 mV) with an overpotential of 191 mV at 100 mA cm^–2^ for HER. It also matched S–NiCo­(OH)_2_–NF, demonstrating the advantage of sulfidation. The high electronegativity of S sites, which facilitate proton adsorption, and S doping, which modifies the electronic structure and coordination of neighboring metals, are responsible for the increased activity. With a Tafel slope of 90.54 mV dec^–1^, it exhibits better kinetics than NiCo­(OH)_2_–NF (113.28 mV dec^–1^), S–NiCo­(OH)_2_–NF (107.14 mV dec^–1^), and FeCoNiS–NF (109.19 mV dec^–1^) (Figure 32C). In thorough water splitting testing, the NiCoFeLDH/NiCoFeS-NF catalyst can produce a current density of 50 mA cm^–2^ with just a 1.63 V battery voltage.

**Figure 32:**
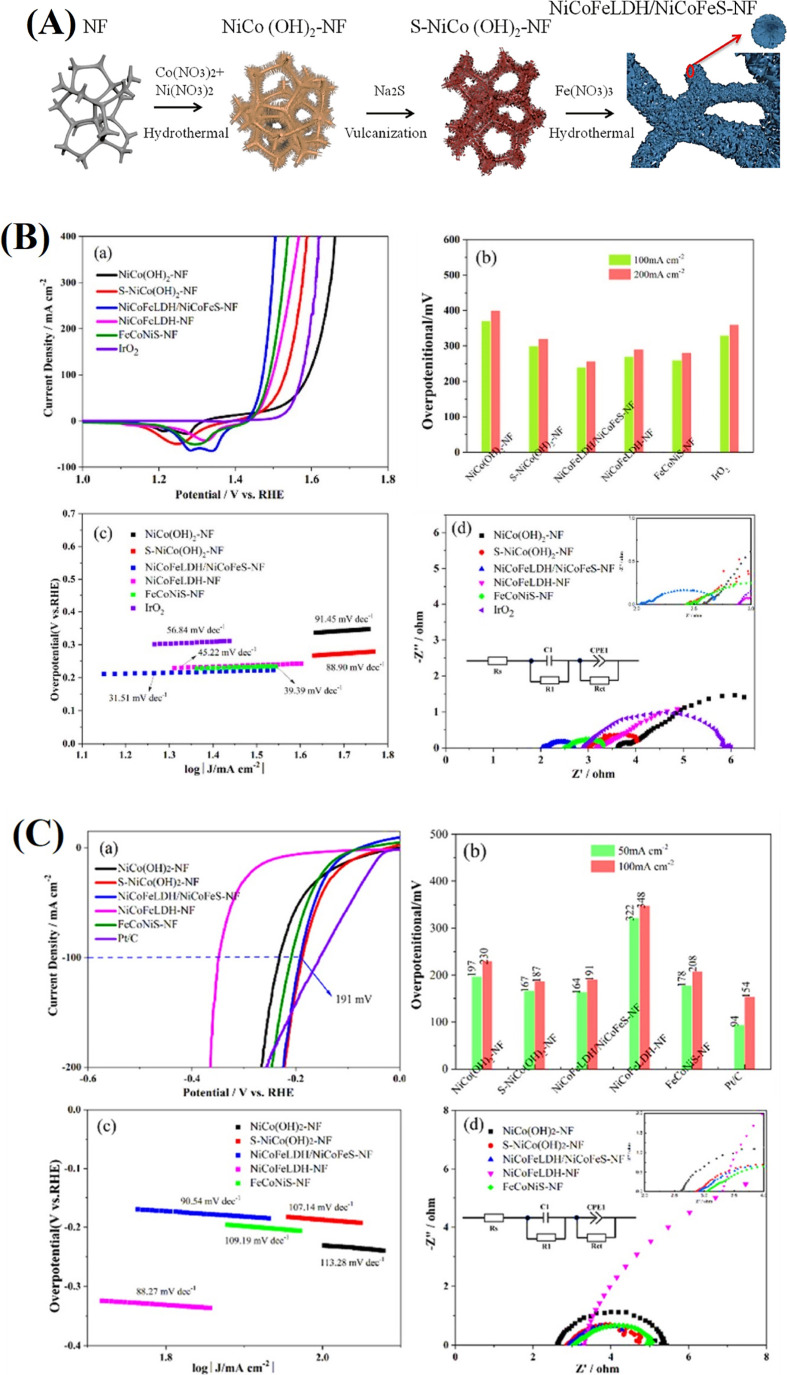
(A) Synthesis of the NiCoFeLDH/NiCoFeS–NF. (B) LSV polarization curves for OER after *iR*-corrected (a), overpotentials at 100 mA cm^–2^ and 200 mA cm^–2^ (b), Tafel slopes (c), EIS spectra tested at 1.57 V vs RHE (d) of the NiCo­(OH)_2_–NF, S–NiCo­(OH)_2_–NF, NiCoFeLDH/NiCoFeS–NF, NiCoFeLDH-NF, FeCoNiS–NF, and IrO_2_ in 1 mol L^–1^ KOH solution. (C) LSV polarization curves for HER after *iR*-corrected (a), overpotentials at 50 mA cm^–2^ and 100 mA cm^–2^ (b), Tafel slope (c), EIS spectrum tested at −0.27 V vs RHE (d) for NiCo­(OH)_2_–NF, S–NiCo­(OH)_2_–NF, NiCoFeLDH/NiCoFeS–NF, NiCoFeLDH-NF, FeCoNiS–NF, and Pt/C in 1 mol L^–1^ KOH solution.

Copyright 2024, Elsevier.^143^

Vanadium doping enhances LDH conductivity, optimizes electron transfer, modifies the electronic structure, increases active sites, and improves hydrogen and oxygen evolution. Its interlayer space also aids charge carrier stabilization. NiFe-LDH catalysts excel in alkaline conditions due to Ni–Fe synergy. Vanadium doping mitigates lattice imperfections and spatial constraints in Fe- and Ni-doped catalysts, enhancing their catalytic performance. It was demonstrated that high temperature sulfidation techniques increase the specific surface area, stabilize the catalyst, and enhance its electrocatalytic efficiency. Thus, the design of trimetallic composites would serve as a novel strategy in the development of high-performance water-splitting electrocatalysts.

He et al. has developed a new NiFeVSx@NF catalyst in which some vanadium and some sulfide ions have been combined with NiFe-LDH to form a ternary NiFeVSx compound that has the structure of a sea-urchin. For this corrugated landscape, B dust–dust sulfur (BS_4_) rods were synthesized in 2 phases directly on a NiFe-LDH nanoplate array, by combinational hydrothermal treatment followed by the second high-temperature sulfidation. This design improves the catalyst surface area and charge transfer rates, by reducing electrode-bubble contact, increasing bubble release and ion transport. Desirable lattice faults to the catalyst also come due to the structure of LDH that support stability for the catalyst (Figure 33A). Following the initial reaction step, NiFe-LDH@NF generates a disordered, compact nanosheet array. The second step produces NiFeV-LTH@NF by growing rod-like V hydroxide in situ on NiFe-LDH. With denser, longer V-based rods firmly bonded to nanosheets, NiFeVS_*x*_@NF maintains its structure after vulcanization, creating a sturdy, sea urchin-like three-dimensional nanostructure that increases stability and catalytic surface area. In comparison to NiFe-LDH@NF (256 mV) and NiFeV-LTH@NF (209 mV) (Figure 33B), NiFeVS_*x*_@NF needs an overpotential of 127 mV to reach 10 mA cm^–2^ for HER. This is because the three metal sulfides work in concert to improve hydrogen intermediate adsorption/desorption and hydrogen production. As demonstrated by the Tafel slope of NiFeVS_*x*_@NF (121 mV dec^–1^), which is lower than those of NiFe LDH@NF (236 mV dec^–1^) and NiFeV LTH@NF (147 mV dec^–1^), vulcanization optimizes catalytic properties. This suggests improved electron exchange because of the lone pair electrons of Fe^2+^ and rapid HER kinetics through the Volmer–Heyrovsky mechanism.^190^ With respect to OER, because of changes in ion interactions and electron transport, the overpotentials for NiFe-LDH@NF, NiFeV-LTH@NF, and NiFeVS_*x*_@NF for OER to reach 10 mA cm^–2^ are 281, 269 mV, and 259 mV, respectively. NiFeVS_*x*_@NF exhibits the lowest Tafels slopes, which are 141, 97 mV dec^–1^, and 34 mV dec^–1^, respectively (Figure 33C). This is explained by trivalent vanadium doping, which increases the oxidation capacity and electron transfer, increasing OER catalytic activity and turnover frequency. Moreover, a current density of 10 mA cm^–2^ may be attained with just 1.6 V when NiFeVS_*x*_@NF is employed as a bifunctional electrode system for total water splitting.

**Figure 33:**
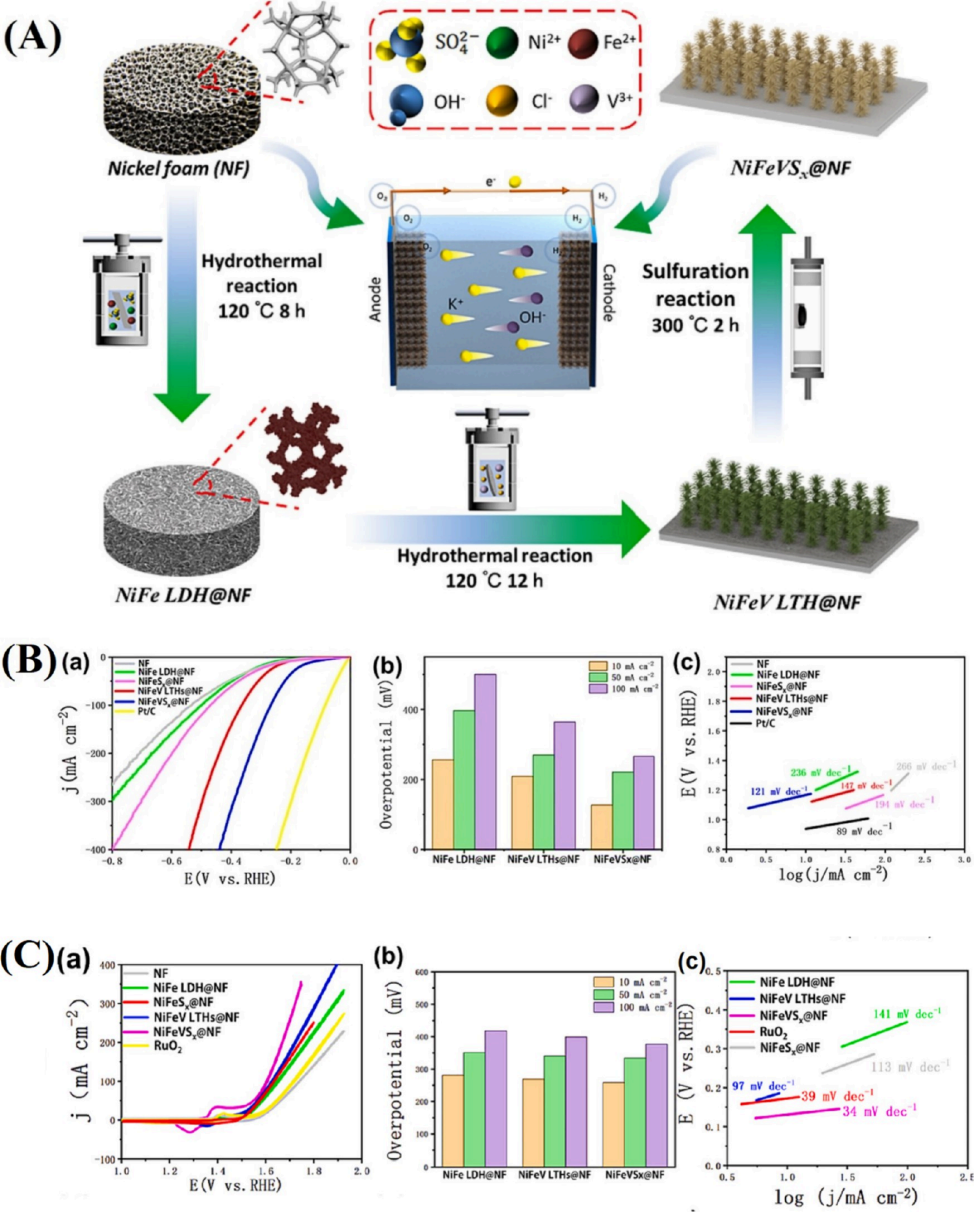
(A) Schematic illustration of the stepwise synthesis of NiFe LDH@NF, NiFeV LTH@NF, and NiFeVSx@NF for highly efficient total water splitting. (B) HER and (C) OER performance in a solution containing 1 mol L^–1^ KOH. (a) LSV curves, (b) the overpotentials, and (c) the corresponding Tafel plots of NiFe LDH@NF, NiFeV LTH@NF, and NiFeVSx@NF.

Copyright 2023, Elsevier.^190^

Improving electrocatalysts is a 2-fold process: enhancing intrinsic activity and active site density. Hybrid catalysts blending noble and non-noble metals take advantage of complementary properties and have been shown to be effective when doping and using varied metallic compositions. The most promising options for both OER and HER are earth-abundant transition-metal alloys, particularly binary Ni and Co oxides/hydroxides. A tertiary metal is added to ternary alloys, which greatly improve the process of water splitting by reducing overpotentials. Even exceeding noble metals including Pt in the HER thanks to good d-band characteristics that boost hydrogen adsorption, molybdenum-based catalysts are attracting interest for their strength and energy efficiency.^225^ Combining Mo-based alloys with metals including iron, cobalt, or nickel, raises active sites and boosts electrical conductivity. More Mo in Ni–Mo powders lowers hydrogen adsorption energy on Mo sites or produces oxygen vacancies, thereby improving HER activity. Little quantities of Fe might improve Ni-based OER catalysts.^226^ Alkaline media result in restructuring of molybdates to further boost catalysis.^227^ The surface of metal alloys undergoes reconstruction, formation of metal oxyhydroxides, selective dissolution, and morphological and structural defects under oxidative conditions. Thus, these alloys act as the precursors for in situ metal oxyhydroxide formation in the improvement of oxidative reactions. Creative catalytic methods, such as ternary alloys, are the most recent approaches that provide appropriate precatalysts with an anode reaction that is more efficient than precedent examples and that can have enhanced OER and HER activities. From the named structures, the Ni–Co hybrids are favorites for their abundance, cost-smartness, and high power capacity. Mo doping helps to achieve very high conductivity, low charge transfer resistance, and increased water dissociation sites that are plentiful with H* intermediates. The TMoO_4_ composition with more active sites of such as T = Ni or Co responds to water dissociation. High-valence Mo^6+^ incorporation is the reason for enhancing the electronic structure, catalytic activity, and availability of protons in alkaline solution.^228^ Due to similar hydrogen adsorption energy of Ni, Co, and Mo, together with the increased activity of binary metal oxide, these are able to reach the same effect as the Pt, and thence, these provide a similar activity in the oxygen reduction reaction.

## Conclusions and Future Perspectives

In order to produce hydrogen via HER and OER, electrochemical water splitting is a complicated process with slow kinetics and significant overpotentials that reduce efficiency. Despite being efficient electrocatalysts, noble metals such as Pt, Pd, IrO_2_, and RuO_2_ have slow kinetics and expensive costs, which restrict their scalability. Potential exists in earth-abundant materials; however, the four-electron OER process and HER are still difficult. For effective, economical hydrogen generation, it is essential to develop highly active, stable electrocatalysts and comprehend reaction mechanisms and structure–activity relationships.

Emerging as a rapidly evolving category of advanced materials, trimetallic nanostructures, especially those integrating first-row transition metals, hold significant appeal owing to their inherent structural adaptability, relative ease of molecular-level functionalization, adjustable porosity, and abundant active sites, and these materials offer remarkable potential for diverse applications in electrocatalysis, energy storage, and conversion. These multimetallic nanostructures exhibit superior performance, selectivity, stability, and recyclability compared to their mono- and bimetallic counterparts. Nevertheless, challenges persist in the widespread adoption of trimetallic materials for energy conversion applications. The utilization of trimetallic materials hinges upon intricate considerations of their structure, morphology, metal composition, and supporting materials, all of which necessitate meticulous control during the synthesis. Comprehensive studies are needed to elucidate their catalytic activity mechanisms, with particular emphasis on defect engineering, ligand effects, electronic properties, strain effects, interfacial phenomena, and surface area modifications. Optimizing surface and interfacial structures is paramount, and the identification of active sites in trimetallic systems, particularly under alkaline conditions, continues to be contentious, necessitating advanced characterization techniques and computational modeling to elucidate reaction mechanisms. Despite the promising performance and stability of trimetallic catalysts, particularly in anodic electrocatalytic processes, the scalability of these catalysts for industrial applications requires further exploration, as problems with long-term stability, economical synthesis, and compatibility with actual electrolytes (e.g., seawater) still exist.

1. Insufficient active sites: Bulk materials such as LDH and MOF are not optimal for water splitting reactions. Converting bulk materials into nanostructured forms can augment the number of electroactive sites, thereby providing more binding sites for electrolytes and enhancing water splitting activity.
2. Poor conductivity: Enhancing the conductivity of trimetallic-based materials can be achieved by binding or decorating them onto conductive supports such as carbon-based substrates, Ni foam, and Cu foam.
3. Lack of ion transport: Further improvement in the catalytic activity of transition metal-based oxides is necessary. Introducing ligands containing heteroatoms like N, P, and S can enhance the water splitting activity by fostering a synergistic effect among multiple metal ions. Ligands with multiple chelation sites can complex multiple metal ions and facilitate the formation of large cross-linked networks. Moreover, electron-rich species like phosphorus and nitrogen exhibit proton-capturing capabilities, resulting in the generation of hydrogen molecules. Conversely, positively charged metal cations effectively interact with hydroxyl ions and leverage the negative sites of phosphorus and nitrogen, thereby aiding in the release of oxygen molecules. This cooperative interplay among metal ions can be augmented through unique structural attributes, thereby promoting electron transfer during water splitting
4. Synthesis challenges: The fabrication of certain catalysts, such as chalcogenides, phosphides, and nitrides, often necessitates harsh reaction conditions, including high temperatures and pressures, resulting in the emission of hazardous gases. These conditions pose challenges for large-scale commercial production due to their unfavorable environmental impact and operational complexities.

In this comprehensive review, first, we have provided insights into the fundamental aspects of water-splitting reactions, elucidating the mechanisms underlying both half-cell reactions. Second, we have examined the latest developments in OER, HER and bifunctional electrocatalysts involving trimetallic materials based on first-row transition metals and their derivatives, with a particular focus on Ni, Co, and Fe-based compositions. Emphasizing their abundance and cost-effectiveness, we delve into strategies for constructing trimetallic water splitting electrocatalysts, encompassing oxides, hydroxides, phosphides, nitrides, chalcogenides, alloys, and composites, thereby shedding light on promising avenues for future research in this domain.
